# Supplementary material for: Assemblathon 2: evaluating de novo methods of genome assembly in three vertebrate species
Source: Gigascience. 2013 Jul 22;2:10. doi: 10.1186/2047-217X-2-10 (PMC3844414; doi:10.1186/2047-217X-2-10)
Supplement: Additional file 1 — Supplementary Data Description. Full details of the Illumina, Roche 454, and Pacific Biosciences sequencing data that were made available to participating teams. [file 2047-217X-2-10-S1.docx]

# Supplementary Data Description

Table of Contents

Supplementary Data Description 1

Bird Sequence Data 2

Illumina, Roche 454, and Pacific Biosciences sequence data 2

Roche 454 and Duke University Data 2

BGI Illumina HiSeq 2000 reads for parrot 4

UK Illumina HiSeq 2000 TrueSeq 3 (GC rich) reads 6

Duke Illumina GAIIx runs 7

Pacific Biosciences long reads 8

Fish Sequence Data 9

Broad Institute Sequence Data for Assemblathon 2 9

I. Fragment Libraries 9

II. Sheared Jumping Libraries 10

III. Fosmid Jumping Libraries 10

Library Coverage Estimates 11

Snake Sequence Data 12

Illumina sequencing data for Assemblathon 2 12

Short inserts: 12

Mate pairs: 12

# Bird Sequence Data

## Illumina, Roche 454, and Pacific Biosciences sequence data

### Roche 454 and Duke University Data

**Summary:** 19.7 Gb of sequence, 16X coverage total, read lengths 200–1000 bp

454 data were provided in the .sff file format, as unfiltered, untrimmed reads, with original 454 quality scores, in their original orientation. File names are varied, but in general are:

<species>_<animal name>_<library type>_<run, lane, or plate>_.sff

Library runs were split into 2, 4, or 8 lanes per plate. Each plate had the same coverage per run type. There were three library types (fragment sizes are calculated from .sff files):

1. 454 shotgun library, titanium runs: 600-800 bp insert size
   1. 30 files representing 2 lanes x 15 plates, lanes labeled 1=a and 2=b in the file name.
   2. Read length mode 456 bases
   3. Average quality score 31.2
   4. Sum of high quality reads 6.55 Gb.
2. 454 shotgun library, flexplus runs: ~1300 bp insert size
   1. 53 files representing 1/8 to 2 lanes x 11 plates
   2. Read length mode 736 bases
   3. Average quality score 26.5
   4. Sum of high quality reads 7.1 Gb.
3. 454 paired_end (5?--> <--3?), titanium runs: 3000, 8000, 20000 bp insert sizes
   1. 40 files representing multiple plates from the three types of 454 libraries
   2. 4 x 3Kb insert paired end (files 173 to 176)
   3. 8 x 8Kb insert paired end (files 13832 to 13839)
   4. 4 x 20Kb insert paired end (files 12212 to 12215).
   5. Initial 1/4 plate runs done at Roche 454; subsequent full plate runs done at Duke University.
   6. Read length mode 2 x 197-209 bases per end
   7. Average quality score 30.9-33.8
   8. Sum of high quality reads 4.77 Gb (1.90Gb for 3Kb, 1.05Gb for 8Kb, 1.82 for 20Kb)

**Library Coverage Averages (Assuming a 1.23 Gb genome)**

|-------------------------------+-----------+------------+----------|

| library name |insert size| Bases (Gb) | Coverage |

|-------------------------------+-----------+------------+----------|

| 1. budgie_shotgun_mrb | 600-800 | 6.55 | 5.32 |

| 2. budgie_mrb_roche_flexplus | ~1300 | 8.42 | 6.85 |

|-------------------------------+-----------+------------+----------|

| 3. budgie_mrb_3kbpe_173 | 3000 | 0.45 | 0.36 |

| 4. budgie_mrb_3kbpe_174 | 3000 | 0.56 | 0.46 |

| 5. budgie_mrb_3kbpe_175 | 3000 | 0.54 | 0.44 |

| 6. budgie_mrb_3kbpe_176 | 3000 | 0.35 | 0.28 |

| Total 3kb 1.90 | 1.54 |

| 7. budgie_mrb_8kbpe_13832 | 8000 | 0.14 | 0.11 |

| 8. budgie_mrb_8kbpe_13833 | 8000 | 0.14 | 0.12 |

| 9. budgie_mrb_8kbpe_13834 | 8000 | 0.13 | 0.11 |

| 10. budgie_mrb_8kbpe_13835 | 8000 | 0.13 | 0.11 |

| 11. budgie_mrb_8kbpe_13836 | 8000 | 0.13 | 0.10 |

| 12. budgie_mrb_8kbpe_13837 | 8000 | 0.14 | 0.11 |

| 13. budgie_mrb_8kbpe_13838 | 8000 | 0.13 | 0.10 |

| 14. budgie_mrb_8kbpe_13839 | 8000 | 0.12 | 0.10 |

| Total 8kb 1.05 | 0.85 |

| 15. budgie_mrb_20kbpe_12212 | 20000 | 0.35 | 0.29 |

| 16. budgie_mrb_20kbpe_12213 | 20000 | 0.57 | 0.46 |

| 17. budgie_mrb_20kbpe_12214 | 20000 | 0.43 | 0.35 |

| 18. budgie_mrb_20kbpe_12215 | 20000 | 0.47 | 0.38 |

| Total 20Kb 1.82 | 1.48 |

|-------------------------------+-----------+------------+----------|

###

###

### BGI Illumina HiSeq 2000 reads for parrot

**Summary:** 269 Gb of sequence, 219X coverage total, read lengths 90 and 151 bp)

These were raw reads, unfiltered, untrimmed, with original Illumina quality scores in their original orientation. A library was represented by one or more pairs of files, where the pair represents each read of the paired reads. Filenames included the sequencing-starting date, index name, the Illumina flowcell name and lane, the library name, and paired read identifier (1 or 2, where 1 is the first read of a pair and 2 is the second):

<date>_<index>_<flowcell>_<lane>_<library name>_<pair_read_ID>.fq.gz

A library could be split over multiple pairs of files - i.e. multiple lanes and/or flowcells.

There were two library types (fragment sizes are laboratory estimates!):

1. Short insert size, paired end (5?--> <--3?) TruSeq 3 chemistry:
   1. 220, 500, 800 bp insert sizes
   2. 2 x 151 base reads.
   3. 3 libraries
   4. 3 lanes
2. Large insert size, mate pairs (5?<-- -->3?) TruSeq 2 chemistry:
   1. 2000, 5000, 10000, 20000, 40000 bp insert sizes
   2. 2 X 90 base reads
   3. 7 libraries
   4. 8 lanes

Illumina mate pair reads were obtained from the circularization of a long DNA fragment in the 2–40kb size range. The junction site at which the fragment ends was joined are marked using a biotin molecule. This long fragment was sheared again and the resulting shorter fragment containing the junction site was isolated by means of the biotin molecule. TruSeq 2 chemistry does not sequence GC rich regions (> 75% across >100 bp), whereas TruSeq 3 does a better job at doing so.

**Library Coverage Estimates (Assuming a 1.23 Gb genome).**

|------------------------+------------+------------+----------|

| library name |insert size | Bases (Gb) | Coverage |

|------------------------+------------+------------+----------|

| 1. PARprgDAPDCAAPE | 220 | 48 | 39 |

| 2. PARprgDAPDIAAPE | 500 | 47 | 38 |

| 3. PARprgDAPDMAAPE | 800 | 43 | 35 |

|------------------------+------------+------------+----------|

| 4. PARprgDAPDWAAPE | 2000 | 15 | 12 |

| 5. PARprgDAPDWBAPE | 2000 | 31 | 26 |

| 6. PARprgDABDLBAPE | 5000 | 16 | 13 |

| 7. PARprgDABDLAAPE | 5000 | 18 | 15 |

| 8. PARprgDAADTAAPE | 10000 | 17 | 13 |

| 9. PARprgDAPDUAAPEI-12| 20000 | 16 | 13 |

| 10. PARprgDABDVAAPEI-6 | 40000 | 15 | 12 |

|------------------------+------------+------------+----------|

###

###

### UK Illumina HiSeq 2000 TrueSeq 3 (GC rich) reads

**Summary:** 66.76 Gb of sequence, 54.28X coverage total, read lengths 101 bp).

TrueSeq 3 reactions were done at Illumina UK to reduce the low read count of high GC rich regions (> 75% GC rich). Reactions were done on the BudgieILL library (see above) with 3 lanes, numbered 6, 7 and 8. Each lane has two files associated with it, designated read1 and read2, where _1 is the first read of a pair and _2 is the second. Each lane has two fastq files associated with it (which can be generated by running a script on the *qseq.txt files

that will be uploaded separately).

1. BudgieILL short insert size, paired end (5?--> <--3?): 400-600 bp insert size
   1. 6 files representing 3 lanes
   2. 2 x 101 bp reads

**Library Coverage Estimate (Assuming a 1.23 Gb genome):**

|------------------------------+------------+------------+----------|

| library name |insert size | Bases (Gb) | Coverage |

|------------------------------+------------+------------+----------|

| 1. BudgieILL (lanes 6-8) | 400-600 | 66.7 | 54.3 |

|------------------------------+------------+------------+----------|

### Duke University Illumina GAIIx runs

Two libraries were created, and a standard illumina GAIIx run was done at Duke University on 5 lanes, numbered 1, 2, 6, 7 and 8. Each lane had two fastq files associated with it (read1 or read2, where _1 is the first read of a pair and _2 is the second). The two libraries and the lanes in which these were sequenced are described below:

1. BudgieTUFT short insert size, paired end (5?--> <--3?): 400-600 bp insert size
   1. 2 files representing 1 lane (lane 6).
   2. 2 x 76 bp reads
2. BudgieILL short insert size, paired end (5?--> <--3?): 400-600 bp insert size
   1. 8 files representing 4 lanes (Lanes 1, 2, 7 and 8)
   2. 2 x 76 bp reads
   3. NOTE: Lanes 1, 2 were sequenced with a low-emulsion density chemistry,

in order to better sequence GC-rich regions.

|------------------------------+------------+------------+----------|

| library name |insert size | Bases (Gb) | Coverage |

|------------------------------+------------+------------+----------|

| 1. BudgieTUFT (lanes 6-8) | 400-600 | 1.8 | 1.5 |

| 2. BudgieILL (lanes 7-8) | 400-600 | 7.8 | 6.3 |

| 3. BudgieILL (lanes 1-2) | 400-600 | 9.9 | 8.1 |

| (low-emulsion density | | | |

| sequencing) | | | |

|------------------------------+------------+------------+----------|

### Pacific Biosciences long reads

Two libraries were generated:

1. 7 Kbp insert library (average read length ~1.2 Kbp)
2. 10 Kbp insert library (average read length ~3 Kbp, with 5% of the reads over 6–8 Kbp)

A total of 10X coverage of sequence was made.

#

#

# Fish Sequence Data

## Broad Institute Sequence Data for Assemblathon 2

The Broad Institute provided Illumina data for the *M. zebra* fish. Sequence was provided as raw reads, unfiltered, untrimmed, with original Illumina quality scores and in their original orientation.

Libraries were represented by one or more pairs of files, where the pair represents each read of the paired reads. Filenames include the Illumina flowcell name and lane, and paired read identifier (1 or 2, where 1 is the first read of a pair and 2 is the second). I.e.:

<flowcell>.<lane>.<pair_read_ID>.fastq.gz

803DNABXX.6.1.fastq.gz 803DNABXX.6.2.fastq.gz

A library may be split over multiple pairs of files - i.e., multiple lanes and/or flowcells. Fragment sizes are laboratory estimates.

### I. Fragment Libraries

2x 101 base reads.

Orientation: 1=fw, 2=rc

Library name: Solexa-38739

Estimated fragment size: 180 +/- 15

625E1AAXX.1.1.fastq 625E1AAXX.1.2.fastq

625E1AAXX.2.1.fastq 625E1AAXX.2.2.fastq

625E1AAXX.3.1.fastq 625E1AAXX.3.2.fastq

625E1AAXX.4.1.fastq 625E1AAXX.4.2.fastq

625E1AAXX.5.1.fastq 625E1AAXX.5.2.fastq

625E1AAXX.6.1.fastq 625E1AAXX.6.2.fastq

625E1AAXX.7.1.fastq 625E1AAXX.7.2.fastq

625E1AAXX.8.1.fastq 625E1AAXX.8.2.fastq

### II. Sheared Jumping Libraries

These libraries were generated using the Illumina protocol, which uses blunt-end ligation. They may contain a sizable population of non-jumps, which have the opposite orientation. They will also contain reads that are chimeric, caused by sequencing through the circularization junction

point. The proportion of non-jumps and chimeric reads varies from library to library.

2x 101 base reads.

Orientation: 1=rc, 2=fw

Library name: Solexa-50914

Estimated fragment size: 7000 +/- 700

803DNABXX.1.1.fastq 803DNABXX.1.2.fastq

Library name: Solexa-39450

Estimated fragment size: 2500 +/- 250

801KYABXX.2.1.fastq 801KYABXX.2.2.fastq

801KYABXX.3.1.fastq 801KYABXX.3.2.fastq

Library name: Solexa-50937

Estimated fragment size: 5000 +/- 500

803DNABXX.6.1.fastq 803DNABXX.6.2.fastq

Library name: Solexa-50902

Estimated fragment size: 9000 +/- 900

803DNABXX.2.1.fastq 803DNABXX.2.2.fastq

Library name: Solexa-51379

Estimated fragment size: 11000 +/- 1100

803DNABXX.8.1.fastq 803DNABXX.8.2.fastq

Library name: Solexa-39462

Estimated fragment size: 2500 +/- 250

801KYABXX.4.1.fastq 801KYABXX.4.2.fastq

### III. Fosmid Jumping Libraries

2x 101 base reads (2x 72 base reads after trimming)

Only bases 5-76 are genomic.

(Trim first 4 bases, keep next 72 bases, discard remaining bases)

Orientation: 1=fw, 2=rc

Library name: Solexa-46074

Estimated fragment size: 40000 +/- 4000

62F6HAAXX.1.1.fastq 62F6HAAXX.1.2.fastq

62F6HAAXX.2.1.fastq 62F6HAAXX.2.2.fastq

### Library Coverage Estimates

Assuming a 1 Gb genome.

|--------------+--------------+------------+--------------+----------|

| Type | Name | Reads | Bases | Coverage |

|--------------+--------------+------------+--------------+----------|

| fragment | Solexa-38739 | 597610332 | 60358643532 | 60 |

| sheared jump | Solexa-39450 | 492188542 | 49711042742 | 50 |

| sheared jump | Solexa-39462 | 217999666 | 22017966266 | 22 |

| sheared jump | Solexa-50902 | 147317752 | 14879092952 | 15 |

| sheared jump | Solexa-50914 | 158260012 | 15984261212 | 16 |

| sheared jump | Solexa-50937 | 143454662 | 14488920862 | 14 |

| sheared jump | Solexa-51379 | 114671088 | 11581779888 | 12 |

| fosmid jump | Solexa-46074 | 38364464 | 2762241408 | 2.8 |

|--------------+--------------+------------+--------------+----------|

| | Total | 1909866518 | 191783948862 | 192 |

|--------------+--------------+------------+--------------+----------|

# Snake Sequence Data

## Illumina sequencing data for Assemblathon 2

### Short inserts:

* Paired reads with orientation --> <--

* Two Genome Analyzer IIx runs with 8 lanes each

* 120 bp reads length and approx. 400 bp insert size

### Mate pairs:

The mate pair data set comprises two Genome Analyzer IIx flow cells. Three libraries were sequenced with different insert sizes using each of the two protocols. Illumina sequenced 2–3 lanes from each library. *Protocol A* is a protocol in research and kits were not currently available to the public at the time data was generated for the Assemblathon 2.

Flowcell 1: 110405_EAS192_0222_FC70N35AAXX

Lane 1 Protocol A Mate Pair 2 kb

Lane 2 Protocol A Mate Pair 2 kb

Lane 3 Protocol A Mate Pair 2 kb

Lane 4 Protocol A Mate Pair 10 kb (previously listed incorrectly as 4 kb)

Lane 5 Protocol A Mate Pair 10 kb (previously listed incorrectly as 4 kb)

Lane 6 Protocol A Mate Pair 10 kb (previously listed incorrectly as 4 kb)

Lane 7 Protocol A Mate Pair 4 kb (previously listed incorrectly as 10 kb)

Lane 8 Protocol A Mate Pair 4 kb (previously listed incorrectly as 10 kb)

Illumina mate pair reads are obtained from the circularization of a long DNA fragment in the 2–10 kb size range. The junction site at which the fragment ends are joined are marked using a biotin molecule. This long fragment is sheared again and the resulting shorter fragment containing the junction site is isolated by means of the biotin molecule. Sequencing this fragment from both ends yields paired-end reads with a genomic distance matching the initial fragment size.

The Protocol A mate pairs have a read length of 100 bp. Most reads will contain the biotin junction site, but it is marked by an adapter sequence which is "CAGTACTG". This allows users of the data to split the reads at the junction site and thus avoid chimeric reads.

Note that all these reads have an outward facing genomic orientation (<-- -->) which is different from the short insert protocol that yields inward facing reads.
