# Supplementary material for: Assemblathon 2: evaluating de novo methods of genome assembly in three vertebrate species
Source: Gigascience. 2013 Jul 22;2:10. doi: 10.1186/2047-217X-2-10 (PMC3844414; doi:10.1186/2047-217X-2-10)
Supplement: Additional file 2 — Supplementary Results. Additional figures and tables to accompany the main text. [file 2047-217X-2-10-S2.docx]

# Supplementary results for Assemblathon 2 paper

**Table S1. Different assembly IDs used in Assemblathon 2.**

While the Assemblathon 2 entries were being assessed, an anonymous identifier was used to refer to all assemblies. This consisted of a species description followed by a 2–3 character code. These have since been replaced with more human-readable identifiers, but as other publications may refer to the older identifiers, we have included them here. Assembly names with a ‘C’ or ‘E’ suffix refer to ‘competition’ or ‘evaluation’ entries. For the newer assembly IDs, evaluation entries are indicated by the use of one or two asterisks appended to the assembly ID: one asterisk for the first, or only, evaluation entry and two asterisks to refer to the second evaluation entry.

| **Team name** | **New assembly ID prefixes** | **Old assembly IDs** |
| --- | --- | --- |
| ABL | ABL | bird 15C |
| ABySS | ABYSS | fish 7C, snake 9C |
| Allpaths | ALLP | bird 11C, fish 6C |
| BCM-HGSC | BCM | bird 2C, bird 3E, fish 1C, snake 1C |
| CBCB | CBCB | bird 9C |
| CoBiG2 | COBIG | bird 8C |
| CRACS | CRACS | snake 10C |
| CSHL | CSHL | fish 12C, fish 14E, fish 15E |
| CTD | CTD | fish 2E, fish 9C, fish 10E |
| Curtain | CURT | snake 3C |
| GAM | GAM | snake 4C |
| IOBUGA | IOB | fish 13C, fish 16E |
| MLK Group | MLK | bird 5C |
| Meraculous | MERAC | bird 6C, fish 8C, snake 6C |
| Newbler-454 | NEWB | bird 7C |
| Phusion | PHUS | bird 1C, snake 5C |
| PRICE | PRICE | snake 12C |
| Ray | RAY | bird 4C, fish 4C, snake 2C |
| SGA | SGA | bird 10C, fish 3C, snake 7C |
| SOAPdenovo | SOAP | bird 12C, bird 13E, bird 14E, fish 11E, snake 11C |
| Symbiose | SYMB | fish 5C, snake 8C |

**Table S2. Details of principle assembly software and CPU/RAM requirements of different assembly pipelines.**

Instructions to run assemblers are included in Supplementary Methods for some teams.

| **Team name** | **Principle Software Used** | **CPU/RAM requirements** |
| --- | --- | --- |
| ABL | HyDA | 512 GB RAM machine with 48 cores. Runtime: 14 hours. |
| ABySS | ABySS v1.3.0 and Anchor | ABySS: 48 core-cluster for the single-end stage, and 12 cores for the paired-end and scaffolding stages, each with 4 GB RAM. Runtime was ~4 h for the single-end stage, and 13 h for the paired-end stage, then another three days for the final scaffolding stage.  Anchor: Same cluster as above, using 1–100 cores for the various stages. Total runtime was approximately 13 h. |
| Allpaths | ALLPATHS-LG | 48 core server with 512 GB RAM, with a runtime of ~151–215 h (depending on species). |
| BCM-HGSC | SeqPrep (version: a1e1d38), KmerFreq, Quake (v0.2), BWA, Newbler (v2.3), ALLPATHS-LG (version: allpathslg-37405), Atlas-Link, Atlas-GapFill, Phrap, CrossMatch, Velvet, BLAST, and BLASR | Estimated max RAM: 300–500 GB (depending on species). Estimated running time: 3.5 weeks; using a single node with 1 TB RAM and 32 CPUs, as well as a cluster of 100 cores each with 16 GB RAM.  Gap filling step used a cluster of 100–600 cores (depending on species), each with 16 GB RAM and required a run time of 90 h. |
| CBCB | Celera assembler v7 and PacBio Corrected Reads (PBcR) | Runtime of 6.75 days for PacBio read correction and 9.5 days for assembly. Serial steps were executed on 32 core head node with 256 GB RAM. Parallel jobs were distributed across 60 nodes, with 16 cores and 32 GB RAM each. |
| CoBiG2 | 4Pipe4 pipeline, Seqclean (version: 2011-02-22), Mira (v3.2.1), Bambus2 | DELL Power Edge R710, CPU: 2x Intel Xeon E5520, RAM: 64 GB, Runtime of 24 h. |
| CRACS | ABySS, SSPACE, Bowtie, and FASTX | Single 6-core AMD Opteron™ processor (2100 MHz) with 128 GB of RAM. The approximate total amount of computation time required to generate the assembly was 300 h. |
| CSHL | Metassembler, ALLPATHS, SOAPdenovo | Metassembler: <3 h runtime and <50 GB RAM for the pairwise alignment. Computing the CE statistic required ~10 h and 50 GB RAM, dominated by aligning the reads to the assemblies to determine placement. Evaluating the alignments and patching the assemblies required ~1 h.  ALLPATHS:  48 available CPUs, 945 h of elapsed time, and 456 GB RAM memory usage peak  SOAPdenovo:  ~1 day, 100 GB RAM, 48 cores for FLASH, and Quake  ~1 day for the basic assembly  ~1 day to align the mates, filter failed mates, remove PCR duplicates  ~1 day to improve the assembly with the corrected mates. |
| CTD | Unspecified | 48 GB RAM |
| Curtain | SOAPdenovo (v1.05), fastx_toolkit (v0.0.6), bwa (v0.5.8a), samtools (v0.1.17), velvet (v1.1.06), curtain (v0.2.3-BETA) | 14 h on 1 machine with 170 GB RAM, plus 11 h on 20 machines with 60 GB RAM. |
| GAM | GAM, CLC and ABySS | CLC: one server, 8 cores, 128 Gb RAM, half a day runtime.  ABySS, cluster with 6 nodes, 8 cores per node, one day runtime.  GAM: one server, 8 cores, 128Gb RAM, half a day runtime.  SSPACE: single CPU, 1 h runtime. |
| IOBUGA | ALLPATHS-LG (38293) and SOAPdenovo (1.05) | 32 CPU machine, 512 GB RAM. Runtime: ~ 120 h for ALLPATH-LG and  48 h for SOAPdenovo. |
| MLK Group | ABySS | 672 core cluster, 1.2 TB RAM distributed, non-parallel steps done on 256 GB RAM machine and single node. SGA steps done on local workstation with 36 GB RAM. |
| Meraculous | meraculous | 500 core cluster with 8 GB RAM per core. Runtime: 20 hours. Single core machine with ~100 GB RAM. Runtime 10 h. |
| Newbler-454 | Newbler (R&D version, post2.8_v20110815). Run with options "-large -scaffold -het -sio -cpu 12" | Shared memory machine, 12 cores used, 130 GB RAM, run time of 18 h. |
| Phusion | Phusion2, SOAPdenovo, SSPACE | 160GB RAM for 72 h, 100 cores with 4GB RAM for 2 h. |
| PRICE | PRICE | Run on various 8–64 core machines with 16–256 GB RAM. |
| Ray | Ray (version 1.7 with some modifications, see:<https://github.com/sebhtml/assemblathon-2-ray>**)** | Version: 32 computers, 8 cores per computer, 24 GB RAM per computer. Approx. running time: 36–72 h (depending on species). |
| SGA | SGA | Total CPU time: 1,000–1,900 h (depending on species). Total wall clock time: 174 h. Peak memory usage: 34–50 GB RAM (depending on species). |
| SOAPdenovo | SOAPdenovo | 110–150 GB RAM (peak), depending on species, 24–32 CPUs (depending on species). Runtime 48–72 h (depending on species). |
| Symbiose | Monument (for paired-end assembly), SSPACE (for mate-pair scaffolding in snake), SuperScaffolder (for mate-pair scaffolding in fish), and GapCloser (for GapClosing) | Computational resources: 40 cores on 5 nodes with 140 GB RAM (max RAM usage not recorded)  Runtimes:  Indexing: ~1 day (40 cores / 5 nodes). Paired-end assembly: ~1 day (16 cores / 1 node). Two rounds of scaffolding and gap-filling: ~1 day (8 cores / 1 node). |

**Table S3. Availability of software used for assemblies.**

| **Assembly software** | **URL** | **Reference (if published)** |
| --- | --- | --- |
| 4Pipe4 pipeline | <https://github.com/StuntsPT/4Pipe4/commit/a1808cecce7025a3fb90d64a337ccbe08619267a> |  |
| ABySS | [http://www.bcgsc.ca/platform/bioinfo/software/abyss](http://www.bcgsc.ca/platform/bioinfo/software/abyss),) | [1] |
| ALLPATHS-LG | http://www.broadinstitute.org/software/allpaths-lg/blog/ | [2] |
| Anchor | http://www.bcgsc.ca/platform/bioinfo/software/anchor |  |
| Atlas-GapFill | <https://www.hgsc.bcm.edu/content/atlas-gapfill> | [3] |
| Atlas-Link | <https://www.hgsc.bcm.edu/content/Atlas-Link> | [4] |
| Bambus2 | <http://www.cbcb.umd.edu/software/bambus/> |  |
| BLASR | <http://www.pacificbiosciences.com/products/software/algorithms/> | [5] |
| BLAST | http://blast.ncbi.nlm.nih.gov/ | [6] |
| Bowtie | <http://bowtie-bio.sourceforge.net/index.shtml> | [7] |
| BWA | <http://bio-bwa.sourceforge.net/> | [8] |
| Celera | http://wgs-assembler.sourceforge.net/ | [9] |
| CLC Genomics Workbench de novo assembler | http://clcbio.com |  |
| Curtain | <http://code.google.com/p/curtain/> |  |
| FASTX | <http://hannonlab.cshl.edu/fastx_toolkit/> |  |
| GAM (Genomic Assemblies Merger) | https://github.com/vice87/gam-ngs | [10] |
| HyDA | http://compbio.cs.wayne.edu/software/hyda/ |  |
| KmerFreq (part of SOAPdenovo) | http://soap.genomics.org.cn/soapdenovo.html | [11] |
| Meraculous | ftp://ftp.jgi-psf.org/pub/JGI_data/meraculous/ | [12] |
| Metassembler | http://sourceforge.net/apps/mediawiki/metassembler/index.php?title=Metassembler |  |
| MIRA | <http://www.chevreux.org/projects_mira.html> |  |
| Monument |  | [13] |
| Newbler | <http://454.com/products/analysis-software/index.asp> | [14] |
| PBcR | http://www.cbcb.umd.edu/software/PBcR/ | [15] |
| Phrap & Crossmatch | <http://www.phrap.org/> |  |
| Phusion2 | ftp://ftp.sanger.ac.uk/pub/zn1/phusion2/ | [16] |
| PRICE | http://derisilab.ucsf.edu/software/price/ | [17] |
| Quake | <http://www.cbcb.umd.edu/software/quake/> | [18] |
| Ray | http://denovoassembler.sourceforge.net | [19] |
| SAMtools | <http://samtools.sourceforge.net/> | [20] |
| Seqclean | <http://sourceforge.net/projects/seqclean/files/seqclean-x86_64.tgz> |  |
| SeqPrep | https://github.com/jstjohn/SeqPrep |  |
| SGA | <http://github.com/jts/sga> | [21] |
| SOAPdenovo | http://soap.genomics.org.cn/soapdenovo.html | [11] |
| SSPACE | http://www.baseclear.com/landingpages/sspacev12/ | [22] |
| Velvet | [http://www.ebi.ac.uk/~zerbino/velvet/](http://www.ebi.ac.uk/%7Ezerbino/velvet/) | [23] |

**Table S4. Summary of available transcript and RefSeq data for bird, fish, and snake.**

Numbers in parentheses indicate partial length mRNAs. Data taken from release 192.0 of GenBank, accessed from: http://www.ncbi.nlm.nih.gov/nucleotide/

| **Species** | **Number of mRNAs** | **Number of RefSeq entries** |
| --- | --- | --- |
| Bird (*Melopsittacus undulatus*) | 26 (15) | 1 |
| Fish (*Maylandia zebra*) | 27 (22) | 0 |
| Snake (*Boa constrictor constrictor*) | 0 | 0 |

**Table S5.** **CEGMA bird results: total number of all CEGs present in all bird assemblies.**

Results in 3rd column reflect the numbers in the 2nd column as a percentage of the 442 CEGs that were found across all bird assemblies. Final column shows results for a subset of 248 CEGs which are the most highly conserved CEGs, and which tend to occur as single copy genes.

| **Assembly** | **Number of 458 CEGs present in assembly** | **% of 442 CEGs present across all bird assemblies** | **Number of 248 highly conserved CEGs present** |
| --- | --- | --- | --- |
| PHUS | 391 | 88.5% | 176 |
| BCM | 420 | 95.0% | 197 |
| BCM* | 420 | 95.0% | 197 |
| RAY | 404 | 91.4% | 190 |
| MLK | 401 | 90.7% | 181 |
| MERAC | 393 | 88.9% | 189 |
| NEWB | 380 | 86.0% | 179 |
| CBCB | 403 | 91.2% | 197 |
| SGA | 371 | 83.9% | 169 |
| ALLP | 408 | 92.3% | 199 |
| SOAP | 416 | 94.1% | 202 |
| SOAP* | 415 | 93.9% | 202 |
| SOAP** | 412 | 93.2% | 201 |
| ABL | 229 | 51.8% | 61 |

* or ** refers to the 1^st^ or 2^nd^ evaluation assemblies that were submitted by some teams.

**Table S6.** **CEGMA fish results: total number of all CEGs present in all fish assemblies.**

Results in 3rd column reflect the numbers in the 2nd column as a percentage **o**f the 455 CEGs that were found across all fish assemblies. The final column shows results for a subset of 248 CEGs which are the most highly conserved CEGs, and which tend to occur as single copy genes.

| **Assembly** | **Number of 458 CEGs present in assembly** | **% of 455 CEGs present across all fish assemblies** | **Number of 248 highly conserved CEGs present** |
| --- | --- | --- | --- |
| BCM | 434 | 95.4% | 228 |
| CTD* | 169 | 37.1% | 25 |
| SGA | 423 | 94.9% | 207 |
| RAY | 435 | 95.6% | 210 |
| SYM | 428 | 94.1% | 221 |
| ALLP | 430 | 94.5% | 225 |
| ABYSS | 431 | 94.7% | 224 |
| MERAC | 426 | 93.6% | 216 |
| CTD | 350 | 76.9% | 103 |
| CTD** | 207 | 45.5% | 41 |
| SOAP* | 436 | 95.8% | 225 |
| CSHL | 436 | 95.8% | 227 |
| IOB | 387 | 85.1% | 163 |
| CSHL* | 436 | 95.8% | 227 |
| CSHL** | 307 | 67.5% | 86 |
| IOB* | 83 | 18.2% | 16 |

* or ** refers to the 1^st^ or 2^nd^ evaluation assemblies that were submitted by some teams.

**Table S7.** **CEGMA snake results: total number of all CEGs present in all snake assemblies.**

Results in 3rd column reflect the numbers in the 2nd column as a percentage of the 454 CEGs that were found across all snake assemblies. The final column shows results for a subset of 248 CEGs which are the most highly conserved CEGs, and which tend to occur as single copy genes.

| **Assembly** | **Number of 458 CEGs present in assembly** | **% of 454 CEGs present across all snake assemblies** | **Number of 248 highly conserved CEGs present** |
| --- | --- | --- | --- |
| BCM | 434 | 95.6% | 214 |
| RAY | 422 | 93.0% | 194 |
| CURT | 360 | 79.3% | 91 |
| GAM | 415 | 91.4% | 157 |
| PHUS | 435 | 95.8% | 214 |
| MERAC | 430 | 94.7% | 217 |
| SGA | 433 | 95.4% | 218 |
| SYMB | 436 | 96.0% | 209 |
| ABYSS | 429 | 94.5% | 208 |
| CRACS | 438 | 96.5% | 211 |
| SOAP | 428 | 94.3% | 209 |

**Table S8.** **Using validated fosmid regions (VFRs) to assess short-range accuracy in bird assemblies.**

Results from 86 VFRs, producing 988 VFR fragments of 1,000 nt and 988 pairs of VFR ‘tags’ (the end 100 nt of each fragment). The expected distance between start coordinates of VFR tags = 900 nt. Tag pairs are deemed to have mapped correctly if the distance between them is 898–902 nt.

| **Assembly** | **Number of pairs of VFR tags that both map to the same scaffold** | **Number of pairs of VFR tags that map uniquely at correct distance apart (898–902 nt)** | **% of uniquely mapped tag pairs that map at correct distance apart** | **Extremes of mismapping (lowest and highest distances in nt)** |
| --- | --- | --- | --- | --- |
| PHUS | 815 | 557 | 89.1% | 702–41,949 |
| BCM | 890 | 713 | 92.6% | 882–2,780 |
| RAY | 896 | 699 | 91.6% | 746–4,175 |
| MLK | 857 | 544 | 93.8% | 804–2,780 |
| MERAC | 840 | 746 | 91.9% | 800–7,815 |
| NEWB | 849 | 733 | 91.2% | 871–2,780 |
| CBCB | 897 | 744 | 91.4% | 855–8,002 |
| SGA | 795 | 709 | 91.6% | 713–34,915 |
| ALLP | 881 | 758 | 92.6% | 875–43,292 |
| SOAP | 876 | 720 | 90.1% | 709–4,805 |
| ABL | 337 | 332 | 98.5% | 893–952 |

**Table S9.** **Using validated fosmid regions (VFRs) to assess short-range accuracy in snake assemblies.**

Results from 56 VFRs, producing 350 VFR fragments of 1,000 nt and 988 pairs of VFR ‘tags’ (the end 100 nt of each fragment). The expected distance between start coordinates of VFR tags = 900 nt. Tag pairs are deemed to have mapped correctly if the distance between them is 898–902 nt.

| **Assembly** | **Number of pairs of VFR tags that both map to the same scaffold** | **Number of pairs of VFR tags that map uniquely at correct distance apart (898–902 nt)** | **% of uniquely mapped tag pairs that map at correct distance apart** | **Extremes of mismapping (lowest and highest distances in nt)** |
| --- | --- | --- | --- | --- |
| BCM | 278 | 240 | 90.2% | 835–1,864 |
| RAY | 311 | 253 | 95.5% | 860–2,973 |
| CURT | 272 | 220 | 87.6% | 835–46,813 |
| GAM | 236 | 200 | 88.9% | 815–1,022 |
| PHUS | 336 | 247 | 89.2% | 653–2,070 |
| MERAC | 319 | 263 | 95.3% | 875–912 |
| SGA | 323 | 265 | 94.3% | 860–920 |
| SYMB | 300 | 211 | 87.9% | 673–1,364 |
| ABYSS | 323 | 267 | 94.7% | 878–2,206 |
| CRACS | 304 | 253 | 90.7% | 855–3,472 |
| SOAP | 334 | 227 | 90.4% | 830–4,858 |

**Figure S1. Contig NG50 length for all competitive assemblies.**


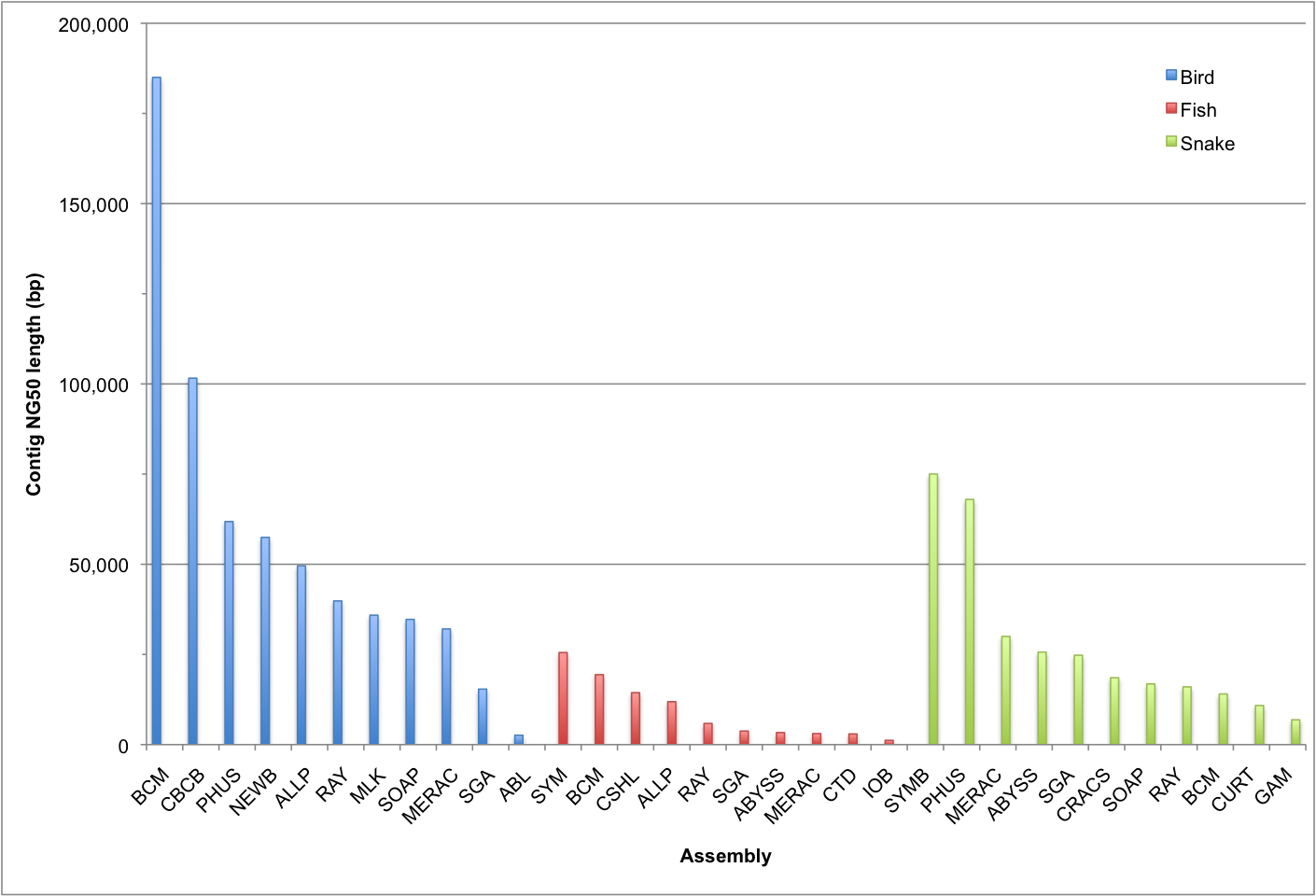


**Figure S2. Relationship between scaffold NG50 length and contig NG50 length.**

P-values from correlation coefficients: bird: *P* = 0.0587, fish: *P* = 0.0039, snake: *P* = 0.0398


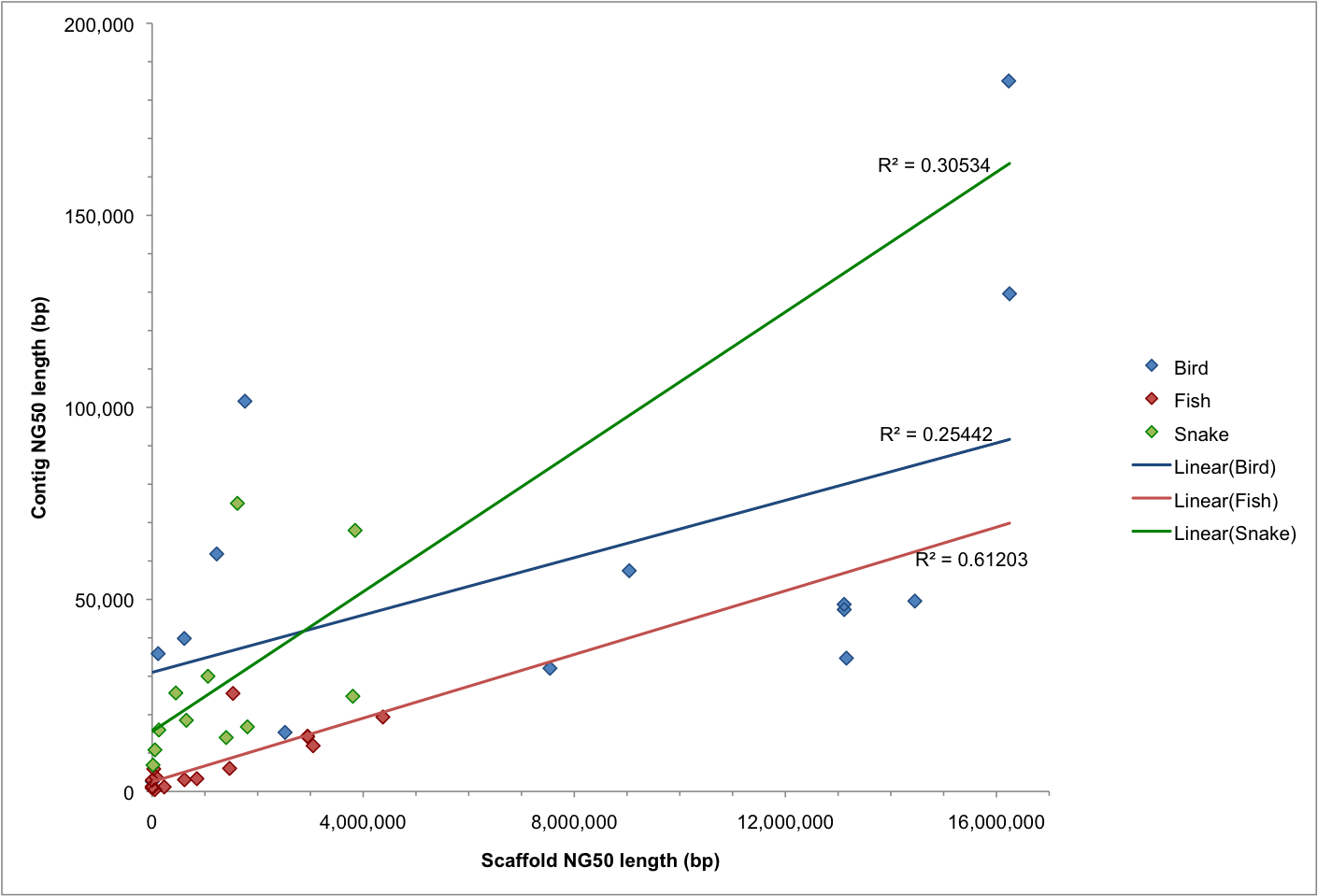


**Figure S3.** **NG50 scaffold length distribution in bird assemblies and the fraction of the fish genome represented by gene-sized scaffolds.**

Primary Y-axis (red) shows NG50 length for fish assemblies: the N50 scaffold length that captures 50% of the estimated genome size (~1.6 Gbp). Secondary Y-axis (blue) shows percentage of estimated genome size that is represented by scaffolds ≥25 Kbp (the average length of a vertebrate gene).


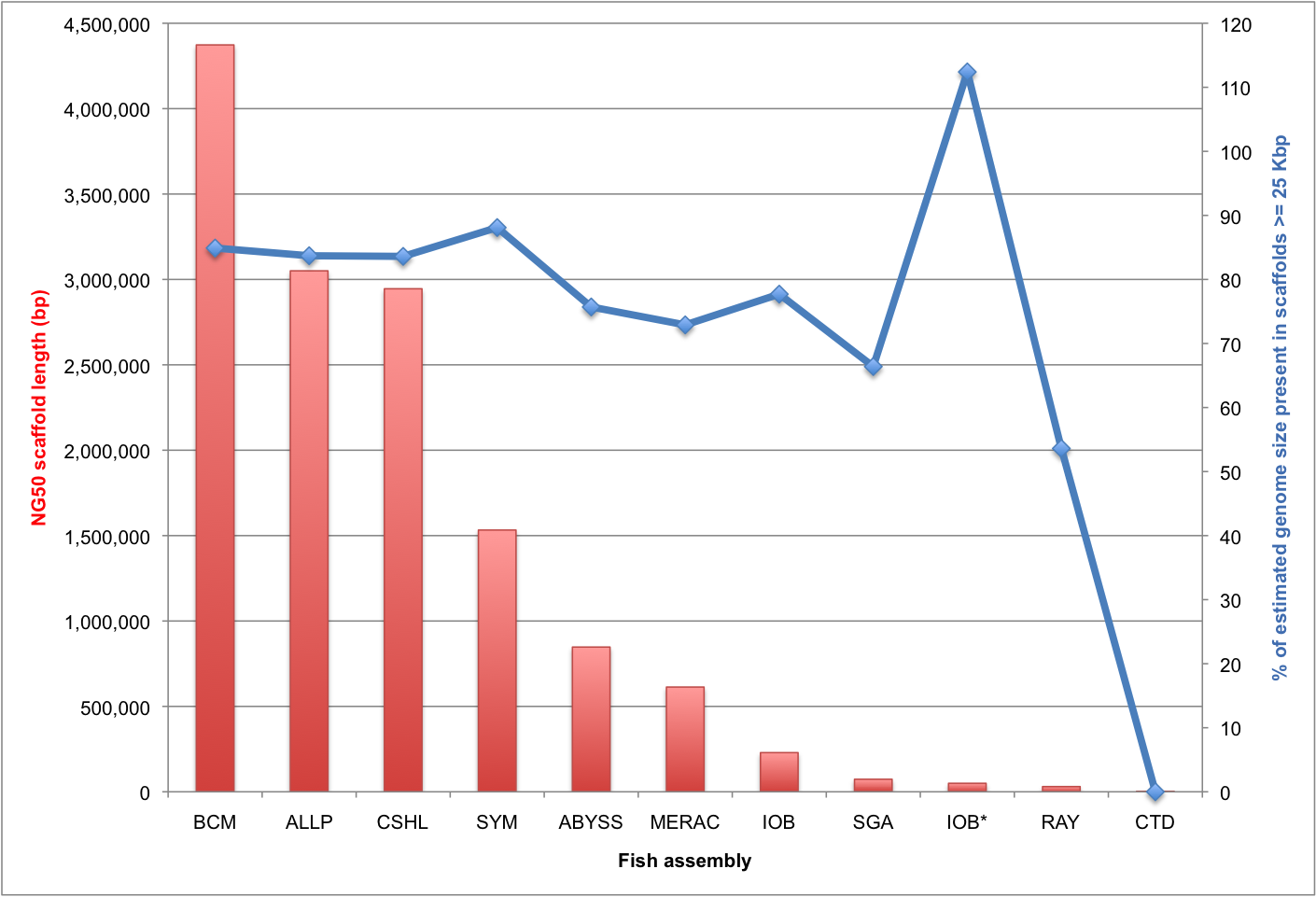


**Figure S4.** **NG50 scaffold length distribution in bird assemblies and the fraction of the snake genome represented by gene-sized scaffolds.**

Primary Y-axis (red) shows NG50 length for snake assemblies: the N50 scaffold length that captures 50% of the estimated genome size (~1.0 Gbp). Secondary Y-axis (blue) shows percentage of estimated genome size that is represented by scaffolds ≥25 Kbp (the average length of a vertebrate gene).


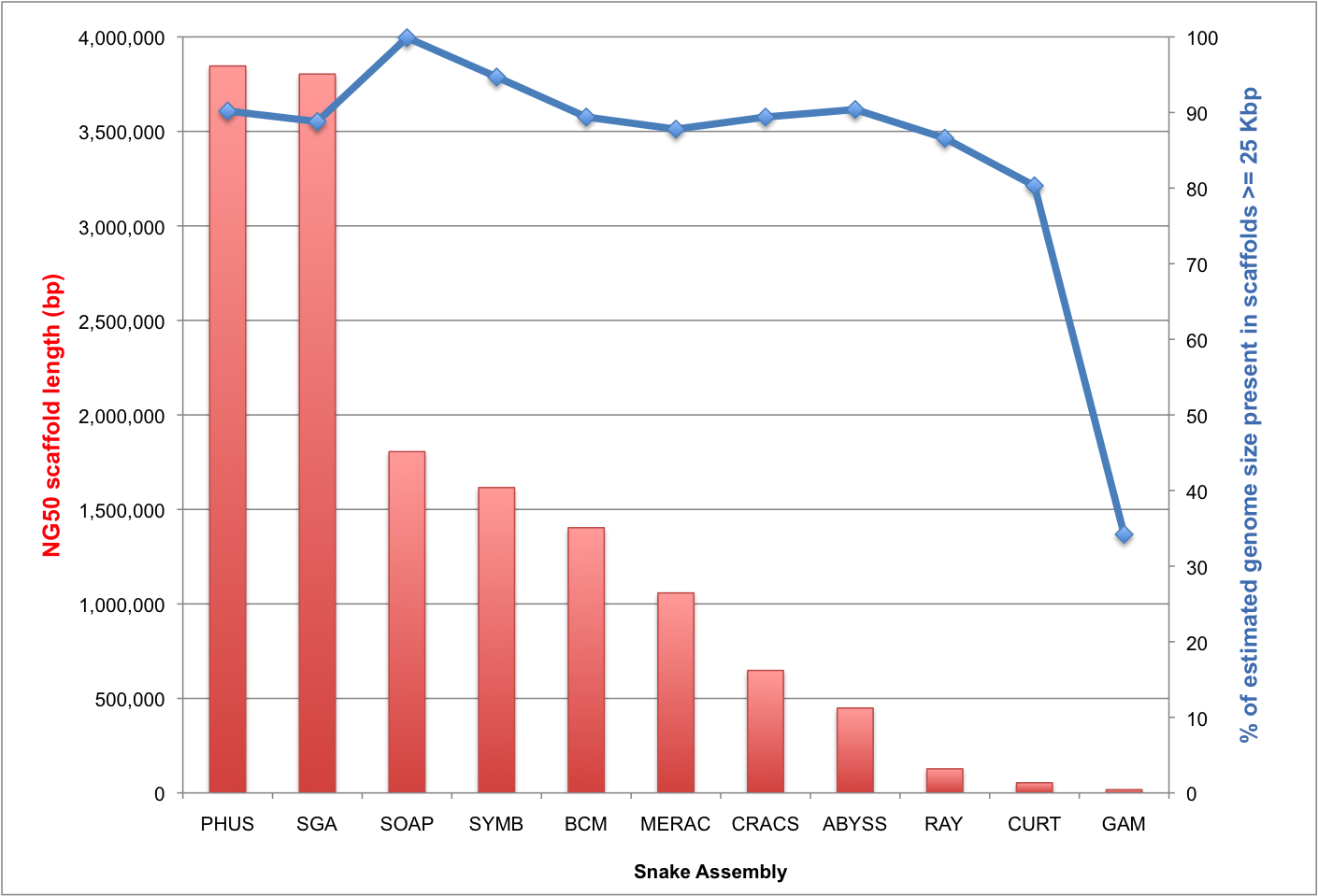


**Figure S5.: Alignment of snake predicted CEGMA proteins for the core gene family KOG3372.**

Alignment made using T-COFFEE program with default parameters. The initial set of proteins predicted by CEGMA are aligned to the underlying HMMER profile for each core gene, and only those that span at least 70% of the alignment are considered ‘full-length’ and retained.

CLUSTAL FORMAT for T-COFFEE Version_5.31 [http://www.tcoffee.org], CPU=3.13 sec, SCORE=96, Nseq=11, Len=205

ABYSS MNTVLTRANSLFAFSLSVMAALTFGCFITTAFKERTVPVSIAVSRVML-------KNVED

BCM MNTVLTRANSLFAFSLSVMAALTFGCFITTAFKERTVPVSIAVSRVML-------KNVED

CRACS MNTVLTRANSLFAFSLSVMAALTFGCFITTAFKERTVPVSIAVSRVML-------KNVED

CURT MNTVLTRANSLFAFSLSVMAALTFGCFITTAFKERTVPVSIAVSRVML-------KNVED

GAM MNTVLTRANSLFAFSLSVMAALTFGCFITTAFKERTVPVSIAVSRVMLFYEVRKIKNVED

MERAC MNTVLTRANSLFAFSLSVMAALTFGCFITTAFKERTVPVSIAVSRVML-------KNVED

PHUS MNTVLTRANSLFAFSLSVMAALTFGCFITTAFKERTVPVSIAVSRVML-------KNVED

RAY MNTVLTRANSLFAFSLSVMAALTFGCFITTAFKERTVPVSIAVSRVML-------KNVED

SGA MNTVLTRANSLFAFSLSVMAALTFGCFITTAFKERTVPVSIAVSRVML-------KNVED

SYMB MNTVLTRANSLFAFSLSVMAALTFGCFITTAFKERTVPVSIAVSRVMLFYEVRKIKNVED

SOAP MNTVLTRANSLFAFSLSVMAALTFGCFITTAFKERTVPVSIAVSRVML-------KNVED

************************************************ *****

ABYSS FTGPGERSDLGIITFNISANILYYKHSSLFPNIFDWNVKQLFLYLSAEYSTKNN------

BCM FTGPGERSDLGIITFNISANILYYKHSSLFPNIFDWNVKQLFLYLSAEYSTKNN------

CRACS FTGPGERSDLGIITFNISANILYYKHSSLFPNIFDWNVKQLFLYLSAEYSTKNN------

CURT FTGPGERSDLGIITFNISANILYYKHSSLFPNIFDWNVKQLFLYLSAEYSTKNN------

GAM FTGPGERSDLGIITFNISANILYYKHSSLFPNIFDWNVKQLFLYLSAEYSTKNNLPHTHI

MERAC FTGPGERSDLGIITFNISANILYYKHSSLFPNIFDWNVKQLFLYLSAEYSTKNN------

PHUS FTGPGERSDLGIITFNISANILYYKHSSLFPNIFDWNVKQLFLYLSAEYSTKNN------

RAY FTGPGERSDLGIITFNISANILYYKHSSLFPNIFDWNVKQLFLYLSAEYSTKNN------

SGA FTGPGERSDLGIITFNISANILYYKHSSLFPNIFDWNVKQLFLYLSAEYSTKNN------

SYMB FTGPGERSDLGIITFNISANILYYKHSSLFPNIFDWNVKQLFLYLSAEYSTKNN------

SOAP FTGPGERSDLGIITFNISANILYYKHSSLFPNIFDWNVKQLFLYLSAEYSTKNN------

******************************************************

ABYSS ---ALNQVVLWDKIILRGDDPNLLLKDMKSKYFFFDDGNGLKGNRNVTLTLSWNVVPNAG

BCM ---ALNQVVLWDKIILRGDDPNLLLKDMKSKYFFFDDGNGLKGNRNVTLTLSWNVVPNAG

CRACS ---ALNQVVLWDKIILRGDDPNLLLKDMKSKYFFFDDGNGLKGNRNVTLTLSWNVVPNAG

CURT ---ALNQVVLWDKIILRGDDPNLLLKDMKSKYFFFDDGNGLKGNRNVTLTLSWNVVPNAG

GAM YGHALNQVVLWDKIILRGDDPNLLLKDMKSKYFFFDDGNGLK------------------

MERAC ---ALNQVVLWDKIILRGDDPNLLLKDMKSKYFFFDDGNGLKGNRNVTLTLSWNVVPNAG

PHUS ---ALNQVVLWDKIILRGDDPNLLLKDMKSKYFFFDDGNGLKGNRNVTLTLSWNVVPNAG

RAY ---ALNQVVLWDKIILRGDDPNLLLKDMKSKYFFFDDGNGLKGNRNVTLTLSWNVVPNAG

SGA ---ALNQVVLWDKIILRGDDPNLLLKDMKSKYFFFDDGNGLKGNRNVTLTLSWNVVPNAG

SYMB ---ALNQVVLWDKIILRGDDPNLLLKDMKSKYFFFDDGNGLKGNRNVTLTLSWNVVPNAG

SOAP ---ALNQVVLWDKIILRGDDPNLLLKDMKSKYFFFDDGNGLKGNRNVTLTLSWNVVPNAG

***************************************

ABYSS ILPLVTGAGHISVPFPDTYKMTKSY

BCM ILPLVTGAGHISVPFPDTYKMTKSY

CRACS ILPLVTGAGHISVPFPDTYKMTKSY

CURT ILPLVTGAGHISVPFPDTYKMTKSY

GAM -------------------------

**Figure S6.** **Correlation between use of two different CEGs datasets.**

Assemblies which contain more full-length core genes from the set of 458 CEGs, also contain more full-length core genes from the set of 248 CEGs which represent the most highly-conserved, least-paralogous CEGs. All correlations are highly statistically significant (*P* < 0.000001).


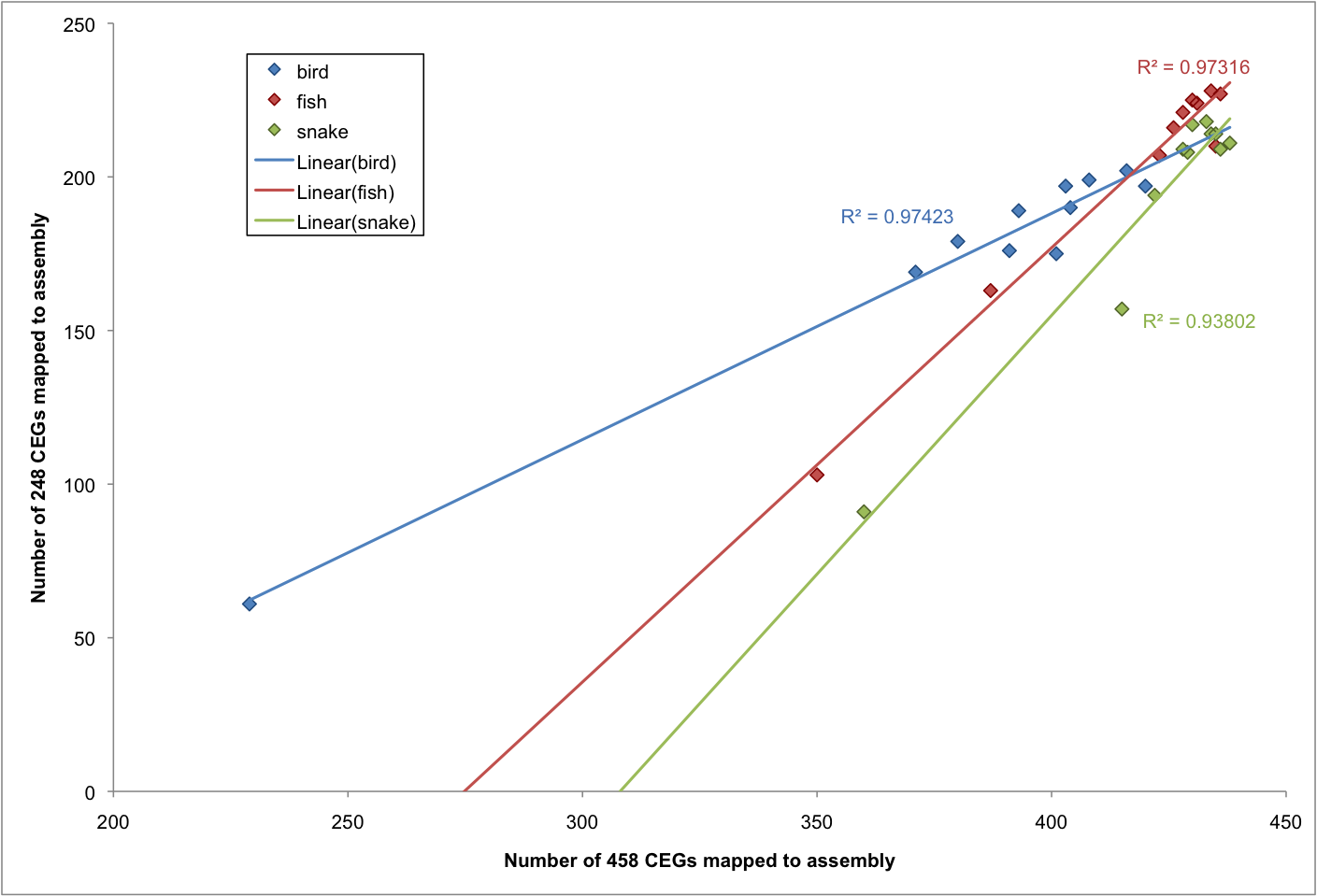


**Figure S7.** **Some core genes are present as partial matches in assemblies.**

Results from the CEGMA analysis of the 248 CEGs dataset includes details of how many CEGs match at full-length or only partially. The fraction of the alignment of a predicted protein to the HMMER profile can range from 20–100%. If this fraction exceeds 70% the protein is classed as a full-length CEG, otherwise it is classified as partial. In both cases, the predicted protein must also exceed a pre-determined cut-off score (see [24]).


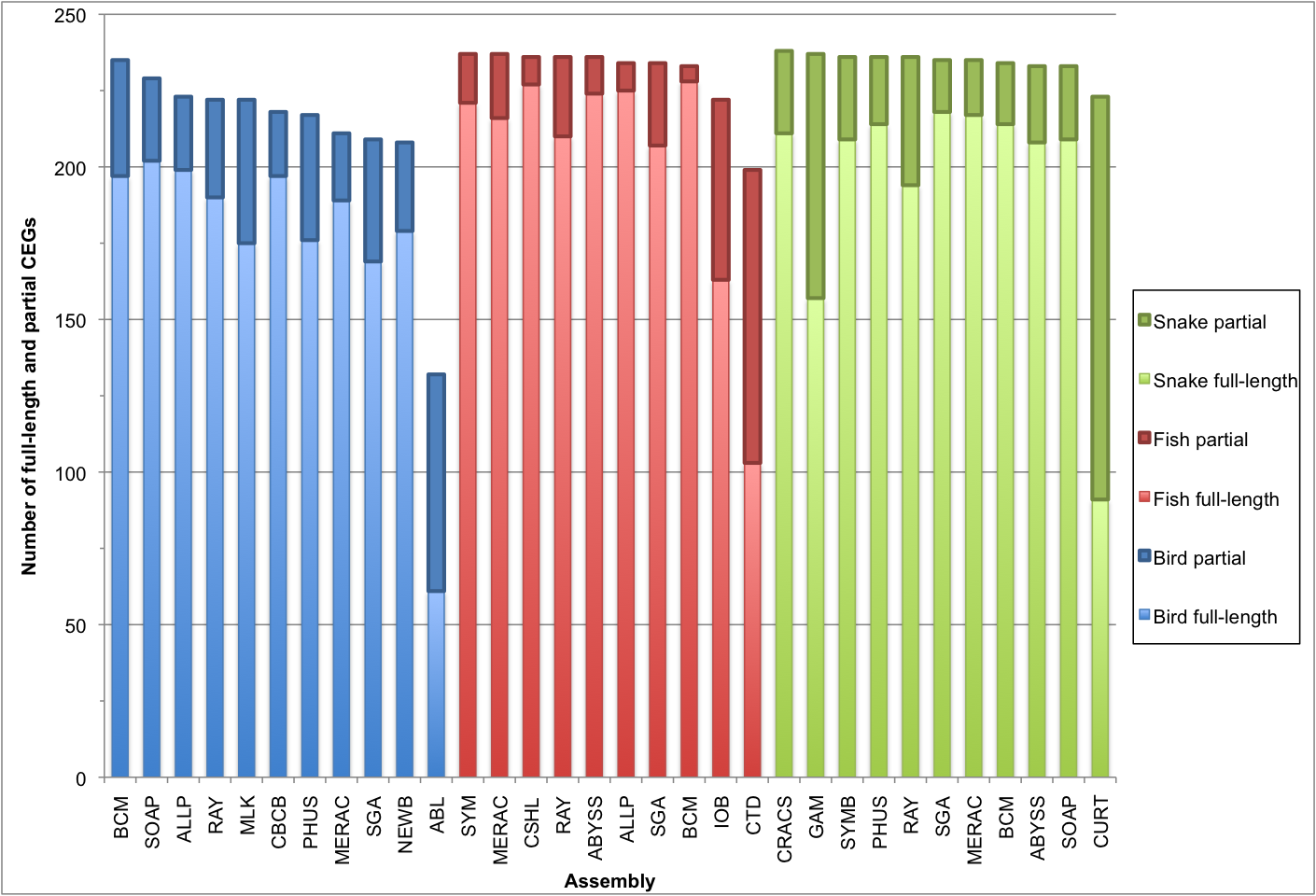


**Figure S8. Relationship between assembly size and Fosmid coverage in bird and snake assemblies.**

Coverage calculated using the COMPASS tool


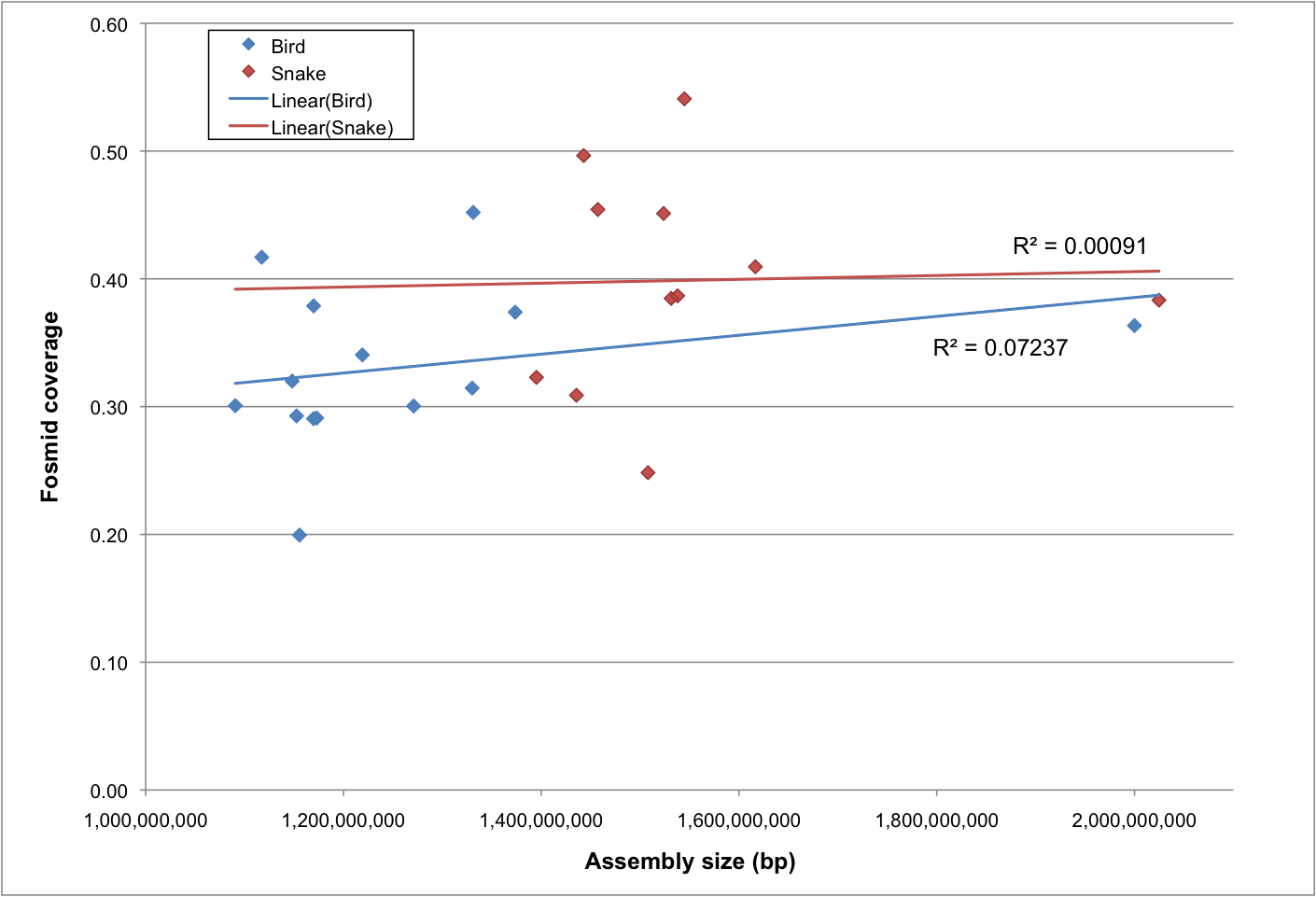


**Figure S9. Average rank of bird assemblies when assessed by ten key metrics.**

Each assembly was ranked by ten different key metrics and then an average rank was calculated. Positive and negative error bars reflect the best and worst average rank that could be achieved if any one key metric was omitted from the analysis. Assemblies in red represent evaluation entries.


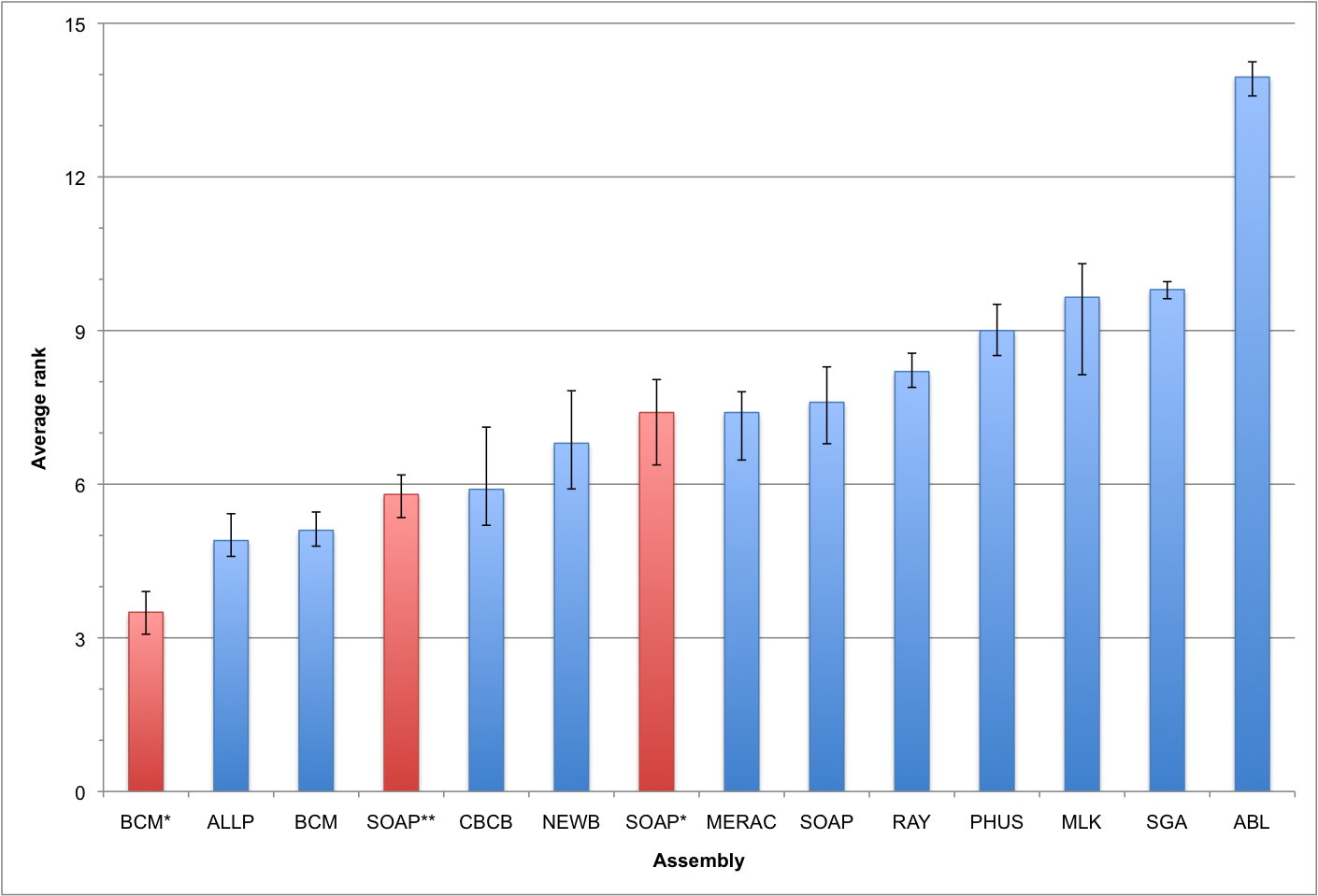


**Figure S10. Average rank of fish assemblies when assessed by seven key metrics.**

Each assembly was ranked by seven different key metrics and then an average rank was calculated. Positive and negative error bars reflect the best and worst average rank that could be achieved if any one key metric was omitted from the analysis. Assemblies in red represent evaluation entries.


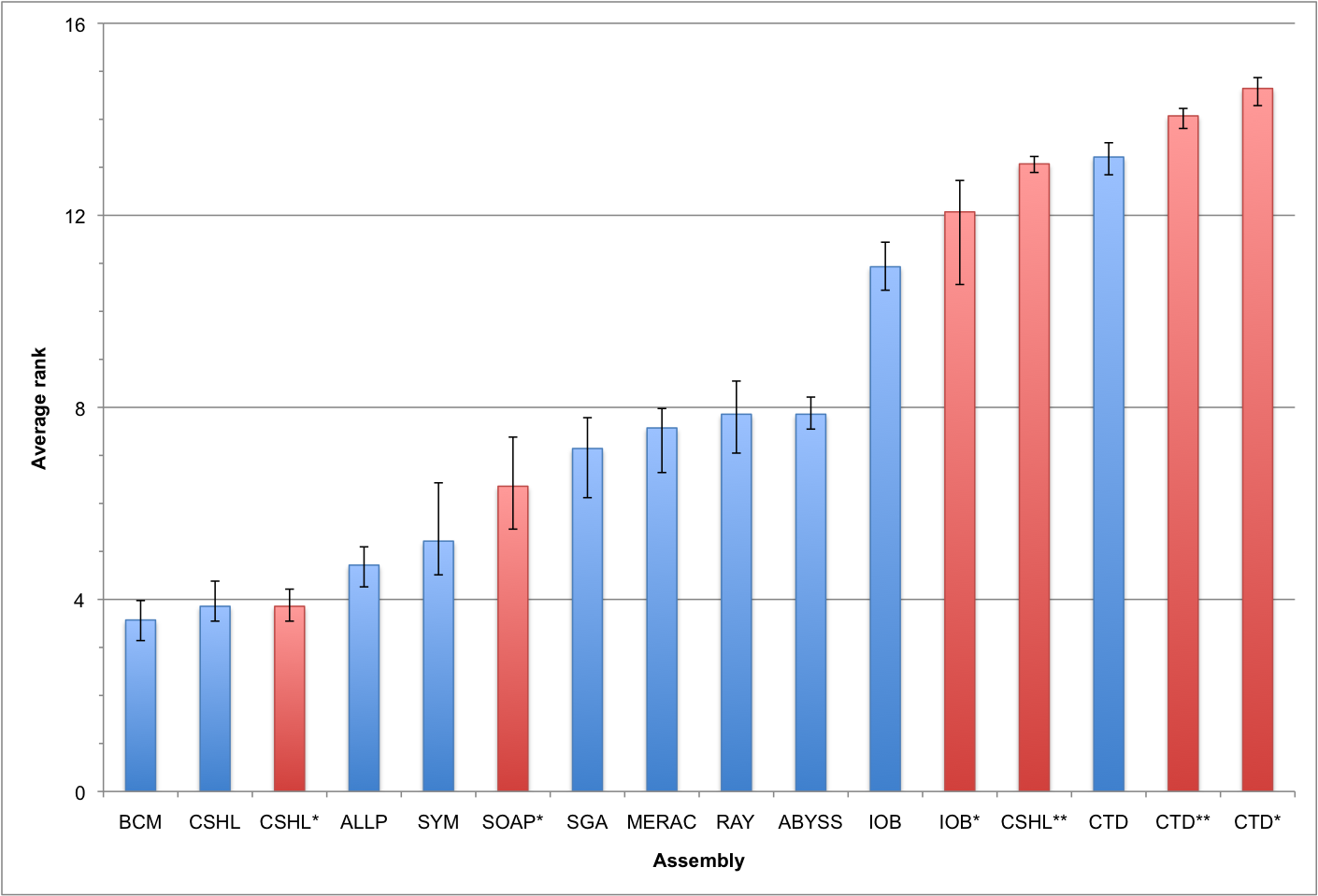


**Figure S11. Average rank of snake assemblies when assessed by ten key metrics.**

Each assembly was ranked by ten different key metrics and then an average rank was calculated. Positive and negative error bars reflect the best and worst average rank that could be achieved if any one key metric was omitted from the analysis.


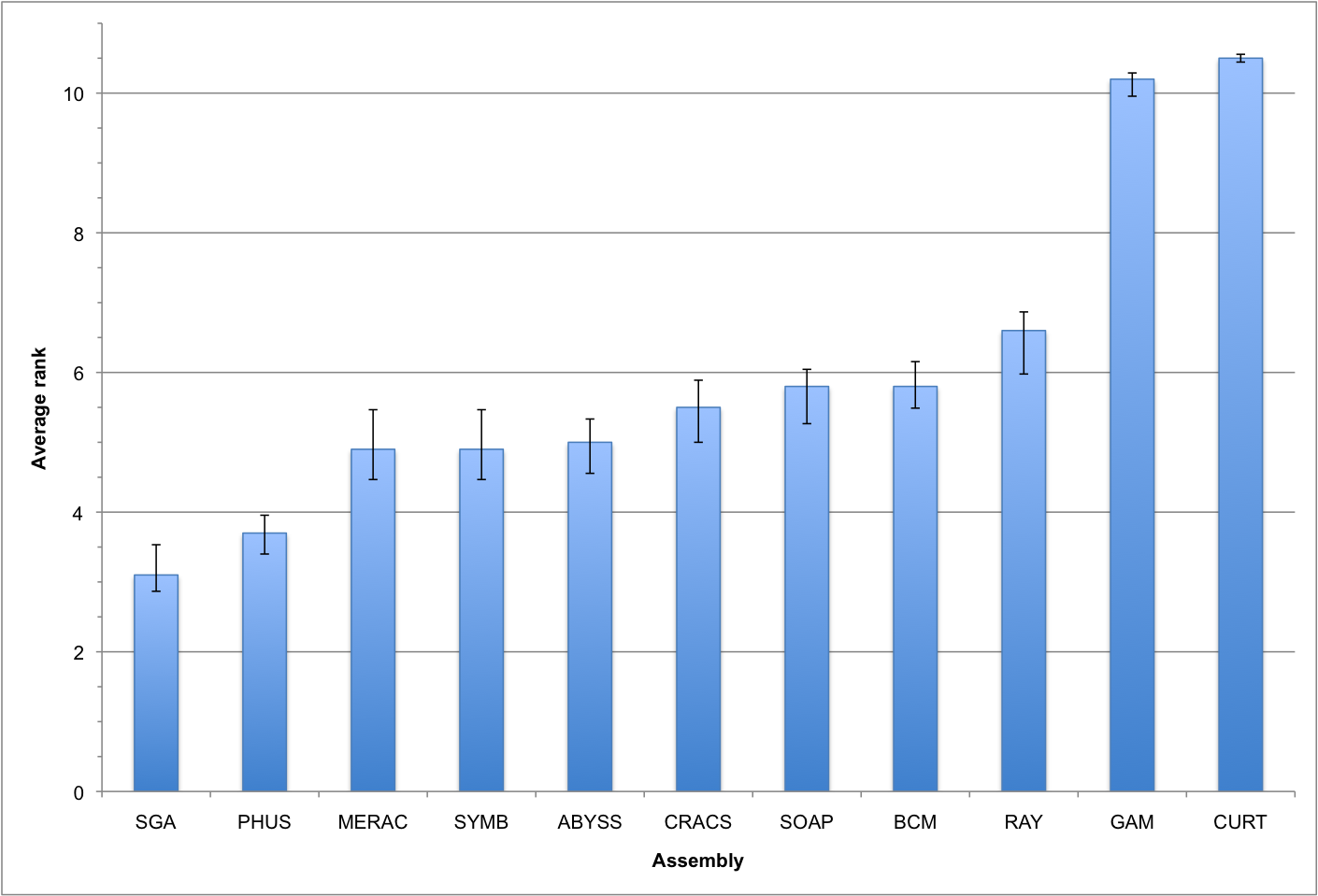


**Figure S12. Correlation between key metrics in bird.**

Pairwise Pearson's correlation matrix. Above the diagonals are Pearson's R correlations with significance (Bonferroni corrected) indicated as: *** *P* < 0.001; ** *P* < 0.01; * *P* < 0.05. Below the diagonal shows the scatterplot of the intersecting row and column key metrics with a simple linear regression drawn in red. Key metrics are CEGMA (number of 458 core eukaryotic genes present); COVERAGE and VALIDITY (of Validated Fosmid Regions, calculated using COMPASS tool); OPTICAL MAP 1 and OPTICAL MAP 1-3 (coverage of optical maps at level 1 or at all levels); VFRT SCORE (summary score of Validated Fosmid Region Tag analysis), GENE-SIZED (the fraction of an assembly’s scaffolds that are 25 Kbp or longer); SCAFFOLD NG50 and CONTIG NG50 (the lengths of the scaffold or contig that takes the sum length of all scaffolds/contigs past 50% of the estimated genome size); REAPR SCORE (summary score of scaffolds from REAPR tool).


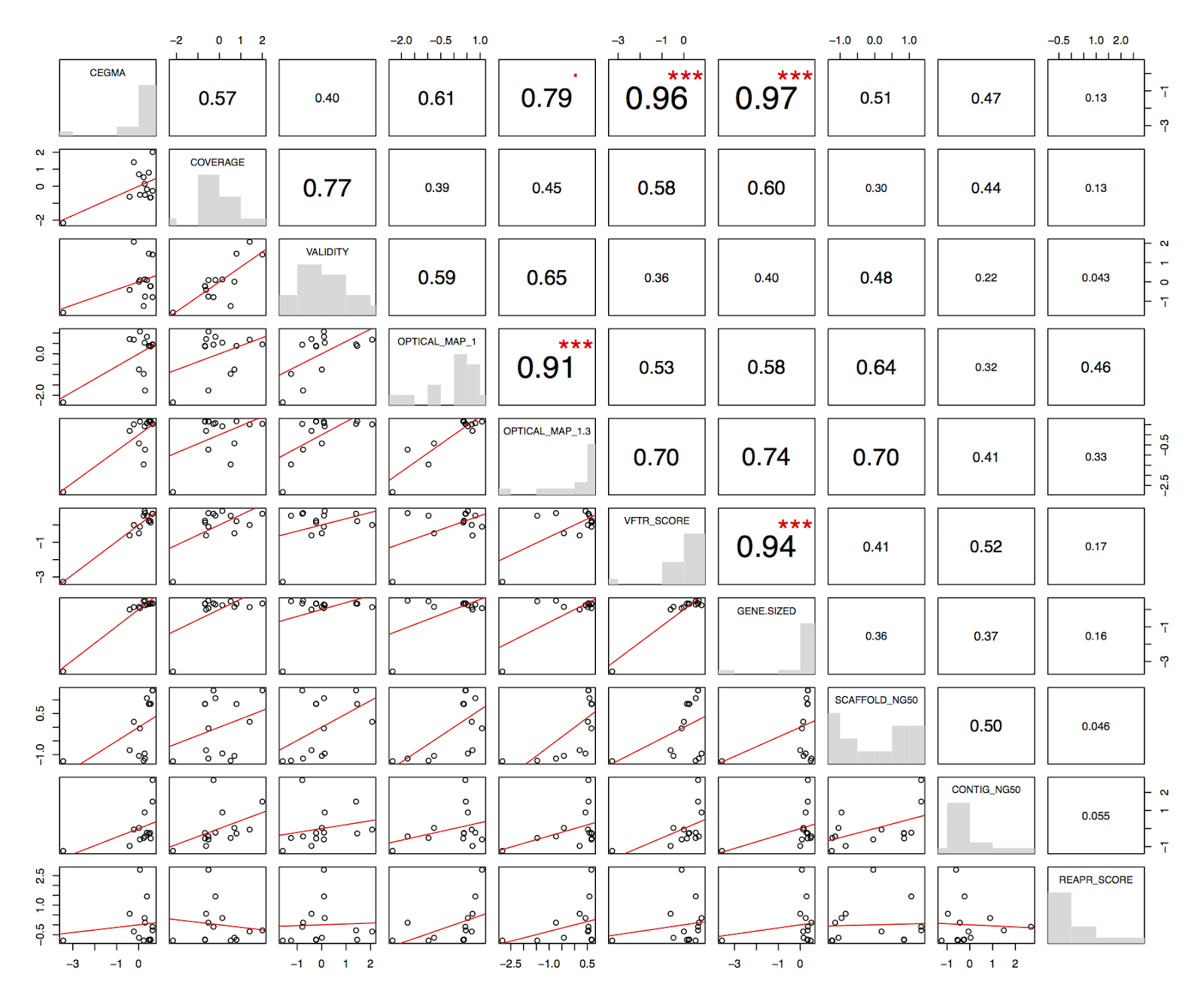


**Figure S13. Correlation between key metrics in fish.**

Pairwise Pearson's correlation matrix. Above the diagonals are Pearson's R correlations with significance (Bonferroni corrected) indicated as: *** *P* < 0.001; ** *P* < 0.01; * *P* < 0.05. Below the diagonal shows the scatterplot of the intersecting row and column key metrics with a simple linear regression drawn in red. Key metrics are CEGMA (number of 458 core eukaryotic genes present); COVERAGE and VALIDITY (of Validated Fosmid Regions, calculated using COMPASS tool); OPTICAL MAP 1 and OPTICAL MAP 1-3 (coverage of optical maps at level 1 or at all levels); VFRT SCORE (summary score of Validated Fosmid Region Tag analysis), GENE-SIZED (the fraction of an assembly’s scaffolds that are 25 Kbp or longer); SCAFFOLD NG50 and CONTIG NG50 (the lengths of the scaffold or contig that takes the sum length of all scaffolds/contigs past 50% of the estimated genome size); REAPR SCORE (summary score of scaffolds from REAPR tool).


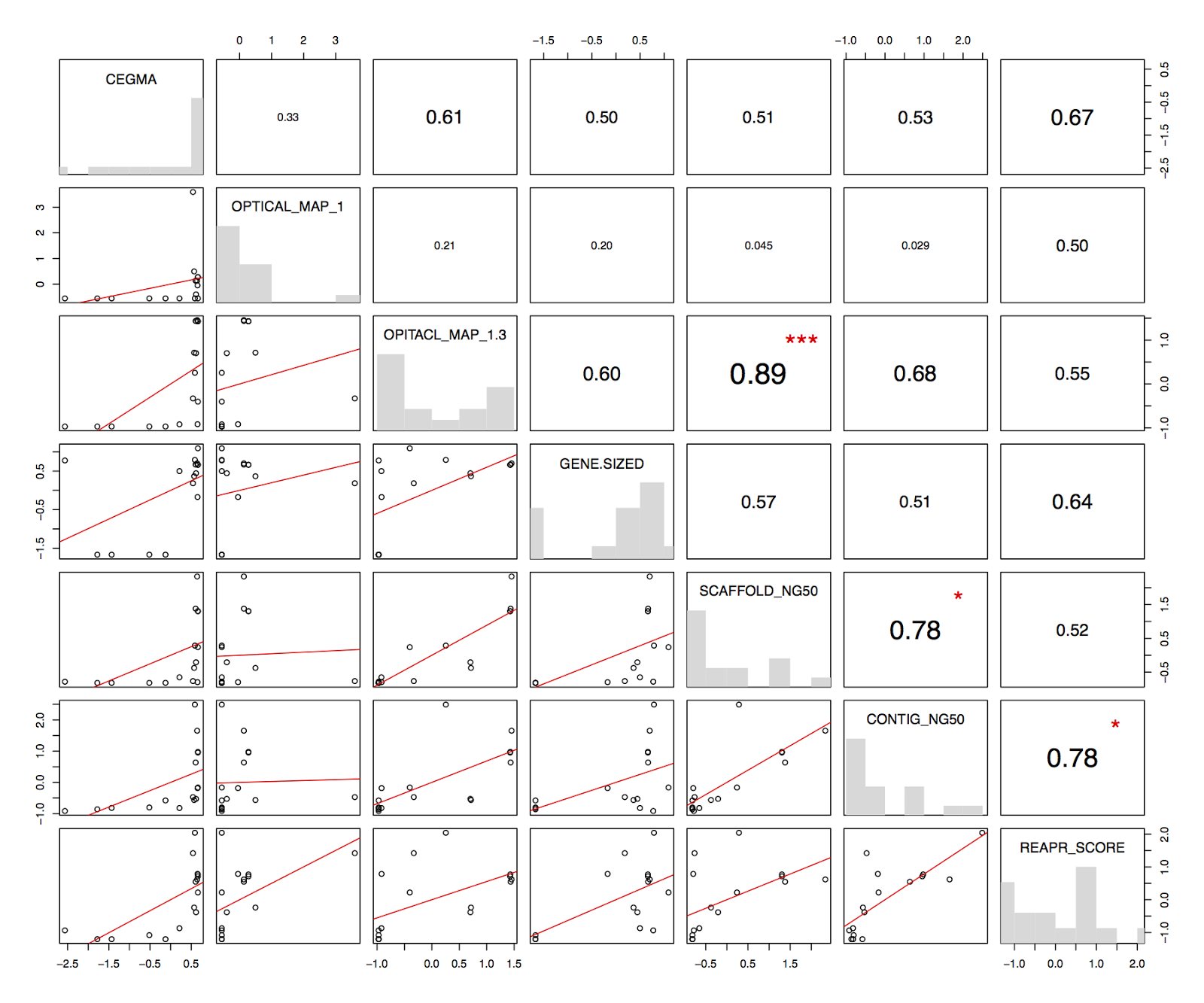


**Figure S14. Correlation between key metrics in snake.**

Pairwise Pearson's correlation matrix. Above the diagonals are Pearson's R correlations with significance (Bonferroni corrected) indicated as: *** *P* < 0.001; ** *P* < 0.01; * *P* < 0.05. Below the diagonal shows the scatterplot of the intersecting row and column key metrics with a simple linear regression drawn in red. Key metrics are CEGMA (number of 458 core eukaryotic genes present); COVERAGE and VALIDITY (of Validated Fosmid Regions, calculated using COMPASS tool); OPTICAL MAP 1 and OPTICAL MAP 1-3 (coverage of optical maps at level 1 or at all levels); VFRT SCORE (summary score of Validated Fosmid Region Tag analysis), GENE-SIZED (the fraction of an assembly’s scaffolds that are 25 Kbp or longer); SCAFFOLD NG50 and CONTIG NG50 (the lengths of the scaffold or contig that takes the sum length of all scaffolds/contigs past 50% of the estimated genome size); REAPR SCORE (summary score of scaffolds from REAPR tool).


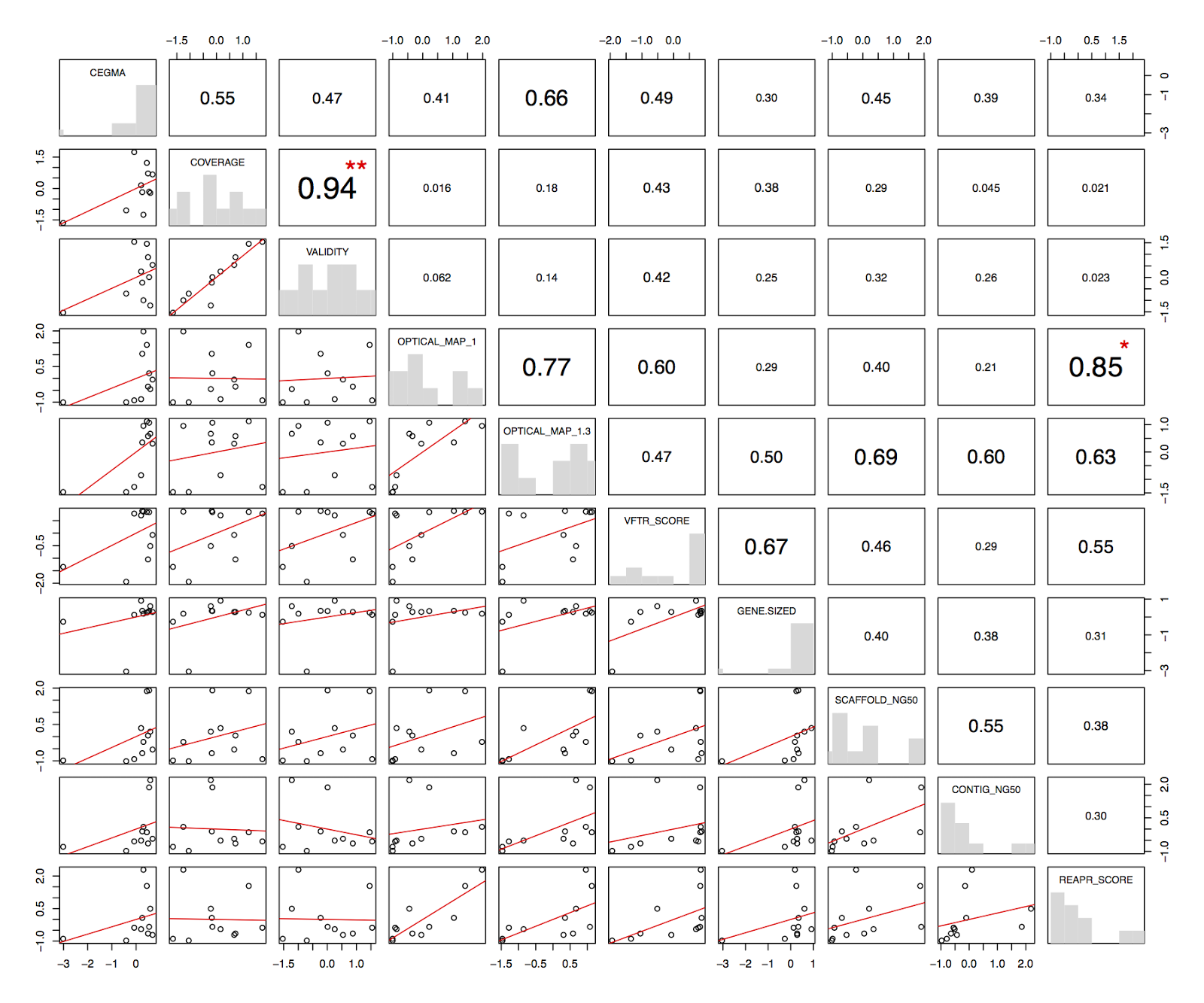


**Figure S15. Correlation between key metrics in bird and snake.**

Pairwise Pearson's correlation matrix from a combined dataset of z-score values from bird and snake. Above the diagonals are Pearson's R correlations with significance (Bonferroni corrected) indicated as: *** *P* < 0.001; ** *P* < 0.01; * *P* < 0.05. Below the diagonal shows the scatterplot of the intersecting row and column key metrics with a simple linear regression drawn in red. Key metrics are CEGMA (number of 458 core eukaryotic genes present); COVERAGE and VALIDITY (of Validated Fosmid Regions, calculated using COMPASS tool); OPTICAL MAP 1 and OPTICAL MAP 1-3 (coverage of optical maps at level 1 or at all levels); VFRT SCORE (summary score of Validated Fosmid Region Tag analysis), GENE-SIZED (the fraction of an assembly’s scaffolds that are 25 Kbp or longer); SCAFFOLD NG50 and CONTIG NG50 (the lengths of the scaffold or contig that takes the sum length of all scaffolds/contigs past 50% of the estimated genome size); REAPR SCORE (summary score of scaffolds from REAPR tool).
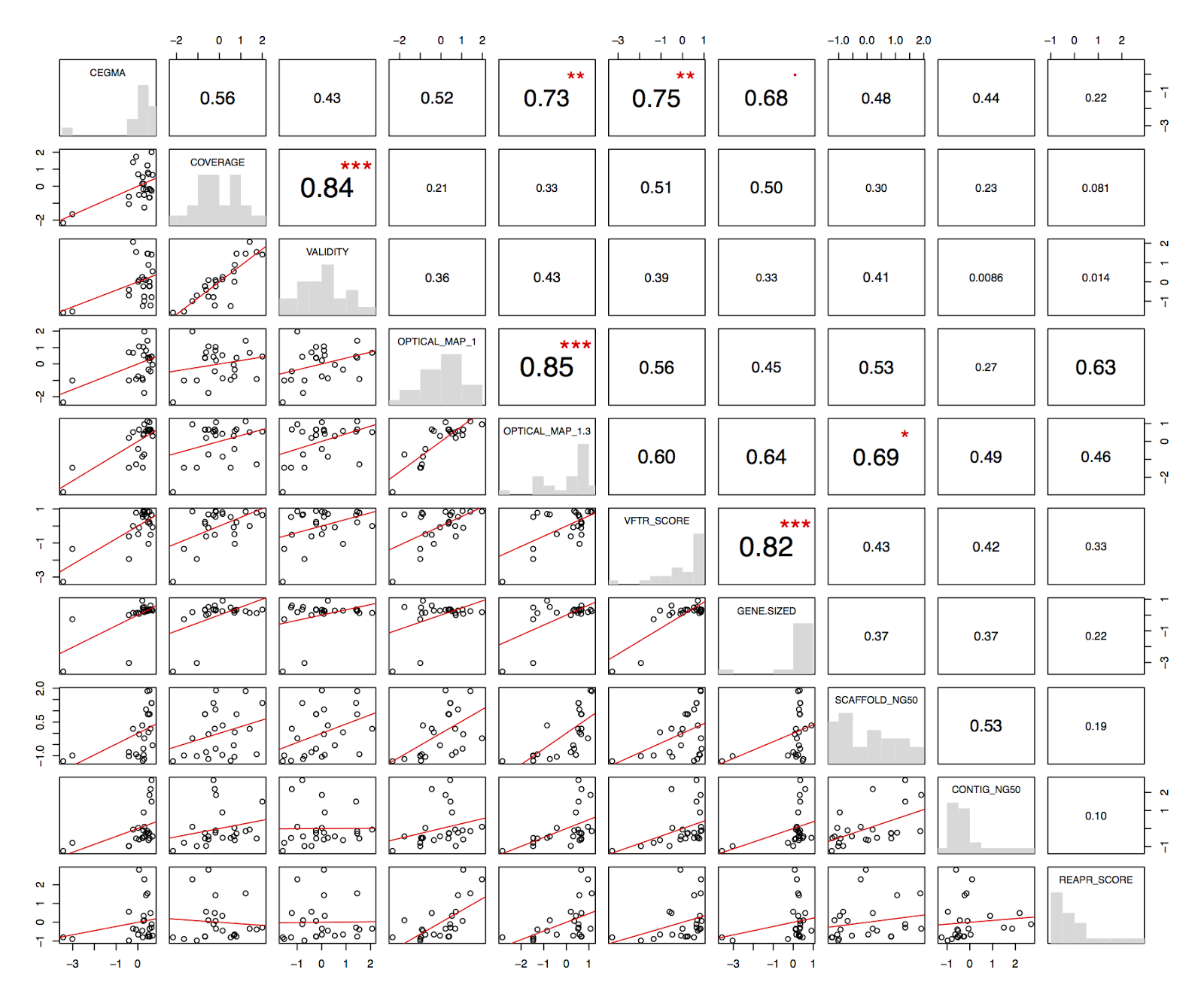


**Figure S16. Heat map showing performance of all assemblies as assessed by z-scores from all key metrics.**

Assemblies arranged in order of their sum z-score (after separating by species). Red and yellow colors indicate higher and lower z-score values respectively. Three key metrics were not computable for fish assemblies and have been left blank. Key metrics are CEGMA (number of 458 core eukaryotic genes present); COVERAGE and VALIDITY (of Validated Fosmid Regions, calculated using COMPASS tool); OPTICAL MAP 1 and OPTICAL MAP 1-3 (coverage of optical maps at level 1 or at all levels); VFRT SCORE (summary score of Validated Fosmid Region Tag analysis), GENE-SIZED (the fraction of an assembly’s scaffolds that are 25 Kbp or longer); SCAFFOLD NG50 and CONTIG NG50 (the lengths of the scaffold or contig that takes the sum length of all scaffolds/contigs past 50% of the estimated genome size); REAPR SCORE (summary score of scaffolds from REAPR tool).


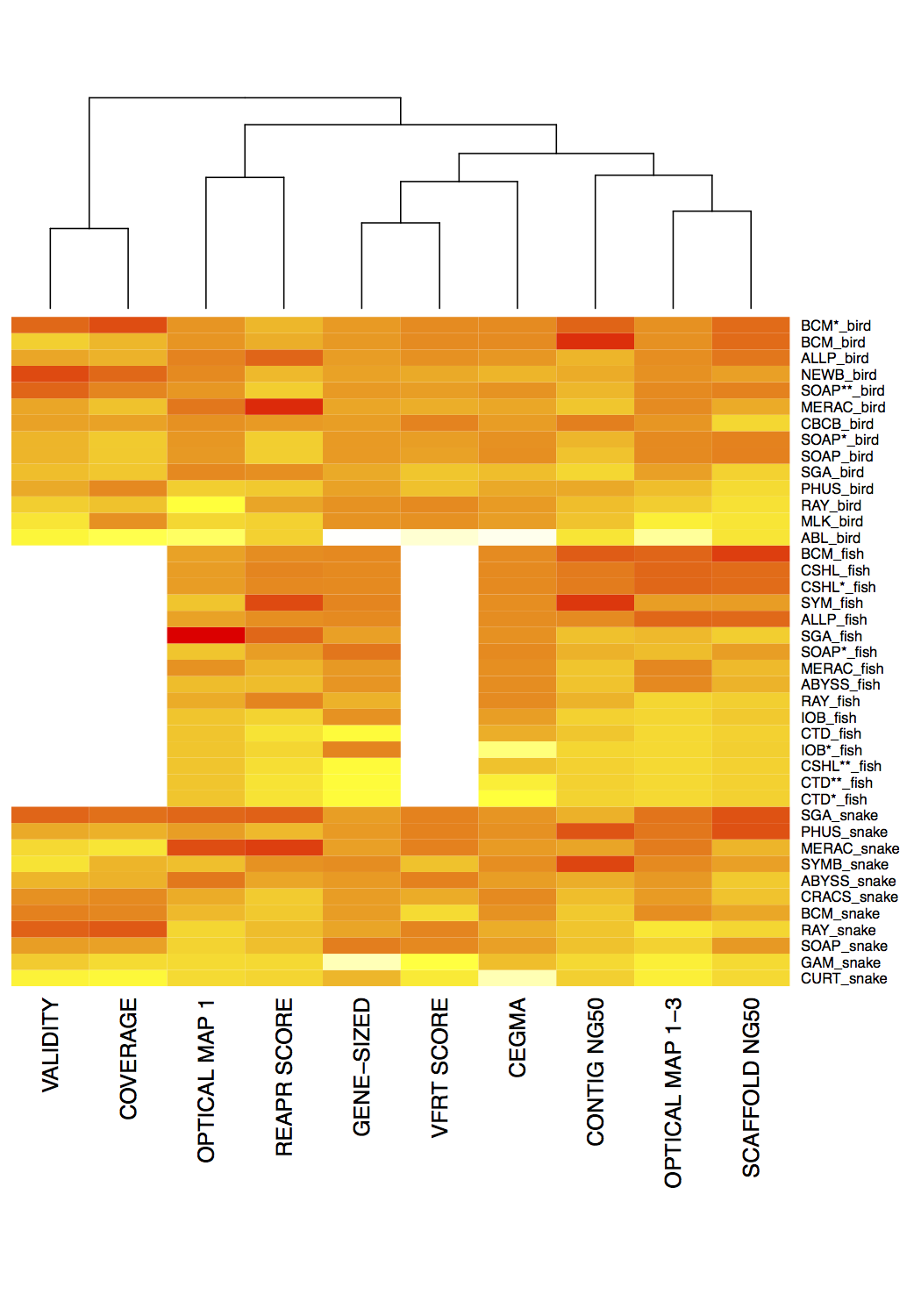


**Figure S17:** **Performance of all snake assemblies when assessed in terms of ‘number of core eukaryotic genes (CEGs) per Mbp of submitted assembly’.**

PRICE assembly (first data point) was excluded from full analysis in the Assemblathon 2 contest because the total assembly size comprised < 25% of the estimated genome size.


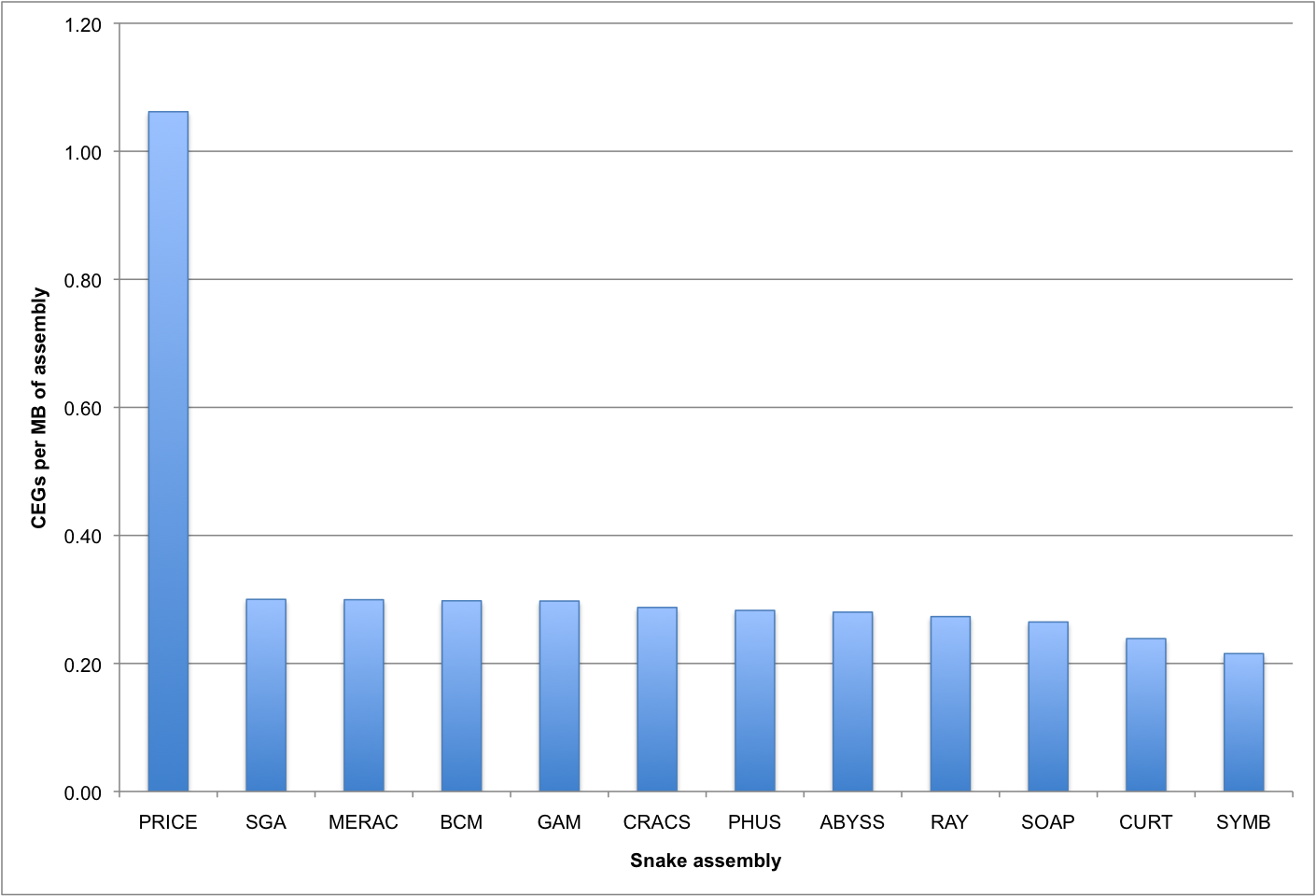


# References

1. Simpson JT, Wong K, Jackman SD, Schein JE, Jones SJM, Birol I: **ABySS: a parallel assembler for short read sequence data.** *Genome Research* 2009**, 19**:1117-1123.

2. Gnerre S, MacCallum I, Przybylski D, Ribeiro FJ, Burton JN, Walker BJ, Sharpe T, Hall G, Shea TP, Sykes S: **High-quality draft assemblies of mammalian genomes from massively parallel sequence data.** *Proceedings of the National Academy of Sciences* 2011**, 108**:1513-1518.

3. Song S, Liu Y, Gibbs RA, Worley KC: **ATLAS-GapFill: A Practical Tool to Improve the Continuity of a Reference Genome Using Second Generation Sequence Data.** (In preparation).

**4**. Deng J, Qu J, Qin X, Gibbs RA, Worley KC: **ATLAS-Link:  Scaffolding Draft Genome Assemblies Using Next-Gen Mate Pair Data.** (In preparation).

**5**. Chaisson MJ, Tesler G: **Mapping single molecule sequencing reads using basic local alignment with successive refinement (BLASR): application and theory.** *BMC Bioinformatics* 2012**, 13**:238.

6. Altschul SF, Gish W, Miller W, Myers EW, Lipman DJ: **Basic local alignment search tool.** *Journal of Molecular Biology* 1990**, 215**:403-410.

7. Langmead B, Trapnell C, Pop M, and Salzberg SL: **Ultrafast and memory-efficient alignment of short DNA sequences to the human genome.** *Genome Biol* 2009**, 10**:R25.

8. Li H, and Durbin R: **Fast and accurate short read alignment with Burrows--Wheeler transform.** *Bioinformatics* 2009**, 25**:1754-1760.

9. Miller JR, Delcher AL, Koren S, Venter E, Walenz BP, Brownley A, Johnson J, Li K, Mobarry C, Sutton G: **Aggressive assembly of pyrosequencing reads with mates.** *Bioinformatics* 2008**, 24**:2818-2824.

10. Casagrande A, Del Fabbro C, Scalabrin S, Policriti A: **GAM: Genomic Assemblies Merger: A Graph Based Method to Integrate Different Assemblies.** In *Bioinformatics and Biomedicine, 2009. BIBM'09. IEEE International Conference on.* 2009:321-326.

11. Li R, Zhu H, Ruan J, Qian W, Fang X, Shi Z, Li Y, Li S, Shan G, Kristiansen K: **De novo assembly of human genomes with massively parallel short read sequencing.** *Genome Research* 2010**, 20**:265-272.

12. Chapman JA, Ho I, Sunkara S, Luo S, Schroth GP, Rokhsar DS: **Meraculous: de novo genome assembly with short paired-end reads.** *PLOS One* 2011**, 6**:e23501.

13. Chikhi R, Lavenier D: **Localized genome assembly from reads to scaffolds: practical traversal of the paired string graph.** *Algorithms in Bioinformatics* 2011:39-48.

14. Quinn NL, Levenkova N, Chow W, Bouffard P, Boroevich KA, Knight JR, Jarvie TP, Lubieniecki KP, Desany BA, Koop BF: **Assessing the feasibility of GS FLX Pyrosequencing for sequencing the Atlantic salmon genome.** *BMC Genomics* 2008**, 9**:404.

15. Koren S, Schatz MC, Walenz BP, Martin J, Howard JT, Ganapathy G, Wang Z, Rasko DA, McCombie WR, Jarvis ED, Phillippy AM: **Hybrid error correction and de novo assembly of single-molecule sequencing reads.** *Nature Biotechnology* 2012**, 30**:693-700.

16. Mullikin JC, Ning Z: **The Phusion Assembler.** *Genome Research* 2003**, 13**:81-90.

17. **PRICE Genome Assembler** [http://derisilab.ucsf.edu/software/price/index.html]

18. Kelley DR, Schatz MC, Salzberg SL: **Quake: quality-aware detection and correction of sequencing errors.** *Genome Biol* 2010**, 11**:R116.19. Boisvert S, Laviolette F, Corbeil J: **Ray: Simultaneous assembly of reads from a mix of high-throughput sequencing technologies.** *Journal of Computational Biology* 2010**, 17**:1519-1533.

20. Li H, Handsaker B, Wysoker A, Fennell T, Ruan J, Homer N, Marth G, Abecasis G, Durbin R: **The sequence alignment/map format and SAMtools.** *Bioinformatics* 2009**, 25**:2078-2079.

21. Simpson JT, Durbin R: **Efficient de novo assembly of large genomes using compressed data structures.** *Genome Research* 2011.

22. Boetzer M, Henkel CV, Jansen HJ, Butler D, Pirovano W: **Scaffolding pre-assembled contigs using SSPACE.** *Bioinformatics* 2011**, 27**:578-579.

23. Zerbino DR, Birney E: **Velvet: algorithms for de novo short read assembly using de Bruijn graphs.** *Genome Research* 2008**, 18**:821-829.

24. Parra G, Bradnam K, Ning Z, Keane T, Korf I: **Assessing the gene space in draft genomes.** *Nucleic Acids Research* 2009**, 37**:289-297.
