# Supplementary material for: Assemblathon 2: evaluating de novo methods of genome assembly in three vertebrate species
Source: Gigascience. 2013 Jul 22;2:10. doi: 10.1186/2047-217X-2-10 (PMC3844414; doi:10.1186/2047-217X-2-10)
Supplement: Additional file 3 — Assembly Instructions. Details provided by participating teams on how to use software to recreate their assemblies. All teams were asked to provide this information. [file 2047-217X-2-10-S3.docx]

# Assembly Instructions

Teams were asked to provide detailed instructions for how to create the assemblies used for the Assemblathon 2 competition. The following is a list of all of the instructions we received.

Table of Contents

Assembly Instructions 1

ABySS team - Fish 2

Allpaths team - Fish 6

BCM-HGSC - Bird 8

Assembly Description 8

Computational requirements 8

BCM-HGSC - Fish 9

Assembly Description 9

Computational requirements 9

BCM-HGSC - Snake 10

Assembly Description 10

Computational requirements 10

BCM-HGSC Software References 11

CBCB team 12

GAM team 13

Assembly Description 13

GAM Software References 13

IOBUGA team 14

Assembly Description 14

Ray team 19

SOAPdenovo team 20

Assembly Description 20

## ABySS team - Fish

The fish paired-end and mate-pair data was assembled using ABySS 1.3.0, followed by additional scaffolding using the fosmid data:

abyss-pe name=fish k=56 s=300 n=10 lib='pe180' mp='mp11k mp9k mp7k mp5k mp2500'

abyss-pe name=fish k=56 s=300 n=5 fosmid_n=2 lib=none mp='fosmid'

**ABySS team - Snake**

The snake paired-end and mate-pair data was assembled using ABySS 1.3.0:

abyss-pe name=snake k=80 s=300 n=10 lib='pe400' mp='mp10k mp4k mp2k'

**Allpaths team - Bird**

-------------------------------------------------------------------------------

Instructions for reproducing the ALLPATHS-LG assemblathon entry for M.undulatus.

--------------------------------------------------------------------------------

Files required:

---------------

Files containing BGI Illumina data for 220, 2000, 5000, 10000, 20000, and 40000 insert sizes. See parrot_groups.csv below for filenames.

parrot_libs.csv file

====================

library_name, project_name, organism_name, type, paired, frag_size, frag_stddev, insert_size, insert_stddev, read_orientation, genomic_start, genomic_end

PARprgDAPDCAAPE, Parrot, Melopsittacus undulatus, fragment, 1, 220, 33, , , inward, ,

PARprgDAPDWAAPE, Parrot, Melopsittacus undulatus, jumping (sheared), 1, , , 2000, 200, outward, ,

PARprgDAPDWBAPE, Parrot, Melopsittacus undulatus, jumping (sheared), 1, , , 2000, 200, outward, ,

PARprgDABDLBAPE, Parrot, Melopsittacus undulatus, jumping (sheared), 1, , , 5000, 500, outward, ,

PARprgDABDLAAPE, Parrot, Melopsittacus undulatus, jumping (sheared), 1, , , 5000, 500, outward, ,

PARprgDAADTAAPE, Parrot, Melopsittacus undulatus, jumping (sheared), 1, , , 10000, 1000, outward, ,

PARprgDAPDUAAPEI-12, Parrot, Melopsittacus undulatus, jumping (sheared), 1, , , 20000, 2000, outward, ,

PARprgDABDVAAPEI-6, Parrot, Melopsittacus undulatus, jumping (sheared), 1, , , 40000, 4000, outward, ,

parrot_groups.csv file

======================

file_name, library_name, group_name

110428_I327_FCB00D2ACXX_L2_PARprgDAPDCAAPE_*.fq.gz, PARprgDAPDCAAPE, 110428_I327_FCB00D2ACXX_L2_PARprgDAPDCAAPE

110503_I266_FCB05AKABXX_L5_PARprgDAPDWBAPE_*.fq.gz, PARprgDAPDWBAPE, 110503_I266_FCB05AKABXX_L5_PARprgDAPDWBAPE

110503_I266_FCC00ADABXX_L5_PARprgDAPDWAAPE_*.fq.gz, PARprgDAPDWAAPE, 110503_I266_FCC00ADABXX_L5_PARprgDAPDWAAPE

110514_I247_FC81MVPABXX_L5_PARprgDABDLAAPE_*.fq.gz, PARprgDABDLAAPE, 110514_I247_FC81MVPABXX_L5_PARprgDABDLAAPE

110514_I263_FC81P81ABXX_L5_PARprgDAADTAAPE_*.fq.gz, PARprgDAADTAAPE, 110514_I263_FC81P81ABXX_L5_PARprgDAADTAAPE

110514_I263_FC81PACABXX_L5_PARprgDABDLBAPE_*.fq.gz, PARprgDABDLBAPE, 110514_I263_FC81PACABXX_L5_PARprgDABDLBAPE

110515_I260_FCB0618ABXX_L5_PARprgDAPDWBAPE_*.fq.gz, PARprgDAPDWBAPE, 110515_I260_FCB0618ABXX_L5_PARprgDAPDWBAPE

110531_I232_FCB05V6ABXX_L8_PARprgDAPDUAAPEI-12_*.fq.gz, PARprgDAPDUAAPEI-12, 110531_I232_FCB05V6ABXX_L8_PARprgDAPDUAAPEI-12

110531_I277_FCB06B9ABXX_L7_PARprgDABDVAAPEI-6_*.fq.gz, PARprgDABDVAAPEI-6, 110531_I277_FCB06B9ABXX_L7_PARprgDABDVAAPEI-6

To prepare the data for assembly:

---------------------------------

mkdir -p Assemblathon/M.undulatus/attempt_1

Using revision 37666 (or later)

CacheLibs.pl ACTION=Add CACHE_DIR=Assemblathon/M.undulatus/cache IN_LIBS_CSV=parrot_libs.csv

CacheGroups.pl ACTION=Add CACHE_DIR=Assemblathon/M.undulatus/cache IN_GROUPS_CSV=parrot_groups.csv PHRED_64=1

CacheToReads.pl CACHE_DIR=Assemblathon/M.undulatus/cache OUT_HEAD=Assemblathon/M.undulatus/attempt_1/frag_reads_orig GROUPS="{110428_I327_FCB00D2ACXX_L2_PARprgDAPDCAAPE}"

CacheToReads.pl CACHE_DIR=Assemblathon/M.undulatus/cache OUT_HEAD=Assemblathon/M.undulatus/attempt_1/jump_reads_orig GROUPS="{110503_I266_FCC00ADABXX_L5_PARprgDAPDWAAPE,110503_I266_FCB05AKABXX_L5_PARprgDAPDWBAPE,110514_I247_FC81MVPABXX_L5_PARprgDABDLAAPE,110514_I263_FC81PACABXX_L5_PARprgDABDLBAPE,110515_I260_FCB0618ABXX_L5_PARprgDAPDWBAPE,110514_I263_FC81P81ABXX_L5_PARprgDAADTAAPE}"

CacheToReads.pl CACHE_DIR=Assemblathon/M.undulatus/cache OUT_HEAD=Assemblathon/M.undulatus/attempt_1/long_jump_reads_orig GROUPS="{110531_I232_FCB05V6ABXX_L8_PARprgDAPDUAAPEI-12,110531_I277_FCB06B9ABXX_L7_PARprgDABDVAAPEI-6}"

echo 2 > Assemblathon/M.undulatus/attempt_1/ploidy

To reproduce the Assemblathon 2 assembly:

-----------------------------------------

Using revision 38588

RunAllPathsLG PRE=Assemblathon REFERENCE_NAME=M.undulatus DATA_SUBDIR=attempt_1 RUN=run_1 OVERWRITE=True

Using revision 38737 - restarting pipeline with new module FixLocal.

RunAllPathsLG PRE=Assemblathon REFERENCE_NAME=M.undulatus DATA_SUBDIR=attempt_1 RUN=run_1 TARGETS=standard FORCE_TARGETS_OF="{FixLocal}" DONT_UPDATE_TARGETS_OF="{CleanAssembly}" REMODEL=False

To generate a fresh assembly with latest version of ALLPATHS-LG:

----------------------------------------------------------------

RunAllPathsLG PRE=Assemblathon REFERENCE_NAME=M.undulatus DATA_SUBDIR=attempt_1 RUN=run_1

## Allpaths team - Fish

----------------------------------------------------------------------------

Instructions for reproducing the ALLPATHS-LG assemblathon entry for M.zebra.

----------------------------------------------------------------------------

Files required:

---------------

All files containing Broad Institute Illumina data. See zebra_groups.csv below for filenames.

zebra_libs.csv file

====================

library_name, project_name, organism_name, type, paired, frag_size, frag_stddev, insert_size, insert_stddev, read_orientation, genomic_start, genomic_end

Solexa-38739, Zebra, Malawi zebra, fragment, 1, 180, 15, , , inward, ,

Solexa-46074, Zebra, Malawi zebra, jumping (fosill), 1, , , 40000, 4000, inward, 4, 75

Solexa-39450, Zebra, Malawi zebra, jumping (sheared), 1, , , 2500, 250, outward, ,

Solexa-39462, Zebra, Malawi zebra, jumping (sheared), 1, , , 2500, 250, outward, ,

Solexa-51379, Zebra, Malawi zebra, jumping (sheared), 1, , , 11000, 1100, outward, ,

Solexa-50902, Zebra, Malawi zebra, jumping (sheared), 1, , , 9000, 900, outward, ,

Solexa-50914, Zebra, Malawi zebra, jumping (sheared), 1, , , 7000, 700, outward, ,

Solexa-50937, Zebra, Malawi zebra, jumping (sheared), 1, , , 5000, 500, outward, ,

zebra_groups.csv file

======================

file_name, library_name, group_name

625E1AAXX.3.*.fastq, Solexa-38739, 625E1AAXX.3

625E1AAXX.4.*.fastq, Solexa-38739, 625E1AAXX.4

625E1AAXX.2.*.fastq, Solexa-38739, 625E1AAXX.2

625E1AAXX.1.*.fastq, Solexa-38739, 625E1AAXX.1

625E1AAXX.5.*.fastq, Solexa-38739, 625E1AAXX.5

625E1AAXX.6.*.fastq, Solexa-38739, 625E1AAXX.6

625E1AAXX.8.*.fastq, Solexa-38739, 625E1AAXX.8

625E1AAXX.7.*.fastq, Solexa-38739, 625E1AAXX.7

801KYABXX.4.*.fastq, Solexa-39462, 801KYABXX.4

801KYABXX.2.*.fastq, Solexa-39450, 801KYABXX.2

801KYABXX.3.*.fastq, Solexa-39450, 801KYABXX.3

803DNABXX.8.*.fastq, Solexa-51379, 803DNABXX.8

803DNABXX.2.*.fastq, Solexa-50902, 803DNABXX.2

803DNABXX.1.*.fastq, Solexa-50914, 803DNABXX.1

803DNABXX.6.*.fastq, Solexa-50937, 803DNABXX.6

62F6HAAXX.1.*.fastq, Solexa-46074, 62F6HAAXX.1

62F6HAAXX.2.*.fastq, Solexa-46074, 62F6HAAXX.2

To prepare the data for assembly:

---------------------------------

mkdir -p Assemblathon/M.zebra/attempt_1

Using revision 37640 (or later)

CacheLibs.pl ACTION=Add CACHE_DIR=Assemblathon/M.zebra/cache IN_LIBS_CSV=zebra_libs.csv

CacheGroups.pl ACTION=Add CACHE_DIR=Assemblathon/M.zebra/cache IN_GROUPS_CSV=zebra_groups.csv

CacheToReads.pl CACHE_DIR=Assemblathon/M.zebra/cache OUT_HEAD=Assemblathon/M.zebra/attempt_1/frag_reads_orig GROUPS="{625E1AAXX.{1,2,3,4,5,6,7,8}}"

CacheToReads.pl CACHE_DIR=Assemblathon/M.zebra/cache OUT_HEAD=Assemblathon/M.zebra/attempt_1/jump_reads_orig GROUPS="{801KYABXX.4,801KYABXX.2,801KYABXX.3,803DNABXX.8,803DNABXX.2,803DNABXX.1,803DNABXX.6}"

CacheToReads.pl CACHE_DIR=Assemblathon/M.zebra/cache OUT_HEAD=Assemblathon/M.zebra/attempt_1/long_jump_reads_orig GROUPS="{62F6HAAXX.1,62F6HAAXX.2}"

echo 2 > Assemblathon/M.zebra/attempt_1/ploidy

To reproduce the Assemblathon 2 assembly:

-----------------------------------------

Revision 37640 - starting assembly*

RunAllPathsLG PRE=/wga/scr1/ALLPATHS REFERENCE_NAME=M.zebra DATA_SUBDIR=attempt_1 RUN=run_1 OVERWRITE=True TARGETS= TARGETS_RUN="{gap_closed.pathsdb.k96}"

Revision 37658 - continuing using latest code*

RunAllPathsLG PRE=/wga/scr1/ALLPATHS REFERENCE_NAME=M.zebra DATA_SUBDIR=attempt_1 RUN=run_1 OVERWRITE=True TARGETS= TARGETS_RUN="{filled_reads_filt.fastb,extended.unibases.k96.lookup}"

Revision 37743 - continuing using latest code*

RunAllPathsLG PRE=/wga/scr1/ALLPATHS REFERENCE_NAME=M.zebra DATA_SUBDIR=attempt_1 RUN=run_1 OVERWRITE=True

Revision 38732 - restarting pipeline with new module FixLocal

RunAllPathsLG PRE=/wga/scr1/ALLPATHS REFERENCE_NAME=M.zebra DATA_SUBDIR=attempt_1.2 RUN=run_1 OVERWRITE=True

* This assembly was completed prior to the Assemblathon 2 competition using our latest development code, updated twice as the assembly progressed. We then used this assembly as the basis of our Assemblathon entry to save time, just running those modules that had significantly changed.

To generate a fresh assembly with latest version of ALLPATHS-LG:

----------------------------------------------------------------

RunAllPathsLG PRE=Assemblathon REFERENCE_NAME=M.zebra DATA_SUBDIR=attempt_1 RUN=run_1

## BCM-HGSC - Bird

### Assembly Description

All Illumina data was preprocessed by adapter trimming using SeqPrep [1] (with default parameters) and error correcting using Quake [2] (using -k 19), except the 150 bp data from 220 bp inserts from BGI, which was merged into fragments using SeqPrep [1] (with default parameters). The merged fragments and GC-rich Illumina data from UK were assembled using the Newbler assembler [3] (with the -large option). Reads that modeled 400 bp 454 fragment reads were synthesized from this assembly and combined with the real 454 data and co-assembled with the Newbler assembler [3] (with the –large option) and scaffolded with the Atlas-Link software [5] (for mate pair data the min_link=4 in the first iteration and min_link = 3 in second; for short insert data the min_link = 5) using the Illumina data mate information from BGI. In parallel, the merged 220 bp insert data and mate pair data from BGI was assembled using ALLPATHS-LG [4] (with K = 96 TARGETS=standard MIN_CONTIG = 300). Three data sets were used to fill the gaps in scaffolds: 1.Illumina data from BGI (except 220 bp insert) were used to fill the gaps within scaffolds using Atlas-GapFill [6]. 2. Gaps within scaffolds were filled by contigs from the ALLPATHS-LG assembly using blast [7] alignment. 3. The PacBio data were used to fill the gaps in scaffolds using blasr [8] and blast [7] alignment. The competition version (2C) contained all three data sets for gap-filling while the evaluation version (3E) did not include the PacBio data. The final assembly combined these refined scaffolds and contigs with additional unincorporated contigs from the ALLPATHS-LG assembly.

### Computational requirements

Estimated max RAM: 400 GB

Estimated running time: 3.5 weeks

Using a single node with 1 TB RAM and 32 CPUs, as well as a cluster of 100 cores each with 16 GB RAM. The gap filling step used a cluster of 600 cores, each with 16 GB RAM and required a run time of 90 h.

## BCM-HGSC - Fish

### Assembly Description

Illumina data was preprocessed with adapter trimming using SeqPrep [1], and assembled with ALLPATHS-LG [4] (MIN_CONTIG = 500) and scaffolded with the Atlas-Link software [5] (for mate pair data the min_link = 4 in the first iteration and min_link = 3 in second). In parallel, the short insert Illumina data was merged into overlapping fragments using SeqPrep [1] (with default parameters), errors corrected using Quake [2] (using -k 18) and assembled with the Newbler assembler [3] (with the –large option). Gaps in scaffolds from the Atlas-Link step were first filled by illumina data using Atlas-Gapfill [6] and then filled with contigs from the Newbler assembly using blast alignment [7]. The final assembly combined these refined scaffolds and contigs with additional unincorporated contigs from the Newbler assembly.

### Computational requirements

Estimated max RAM: 500 GB

Estimated running time: 2.5 weeks

Using a single node with 1 TB RAM and 32 CPUs, as well as a cluster of 100 cores each with 16 GB RAM. The gap filling step used a cluster of 100 cores, each with 16 GB RAM and required a run time of 60 h.

## BCM-HGSC - Snake

### Assembly Description

Short insert data was preprocessed with adapter trimming using SeqPrep [1] (with default parameters), errors corrected using Quake [2] (using -k 19) and assembled initially with the Newbler assembler [3] (with the -large option). Reads that modeled Illumina 100 bp data from 180bp fragments were synthesized from this assembly, combined with real illumina mate pair data, and reassembled using ALLPATHS-LG [4] (MIN_CONTIG = 300). The initial Newbler assembly was scaffolded using illumina data with the Atlas-Link software [5] (for mate pair data the min_link = 4 in the first iteration and min_link = 3 in second; for short insert data the min_link = 10). Illumina data were used to fill the gaps in scaffolds using Atlas-GapFill [6]; more gaps within scaffolds were then filled by contigs from the ALLPATHS-LG assembly using blast alignment [7]. The final assembly combined these refined scaffolds and contigs with additional unincorporated scaffolds from the ALLPATHS-LG assembly.

### Computational requirements

Estimated max RAM: 300 GB

Estimated running time: 3 weeks

Using a single node with 1 TB RAM and 32 CPUs, as well as a cluster of 100 cores each with 16 GB RAM. The gap filling step used a cluster of 100 cores, each with 16 GB RAM and required a run time of 60 h.

## BCM-HGSC Software References

(1) SeqPrep (version a1e1d38,<https://github.com/jstjohn/SeqPrep>, John St. John, UCSC)

(2) Quake (version 0.2,<http://www.cbcb.umd.edu/software/quake/>, Kelley DR, Schatz MC, Salzberg SL.

Quake: quality-aware detection and correction of sequencing errors. *Genome Biology* 11:R116 2010 (<http://genomebiology.com/2010/11/11/R116/abstract)>)

(3) Newbler (version 2.3,<http://my454.com/products/analysis-software/index.asp>, Margulies M, Egholm M, et al. Genome sequencing in microfabricated high-density picolitre reactors. *Nature*. 2005 Sep 15;437(7057):376-80. Epub 2005 Jul 31.

(4) ALLPATHS-LG (version allpathslg-37405,<http://www.broadinstitute.org/software/allpaths-lg/blog/>)

Gnerre, S., MacCallum, I. *et al.* High-quality draft assemblies of mammalian genomes from massively parallel sequence data, *PNAS USA* January 2011 vol. 108 no. 4 1513-1518.

(<http://dx.doi.org/10.1073/pnas.1017351108>) .

(5) Altas-link (<http://www.hgsc.bcm.tmc.edu/content/Atlas-Link>)

(6) Altas-GapFill (<http://www.hgsc.bcm.tmc.edu/content/atlas-gapfill>)

(7) blast (<http://www.blastalgorithm.com/>)

(8) blasr(http://www.pacificbiosciences.com/products/software/algorithms/)

## CBCB team

The following text provides information on how the Assemblathon 2 parrot hybrid assembly (combining 454 + PacBio + Illumina sequences) was generated.

The source code and pre-compiled binaries for Linux 64bit machines are available at:

http://www.cbcb.umd.edu/software/PBcR/asms/wgs-correction.tar.gz

http://www.cbcb.umd.edu/software/PBcR/asms/wgs-assembly.tar.gz

For the most updated version of CA and PBcR, please see the project wiki page:

http://sourceforge.net/apps/mediawiki/wgs-assembler/index.php

http://sourceforge.net/apps/mediawiki/wgs-assembler/index.php?title=PacBioToCA

The full set of commands and spec files that were used to generate the assembly is available in the following file:

http://korflab.ucdavis.edu/Datasets/Assemblathon/Assemblathon2/team_CBCB_assembly_instructions.tar.gz

## GAM team

### Assembly Description

Reads were quality trimmed with rNA, now erne-filter [1,2] with default parameters, and successively independently assembled into contigs with two softwares: CLC Genomics Workbench v4.0 [3] with default parameters and ABySS v1.2.7 [4] with default parameters but k=50 and n=10. Both assemblies were scaffolded with SSPACE v1.0 [5] with default parameters but -x 0 -k 3.

Finally, scaffolded assemblies were merged with GAM-NGS [6]. In order to merge them, trimmed reads were aligned back to the two assemblies with rNA, now erne-map [1,2] with default parameters and then merge with GAM-NGS with default parameters but --min-block-size 20 (minimum ten reads per block to try merging between blocks) and CLC assembly elected as master assembly and ABySS assembly relegated to slave assembly. CLC assembly was elected as master assembly as it provided better statistics (number of contigs, average contig length, N50).

### GAM Software References

(1) rNA (http://iga-rna.sourceforge.net/), Vezzi F, Del Fabbro C, Tomescu AI, Policriti A. rNA: a Fast and Accurate Short Reads Numerical Aligner. Bioinformatics. 2012; 28:1

(2) ERNE (http://erne.sourceforge.net/), Prezza N, Del Fabbro C, Vezzi F, De Paoli E, Policriti A. ERNE-BS5: Aligning BS-treated Sequences by Multiple Hits on a 5-letters Alphabet. ACM-BCB 2012

(3) CLC Genomics Workbench (http://www.clcbio.com/), CLC Bio, Aarhus, Denmark

(4) ABySS (http://www.bcgsc.ca/platform/bioinfo/software/abyss/) Simpson JT, Wong K, Jackman SD, Schein JE, Jones SJ, Birol I. ABySS: A parallel assembler for short read sequence data. *Genome Research*. 2009;19:6

(5) SSPACE (http://www.baseclear.com/bioinformatics-tools/) Boetzer M, Henkel CV, Jansen HJ, Butler D, Pirovano W. Scaffolding pre-assembled contigs using SSPACE. *Bioinformatics*. 2011; 27:4

(6) GAM-NGS (https://github.com/vice87/gam-ngs/) Vicedomini R, Vezzi F, Scalabrin S, Arvestad L, Policriti A. GAM-NGS: Genomic Assemblies Merger for Next Generation Sequencing. *BMC Bioinformatics*. 2013 **14**(Suppl 7):S6

## IOBUGA team

Reproduced from <http://dna.publichealth.uga.edu/Assemblathon2Result/IOBUGA_Supplimentary.txt>

### Assembly Description

We used a combination of ALLPATH-LG, SOAPcor, and the scaffolder from SOAPdenovo. We ran these programs from a public computing cluster at UGA. Initially, we wanted to assess how well ALLPATH-LG is able to assemble the genome. However, due to the restriction of our computing cluster’s hard drive size, we were only able to run ALLPATH-LG on the fragment library and performed scaffolding through SOAPdenovo.

The following represents the generated log file recorded during our assessment:

07/21/11

Run fastX -quality 28

Run KmerFreq http://soap.genomics.org.cn/about.html#resource2

nohup /home/bigsa/SOAPcor/correction/KmerFreq -i FASTQ.LIST -o FASTQ.LIST.KmerFreq -q 28 -s 50 -f 1 -n 0 &

Failed!

Reason: The total amount of the fastq files may be too big.

Run Corrector

Did not run

07/22/11

Use Quake http://www.cbcb.umd.edu/software/quake/manual.html

tried q-mer scan. killed the qmerscan after one-day running.

07/23/11

Error correction

Bioinformatics. 2011 Jul 1;27(13):i137-i141.Error correction of high-throughput sequencing datasets with non-uniform coverage.Medvedev P, Scott E, Kakaradov B, Pevzner P. http://www.ncbi.nlm.nih.gov/pubmed/21685062

Kelley DR, Schatz MC, Salzberg SL. Quake: quality-aware detection and correction of sequencing errors. Genome Biology 11:R116 2010.

Use JellyFish http://www.cbcb.umd.edu/software/jellyfish/

http://www.cbcb.umd.edu/software/jellyfish/jellyfish-manual-1.1.pdf

jellyfish count -m 22 -o output -c 6 -s 10000000 -t 60 -C 625E1AAXX.1.1.fastq 625E1AAXX.1.2.fastq

jellyfish merge [ALL OUT FILES]

jellyfish dump mer_counts_merged.jf > mer_counts_merged.jf.dump

Convert into Quake kmer format (an output file that lists "kmer \t count" one per line)

cat mer_counts_merged.jf.dump| awk '{if(NR %2 ==1){sub(/>/,""); printf "%s\t",$1} else {print $1}}'|awk '{print $2"\t"$1}' > mer_counts_merged.jf.dump.QuakeFormat

~/Tools/Quake/bin/cov_model.py --int mer_counts_merged.jf.dump.QuakeFormat

Failed! again! Try the other solutions.

[bigsa@cs-test NewFastq]$ ~/Tools/Quake/bin/cov_model.py --int mer_counts_merged.jf.dump.QuakeFormat

Traceback (most recent call last):

File "/home/bigsa/Tools/Quake/bin/cov_model.py", line 251, in ?

main()

File "/home/bigsa/Tools/Quake/bin/cov_model.py", line 33, in main

print 'Cutoff: %s' % open('cutoff.txt').readline().rstrip()

IOError: [Errno 2] No such file or directory: 'cutoff.txt.

07/25/1

Run KmerFreq http://soap.genomics.org.cn/about.html#resource2

nohup /home/bigsa/Tools/SOAPcor/correction/KmerFreq -i 625E1AAXX.1-8.list -o 625E1AAXX.1-8.out -n 1 -q 5 &

nohup /home/bigsa/Tools/SOAPcor/correction/KmerFreq -i 62F6HAAXX.1-2.list -o 62F6HAAXX.1-2.out -n 1 -q 5 &

nohup /home/bigsa/Tools/SOAPcor/correction/KmerFreq -i 801KYABXX.2-4.list -o 801KYABXX.2-4.out -n 1 -q 5 &

nohup /home/bigsa/Tools/SOAPcor/correction/KmerFreq -i 803DNABXX.2-8.list -o 803DNABXX.2-8.out -n 1 -q 5 &

[15:20] Run Corrector http://soap.genomics.org.cn/about.html#resource2

nohup /home/bigsa/Tools/SOAPcor/correction/Corrector -i 625E1AAXX.1-8.list -r 625E1AAXX.1-8.out.freq -n 1 -t 15 &

nohup /home/bigsa/Tools/SOAPcor/correction/Corrector -i 62F6HAAXX.1-2.list -r 62F6HAAXX.1-2.out.freq -n 1 -t 15 &

nohup /home/bigsa/Tools/SOAPcor/correction/Corrector -i 801KYABXX.2-4.list -r 801KYABXX.2-4.out.freq -n 1 -t 15 &

nohup /home/bigsa/Tools/SOAPcor/correction/Corrector -i 803DNABXX.2-8.list -r 803DNABXX.2-8.out.freq -n 1 -t 15 &

[15:46] Should not use -t 15 because each thread is taking ~56.8G of memory.

[19:19] Each thread is using ~120G of memory.

[21:09] If there is no change in 2 hours, I will kill all process and do -t 4

-rw-rw-r-- 1 bigsa bigsa 538586593 Jul 25 16:02 62F6HAAXX.2.1.trim.new.fastq.corr

-rw-rw-r-- 1 bigsa bigsa 355363620 Jul 25 16:05 62F6HAAXX.2.2.trim.new.fastq.corr

-rw-rw-r-- 1 bigsa bigsa 337803691 Jul 25 16:06 62F6HAAXX.1.2.trim.new.fastq.corr

-rw-rw-r-- 1 bigsa bigsa 519109327 Jul 25 16:16 62F6HAAXX.1.1.trim.new.fastq.corr

-rw------- 1 bigsa bigsa 5282 Jul 25 16:47 nohup.out

[22:15] kill all Corrector threads

[22:20] Run Corrector http://soap.genomics.org.cn/about.html#resource2

nohup /home/bigsa/Tools/SOAPcor/correction/Corrector -i 625E1AAXX.1-8.list -r 625E1AAXX.1-8.out.freq -n 1 -t 1 &

nohup /home/bigsa/Tools/SOAPcor/correction/Corrector -i 62F6HAAXX.1-2.list -r 62F6HAAXX.1-2.out.freq -n 1 -t 1 &

nohup /home/bigsa/Tools/SOAPcor/correction/Corrector -i 801KYABXX.2-4.list -r 801KYABXX.2-4.out.freq -n 1 -t 1 &

nohup /home/bigsa/Tools/SOAPcor/correction/Corrector -i 803DNABXX.2-8.list -r 803DNABXX.2-8.out.freq -n 1 -t 1 &

[23:15] Run Corrector http://soap.genomics.org.cn/about.html#resource2

/home/bigsa/Tools/SOAPcor/correction/Corrector -i 625E1AAXX.1-8.list -r 625E1AAXX.1-8.out.freq -n 1 -t 50

/home/bigsa/Tools/SOAPcor/correction/Corrector -i 62F6HAAXX.1-2.list -r 62F6HAAXX.1-2.out.freq -n 1 -t 50

#nohup /home/bigsa/Tools/SOAPcor/correction/Corrector -i 801KYABXX.2-4.list -r 801KYABXX.2-4.out.freq -n 1 -t 1 &

/home/bigsa/Tools/SOAPcor/correction/Corrector -i 803DNABXX.2-8.list -r 803DNABXX.2-8.out.freq -n 1 -t 50

07/26/11

[08:55] Run Corrector http://soap.genomics.org.cn/about.html#resource2 [Finish 07/29/11 09:39 ]

/home/bigsa/Tools/SOAPcor/correction/Corrector -i 625E1AAXX.1-8.list -r 625E1AAXX.1-8.out.freq -n 1 -t 3

/home/bigsa/Tools/SOAPcor/correction/Corrector -i 62F6HAAXX.1-2.list -r 62F6HAAXX.1-2.out.freq -n 1 -t 3

#nohup /home/bigsa/Tools/SOAPcor/correction/Corrector -i 801KYABXX.2-4.list -r 801KYABXX.2-4.out.freq -n 1 -t 1 &

/home/bigsa/Tools/SOAPcor/correction/Corrector -i 803DNABXX.2-8.list -r 803DNABXX.2-8.out.freq -n 1 -t 3

Reference genomes of Maylandia zebra

http://www.ncbi.nlm.nih.gov/genomes/leuks.cgi

http://www.ncbi.nlm.nih.gov/Taxonomy/Browser/wwwtax.cgi?id=106582

GPID Organism Group Subgroup TaxID Genome Size (Mb) # Chr Status Method Depth Release Date Center/Consortium

60369 Maylandia zebra Animals Fishes 106582 1000 In Progress WGS 200X Broad Institute

29483 Maylandia zebra Mazinzi Reef Animals Fishes 106582 76.9832 Assembly WGS 0.17X 6/21/2008 Cichlid Genome Consortium [more]

Check kmer distribution by reference genome

Using Tallymer to count k-mer http://www.zbh.uni-hamburg.de/forschung/genominformatik/software/tallymer.html

gt suffixerator -dna -pl -tis -suf -lcp -v -db wgs.ABPM.1.fsa_nt -indexname wgs.ABPM.1.fsa_nt.reads

gt tallymer mkindex -mersize 17 -esa wgs.ABPM.1.fsa_nt.reads > KmerFreq_K17

gt tallymer occratio -output nonuniquemulti relative -mersizes 17 20 30 45 50 55 60 100 500 -esa wgs.ABPM.1.fsa_nt.reads > result2

Kmers on Fish_Reference non-uniq counts %

17 8175320 11.00%

20 6200908 8.40%

30 4579386 6.30%

45 3240256 4.60%

50 2930556 4.20%

55 2664137 3.80%

60 2430855 3.50%

100 1276964 2.00%

500 36704 0.10%

30mer 100mer

chr13 7.3% 0.8%

simRoot 7.3% 0.8%

simMrca 1.9% 0.3%

simA 2.0% 0.3%

simA1 2.0% 0.3%

simA2 2.0% 0.3%

Check read quality by reference genome Using BWA

~/Tools/bwa-0.5.9/bwa index -a bwtsw wgs.ABPM.1.fsa_nt

~/Tools/bwa-0.5.9/bwa aln wgs.ABPM.1.fsa_nt ~/Assemblathon2/SeqCleaned/Fish/NewFastq/625E1AAXX.1.1.fastq > 625E1AAXX.1.1.fastq.sai

~/Tools/bwa-0.5.9/bwa aln wgs.ABPM.1.fsa_nt ~/Assemblathon2/SeqCleaned/Fish/NewFastq/625E1AAXX.1.2.fastq > 625E1AAXX.1.2.fastq.sai

~/Tools/bwa-0.5.9/bwa samse wgs.ABPM.1.fsa_nt 625E1AAXX.1.1.fastq.sai ~/Assemblathon2/SeqCleaned/Fish/NewFastq/625E1AAXX.1.1.fastq > 625E1AAXX.1.1.fastq.sam

~/Tools/bwa-0.5.9/bwa samse wgs.ABPM.1.fsa_nt 625E1AAXX.1.2.fastq.sai ~/Assemblathon2/SeqCleaned/Fish/NewFastq/625E1AAXX.1.2.fastq > 625E1AAXX.1.2.fastq.sam

~/Tools/bwa-0.5.9/bwa sampe wgs.ABPM.1.fsa_nt 625E1AAXX.1.1.fastq.sai 625E1AAXX.1.2.fastq.sai ~/Assemblathon2/SeqCleaned/Fish/NewFastq/625E1AAXX.1.1.fastq ~/Assemblathon2/SeqCleaned/Fish/NewFastq/625E1AAXX.1.2.fastq > 625E1AAXX.1.fastq.sam

samtools view -bST wgs.ABPM.1.fsa_nt 625E1AAXX.1.1.fastq.sam -o 625E1AAXX.1.1.fastq.bam

samtools sort -m 5000000000 625E1AAXX.1.1.fastq.bam 625E1AAXX.1.1.fastq.sort

samtools index 625E1AAXX.1.1.fastq.sort.bam

samtools tview 625E1AAXX.1.2.fastq.sort.bam

samtools view -bST wgs.ABPM.1.fsa_nt 625E1AAXX.1.2.fastq.sam -o 625E1AAXX.1.2.fastq.bam

samtools sort -m 5000000000 625E1AAXX.1.2.fastq.bam 625E1AAXX.1.2.fastq.sort

samtools index 625E1AAXX.1.2.fastq.sort.bam

07/29/11

Run merge_pair.pl http://soap.genomics.org.cn/about.html#resource2

nohup /home/bigsa/Tools/SOAPcor/correction/merge_pair.pl 625E1AAXX.1.1.new.fastq.corr 625E1AAXX.1.2.new.fastq.corr 625E1AAXX.1.1-2.new.fastq.corr &

nohup /home/bigsa/Tools/SOAPcor/correction/merge_pair.pl 625E1AAXX.2.1.new.fastq.corr 625E1AAXX.2.2.new.fastq.corr 625E1AAXX.2.1-2.new.fastq.corr &

nohup /home/bigsa/Tools/SOAPcor/correction/merge_pair.pl 625E1AAXX.3.1.new.fastq.corr 625E1AAXX.3.2.new.fastq.corr 625E1AAXX.3.1-2.new.fastq.corr &

.....

Run KmerFreq on 801* samples [End at Jul 30 04:41]

07/30/11

[23:57]Run Correction on 801* samples

08/03/11

Soapdenovo-63mer

nohup SOAPdenovo-63mer all -s ALL_config.file -o K50_d -K 50 -d -p 60 &

Config file at /home/bigsa/Assemblathon2/SeqCleaned/Fish/MatchNewFastq/CorrMergeFile/ALL_config.file

Start Aug 4 07:15 ALL_config.file

End Aug 4 13:06

http://bioinfo.cs.ccu.edu.tw/wiki/doku.php?id=soapdenovo

http://soap.genomics.org.cn/soapdenovo.html

8/04/11

Gapcloser

nohup /home/bigsa/Tools/GapCloser/GapCloser -o K50_d.scafSeq.GapCloser -b ALL_config.file -a K50_d.scafSeq -p 31 -t 60 &

Start Aug 4 18:02

End Aug 5 10:34

080511

SOAPdenovo-63mer+Gapcloser for K63

SOAPdenovo-63mer+Gapcloser for K40

SOAPdenovo-31mer+Gapcloser for K31

Start 080511 14:25

08/18/11

BLASTN 14:30

nohup /home/bigsa/BLAST/blast-2.2.22/bin/blastall -p blastn -e 0.05 -m 8 -d /home/bigsa/Assemblathon2/Fish_Reference/FishScaffoldBLASTdb -i /home/bigsa/Assemblathon2/SeqCleaned/Fish/MatchNewFastq/CorrMergeFile/Kmer31_Result/K31_d.scafSeq.GapCloser -o /home/bigsa/Assemblathon2/SeqCleaned/Fish/MatchNewFastq/CorrMergeFile/Kmer31_Result/K31_d.scafSeq.GapCloser.BlastNScaffold.m8 &

nohup /home/bigsa/BLAST/blast-2.2.22/bin/blastall -p blastn -e 0.05 -m 8 -d /home/bigsa/Assemblathon2/Fish_Reference/FishESTBLASTdb -i /home/bigsa/Assemblathon2/SeqCleaned/Fish/MatchNewFastq/CorrMergeFile/Kmer31_Result/K31_d.scafSeq.GapCloser -o /home/bigsa/Assemblathon2/SeqCleaned/Fish/MatchNewFastq/CorrMergeFile/Kmer31_Result/K31_d.scafSeq.GapCloser.BlastNEST.m8 &

nohup /home/bigsa/BLAST/blast-2.2.22/bin/blastall -p blastn -e 0.05 -m 8 -d /home/bigsa/Assemblathon2/Fish_Reference/FishScaffoldBLASTdb -i /home/bigsa/Assemblathon2/SeqCleaned/Fish/MatchNewFastq/CorrMergeFile/Kmer40_Result/K40_d.scafSeq.GapCloser -o /home/bigsa/Assemblathon2/SeqCleaned/Fish/MatchNewFastq/CorrMergeFile/Kmer40_Result/K40_d.scafSeq.GapCloser.BlastNScaffold.m8 &

nohup /home/bigsa/BLAST/blast-2.2.22/bin/blastall -p blastn -e 0.05 -m 8 -d /home/bigsa/Assemblathon2/Fish_Reference/FishESTBLASTdb -i /home/bigsa/Assemblathon2/SeqCleaned/Fish/MatchNewFastq/CorrMergeFile/Kmer40_Result/K40_d.scafSeq.GapCloser -o /home/bigsa/Assemblathon2/SeqCleaned/Fish/MatchNewFastq/CorrMergeFile/Kmer40_Result/K40_d.scafSeq.GapCloser.BlastNEST.m8 &

nohup /home/bigsa/BLAST/blast-2.2.22/bin/blastall -p blastn -e 0.05 -m 8 -d /home/bigsa/Assemblathon2/Fish_Reference/FishScaffoldBLASTdb -i /home/bigsa/Assemblathon2/SeqCleaned/Fish/MatchNewFastq/CorrMergeFile/Kmer50_Result/K50_d.scafSeq.GapCloser -o /home/bigsa/Assemblathon2/SeqCleaned/Fish/MatchNewFastq/CorrMergeFile/Kmer50_Result/K50_d.scafSeq.GapCloser.BlastNScaffold.m8 &

nohup /home/bigsa/BLAST/blast-2.2.22/bin/blastall -p blastn -e 0.05 -m 8 -d /home/bigsa/Assemblathon2/Fish_Reference/FishESTBLASTdb -i /home/bigsa/Assemblathon2/SeqCleaned/Fish/MatchNewFastq/CorrMergeFile/Kmer50_Result/K50_d.scafSeq.GapCloser -o /home/bigsa/Assemblathon2/SeqCleaned/Fish/MatchNewFastq/CorrMergeFile/Kmer50_Result/K50_d.scafSeq.GapCloser.BlastNEST.m8 &

nohup /home/bigsa/BLAST/blast-2.2.22/bin/blastall -p blastn -e 0.05 -m 8 -d /home/bigsa/Assemblathon2/Fish_Reference/FishScaffoldBLASTdb -i /home/bigsa/Assemblathon2/SeqCleaned/Fish/MatchNewFastq/CorrMergeFile/Kmer63_Result/K63_d.scafSeq.GapCloser -o /home/bigsa/Assemblathon2/SeqCleaned/Fish/MatchNewFastq/CorrMergeFile/Kmer63_Result/K63_d.scafSeq.GapCloser.BlastNScaffold.m8 &

nohup /home/bigsa/BLAST/blast-2.2.22/bin/blastall -p blastn -e 0.05 -m 8 -d /home/bigsa/Assemblathon2/Fish_Reference/FishESTBLASTdb -i /home/bigsa/Assemblathon2/SeqCleaned/Fish/MatchNewFastq/CorrMergeFile/Kmer63_Result/K63_d.scafSeq.GapCloser -o /home/bigsa/Assemblathon2/SeqCleaned/Fish/MatchNewFastq/CorrMergeFile/Kmer63_Result/K63_d.scafSeq.GapCloser.BlastNEST.m8 &

08/19/11

* Currently running ALLPATHLG at /home/bigsa/Fish

* The final output seems to be located at data/run/ASSEMBLIES/<name>

* Note Need to export LD_LIBRARY_PATH=/usr/local/gcc-4.3.3/lib64:$LD_LIBRARY_PATH before * running prepare.sh or assembly.sh

Failed to produce output due to Harddrive capacity.

09/01/11

Reran ALLPATH-LG on fragment libraries

09/15/11

SOAPdenovo-63mer+Gapcloser for K63

SOAPdenovo-63mer+Gapcloser for K40

SOAPdenovo-31mer+Gapcloser for K31

## Ray team

Sun Grid Engine submission scripts for running Ray on all three Assemblathon 2 datasets are available from the following Github repository:

https://github.com/sebhtml/assemblathon-2-ray

The version of Ray used was 1.7 with a few modifications.

## SOAPdenovo team

### Assembly Description

Reads were filtered and corrected at first. Then these filtered and corrected reads were assembled using different parameters according to the characters of different species. As a last step, gaps in scaffolds were filled.

Assembly pipelines, including programs, shell scripts and configuration files, for bird (for competition purpose), snake (for competition) and fish (for evaluation) are available at:

ftp://assemblathon2:[e5scl4flbles@ftp.genomics.org.cn](mailto:e5scl4flbles@ftp.genomics.org.cn)

Each pipeline includes programs, shell scripts and configuration files. Read the README file in each pipeline file and follow the instructions to reproduce the assemblies.

Note: The submitted SOAPdenovo snake assembly was generated at a time when some of the Illumina mate-pair libraries were temporarily mislabelled (details of 4 Kbp and 10 Kbp libraries were mistakenly switched). A new assembly based, using correct insert sizes of 4 Kbp and 10 Kbp libraries was produced and available at:

<ftp://assemblathon2:e5scl4flbles@ftp.genomics.org.cn>/from_BGISZ/20121220_Boa_constrictor

Note that those mate-pair libraries from flowcell 2 were not used in either the original submitted entry or this new assembly. The scaffold N50 and contig N50 of new assembly are 7,144,364 bp and 53,419 bp, about 4-fold and 3-fold longer than that of the submitted entry, which were 1,772,383 bp and 17,869 bp, respectively.
