# Supplementary material for: Assemblathon 2: evaluating de novo methods of genome assembly in three vertebrate species
Source: Gigascience. 2013 Jul 22;2:10. doi: 10.1186/2047-217X-2-10 (PMC3844414; doi:10.1186/2047-217X-2-10)
Supplement: Additional file 7 — Bird scaffolds mapped to bird Fosmids. Results of using BLAST to align 46 assembled Fosmid sequences to bird scaffold sequences. Each figure represents an assembled Fosmid sequence with tracks showing read coverage, presence of repeats, and alignments to each assembly. [file 2047-217X-2-10-S7.pdf]

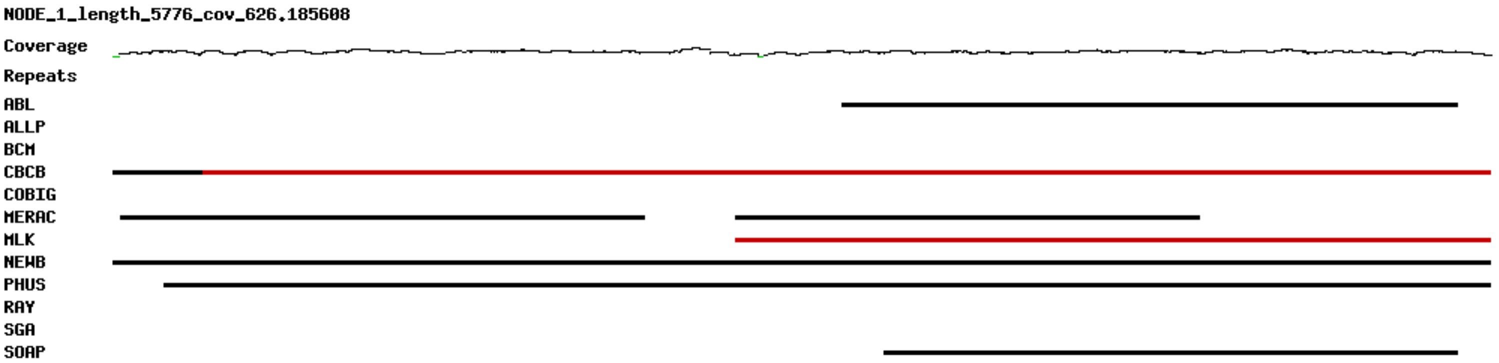
NODE_1_length_5776_cov_626.185608
Coverage
Repeats
ABL
ALLP
BCM
CBCB
COBIG
MERAC
MLK
NEWB
PHUS
RAY
SGA
SOAP

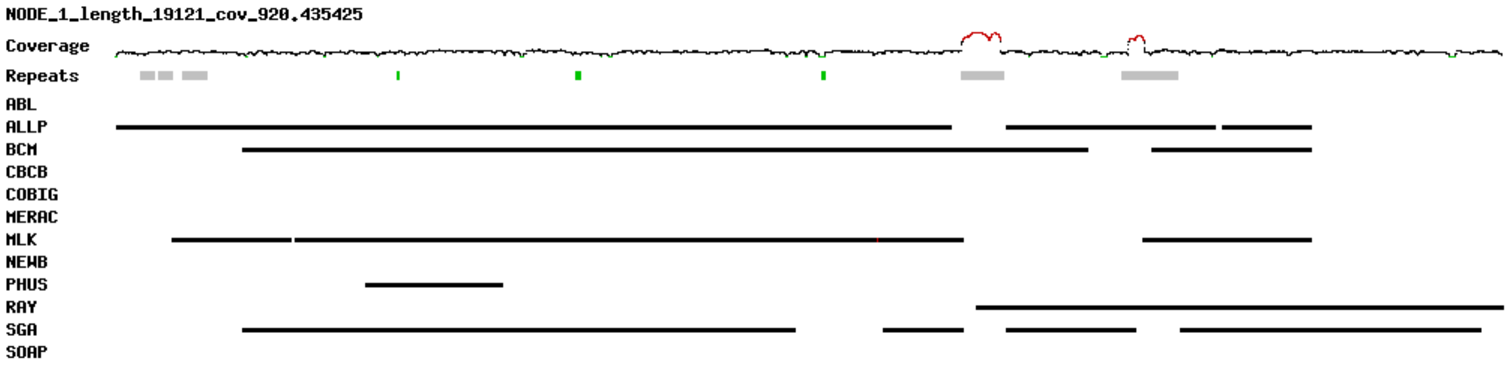
NODE_1_length_19121_cov_920.435425
Coverage
Repeats
ABL
ALLP
BCM
CBCB
COBIG
MERAC
MLK
NEWB
PHUS
RAY
SGA
SOAP

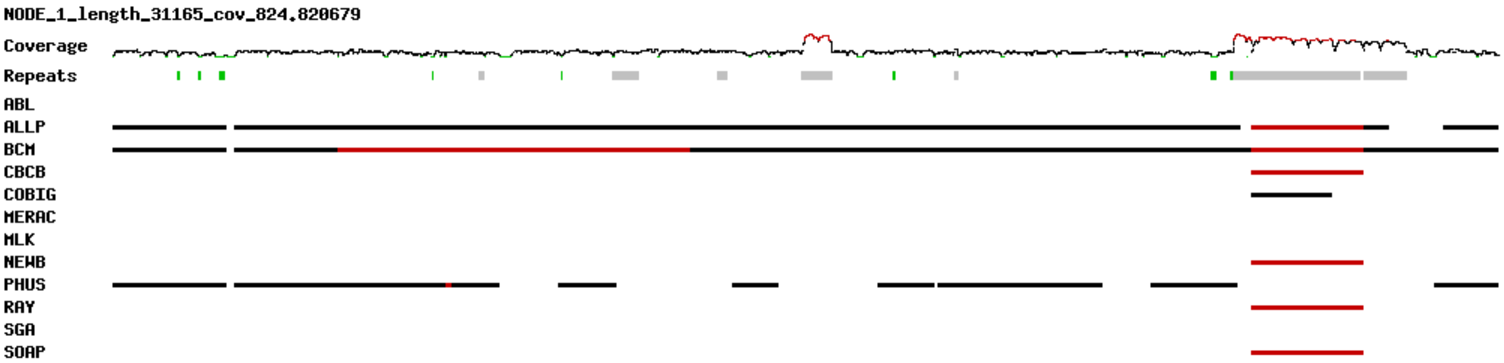
NODE_1_length_31165_cov_824.820679
Coverage
Repeats
ABL
ALLP
BCM
CBCB
COBIG
MERAC
MLK
NEWB
PHUS
RAY
SGA
SOAP

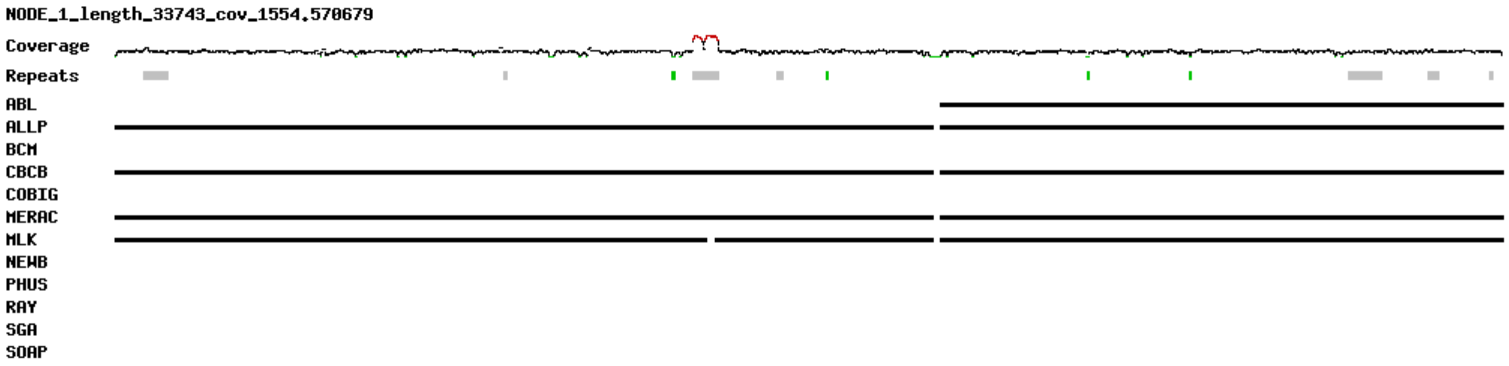
NODE_1_length_33743_cov_1554.570679
Coverage
Repeats
ABL
ALLP
BCM
CBCB
COBIG
MERAC
MLK
NEWB
PHUS
RAY
SGA
SOAP

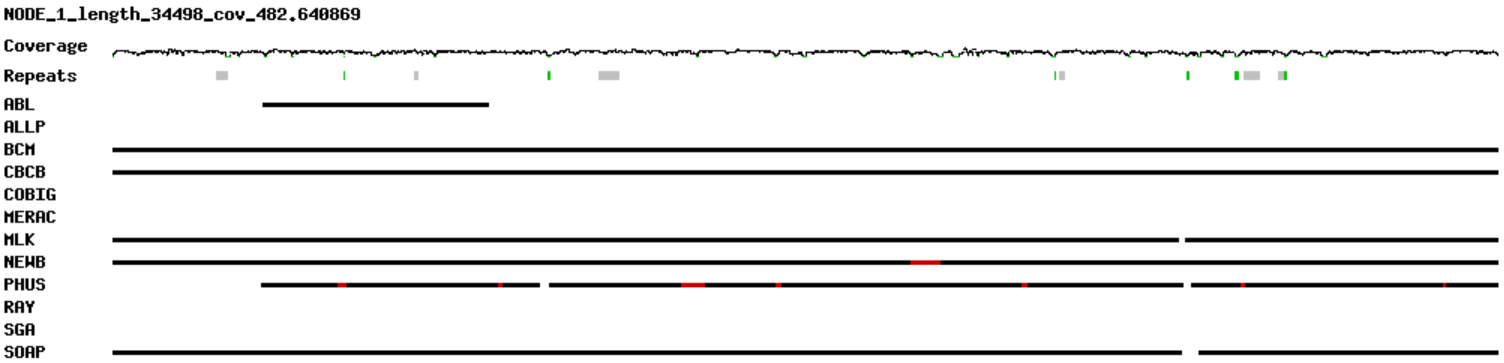
NODE_1_length_34498_cov_482.640869
Coverage
Repeats
ABL
ALLP
BCM
CBCB
COBIG
MERAC
MLK
NEWB
PHUS
RAY
SGA
SOAP

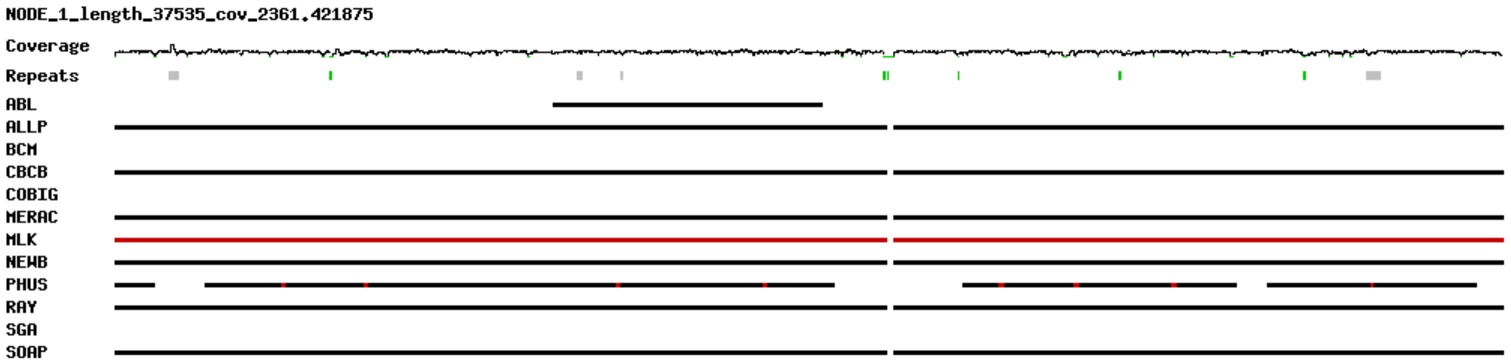
NODE_1_length_37535_cov_2361.421875
Coverage
Repeats
ABL
ALLP
BCM
CBCB
COBIG
MERAC
MLK
NEWB
PHUS
RAY
SGA
SOAP

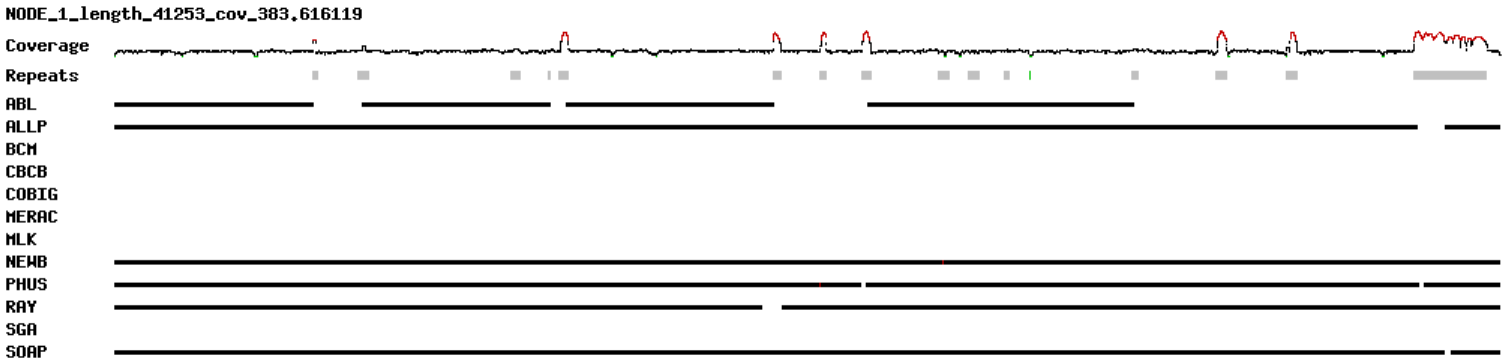

NODE_1_length_41253_cov_383.616119
Coverage
Repeats
ABL
ALLP
BCM
CBCB
COBIG
MERAC
MLK
NEWB
PHUS
RAY
SGA
SOAP

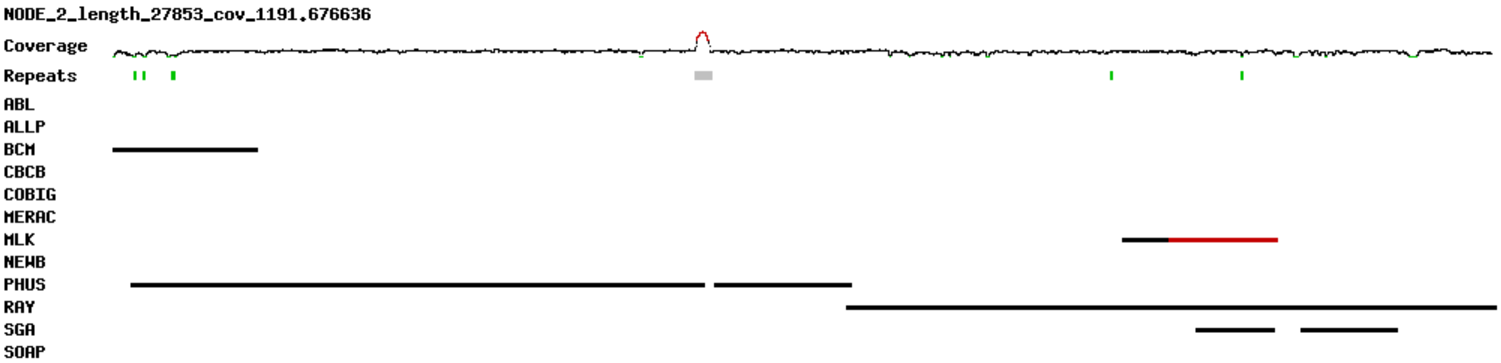
NODE_2_length_27853_cov_1191.676636
Coverage
Repeats
ABL
ALLP
BCM
CBCB
COBIG
MERAC
MLK
NEWB
PHUS
RAY
SGA
SOAP

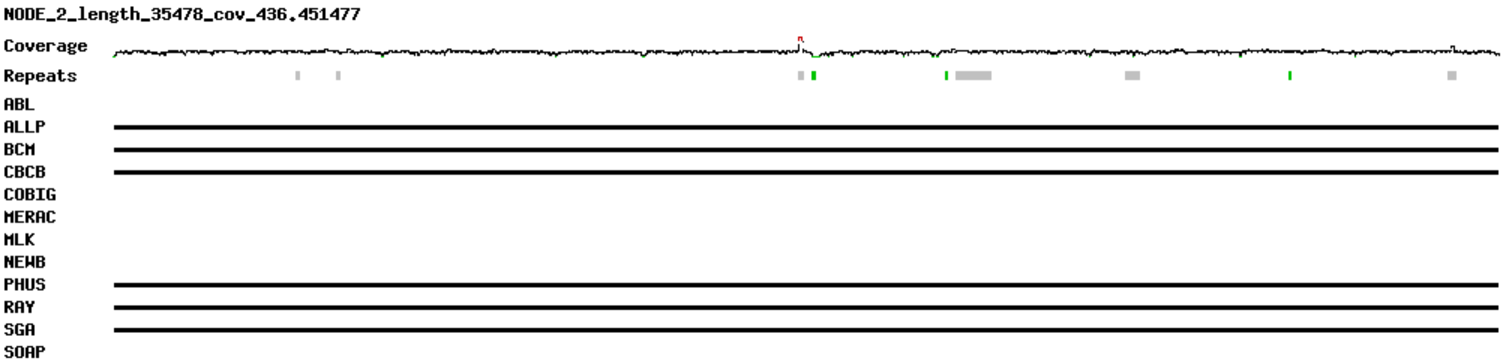
NODE_2_length_35478_cov_436.451477
Coverage
Repeats
ABL
ALLP
BCM
CBCB
COBIG
MERAC
MLK
NEWB
PHUS
RAY
SGA
SOAP

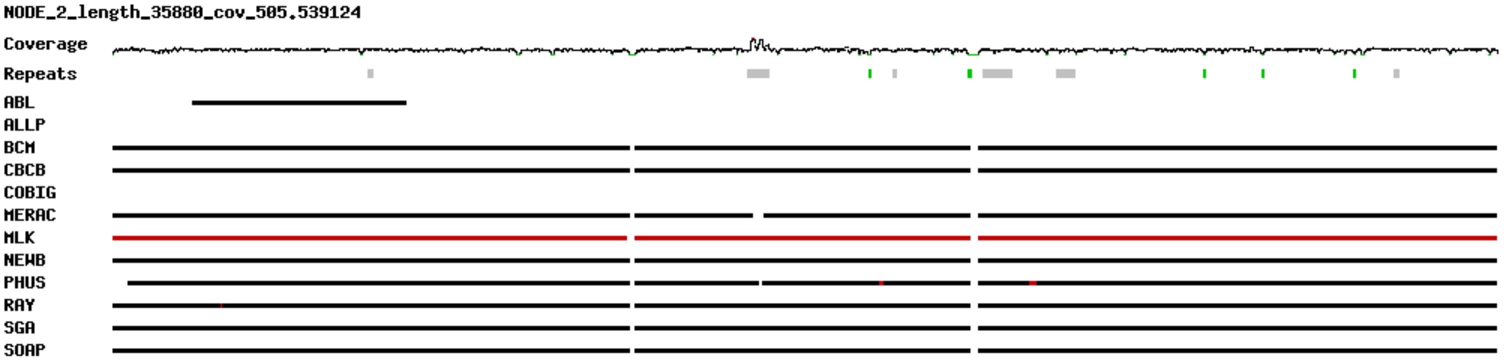
NODE_2_length_35880_cov_505.539124
Coverage
Repeats
ABL
ALLP
BCM
CBCB
COBIG
MERAC
MLK
NEWB
PHUS
RAY
SGA
SOAP

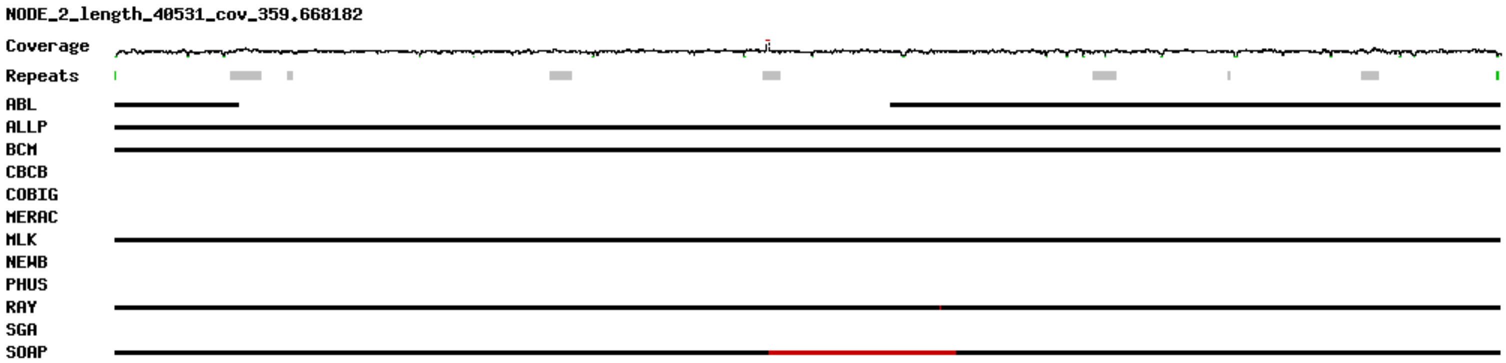
NODE_2_length_40531_cov_359.668182
Coverage
Repeats
ABL
ALLP
BCM
CBCB
COBIG
MERAC
MLK
NEWB
PHUS
RAY
SGA
SOAP

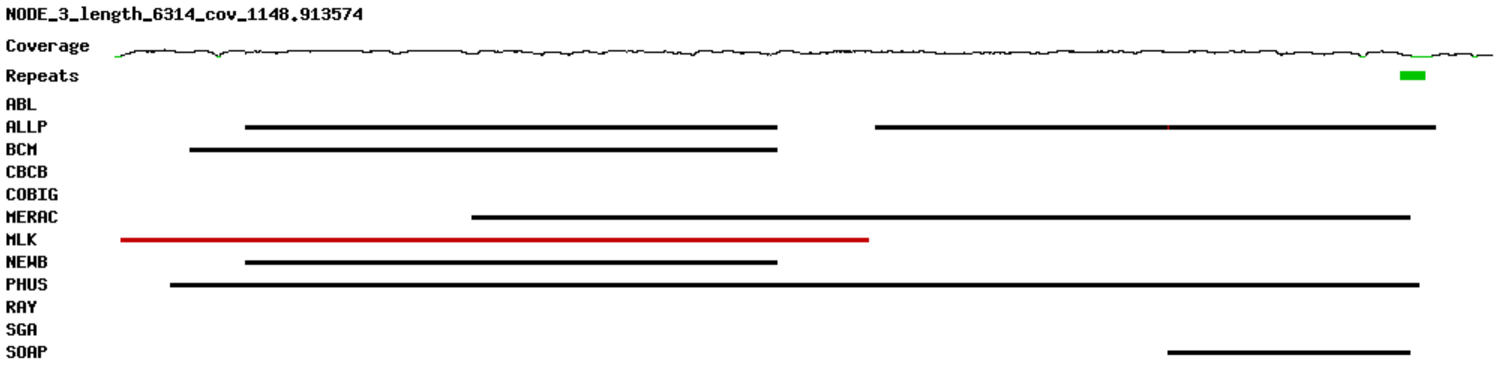
NODE_3_length_6314_cov_1148.913574
Coverage
Repeats
ABL
ALLP
BCM
CBCB
COBIG
MERAC
MLK
NEWB
PHUS
RAY
SGA
SOAP

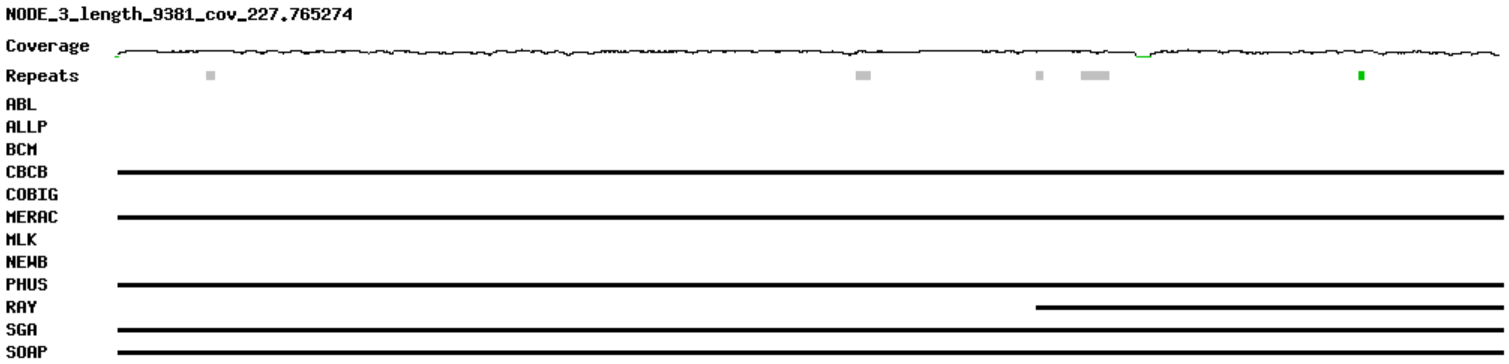
NODE_3_length_9381_cov_227.765274
Coverage
Repeats
ABL
ALLP
BCM
CBCB
COBIG
MERAC
MLK
NEWB
PHUS
RAY
SGA
SOAP

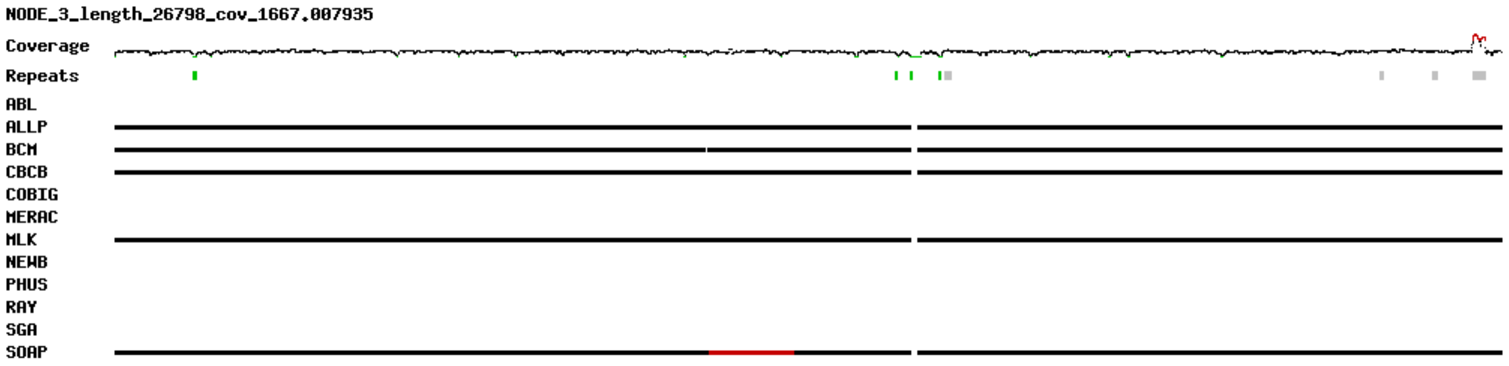
NODE_3_length_26798_cov_1667.007935
Coverage
Repeats
ABL
ALLP
BCM
CBCB
COBIG
MERAC
MLK
NEWB
PHUS
RAY
SGA
SOAP

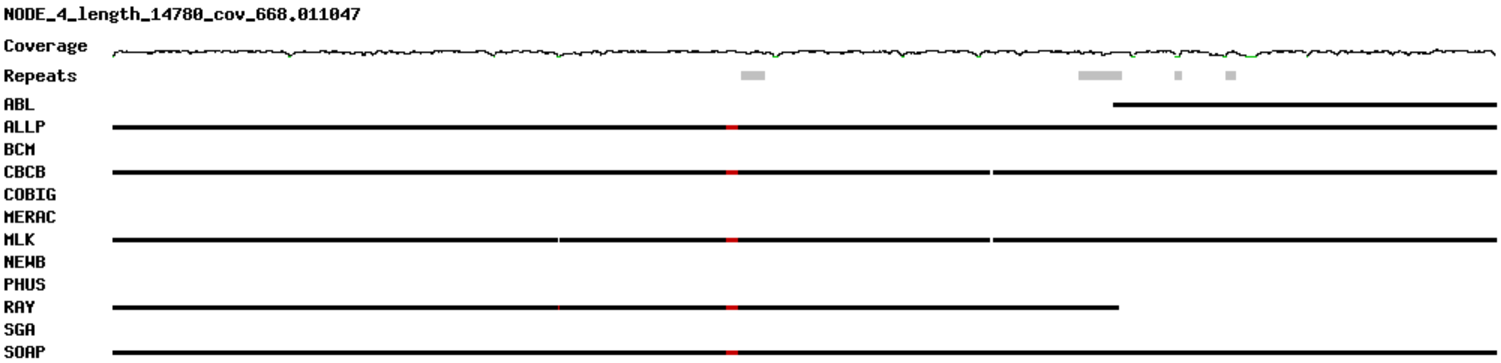
NODE_4_length_14780_cov_668.011047
Coverage
Repeats
ABL
ALLP
BCM
CBCB
COBIG
MERAC
MLK
NEWB
PHUS
RAY
SGA
SOAP

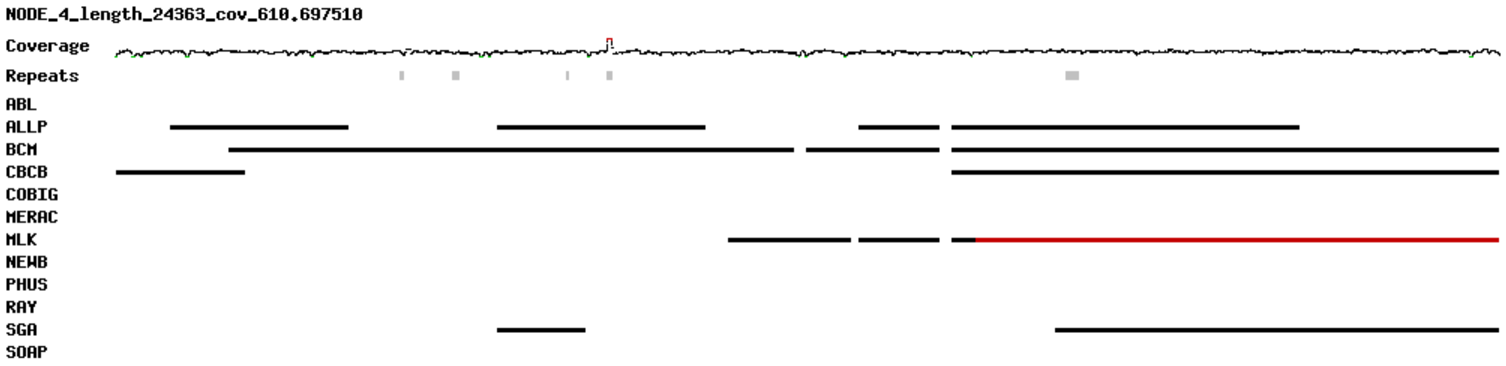
NODE_4_length_24363_cov_610.697510
Coverage
Repeats
ABL
ALLP
BCM
CBCB
COBIG
MERAC
MLK
NEWB
PHUS
RAY
SGA
SOAP

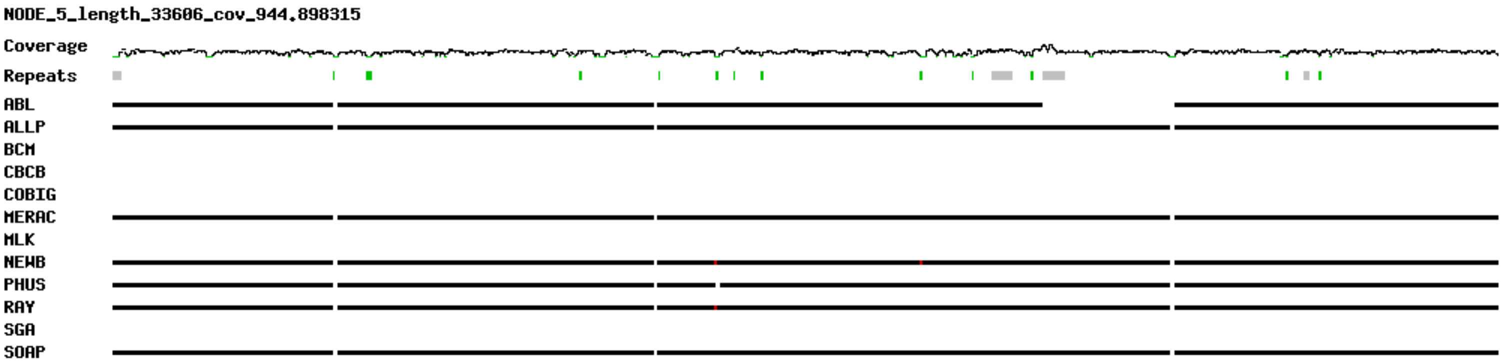
NODE_5_length_33606_cov_944.898315
Coverage
Repeats
ABL
ALLP
BCM
CBCB
COBIG
MERAC
MLK
NEWB
PHUS
RAY
SGA
SOAP

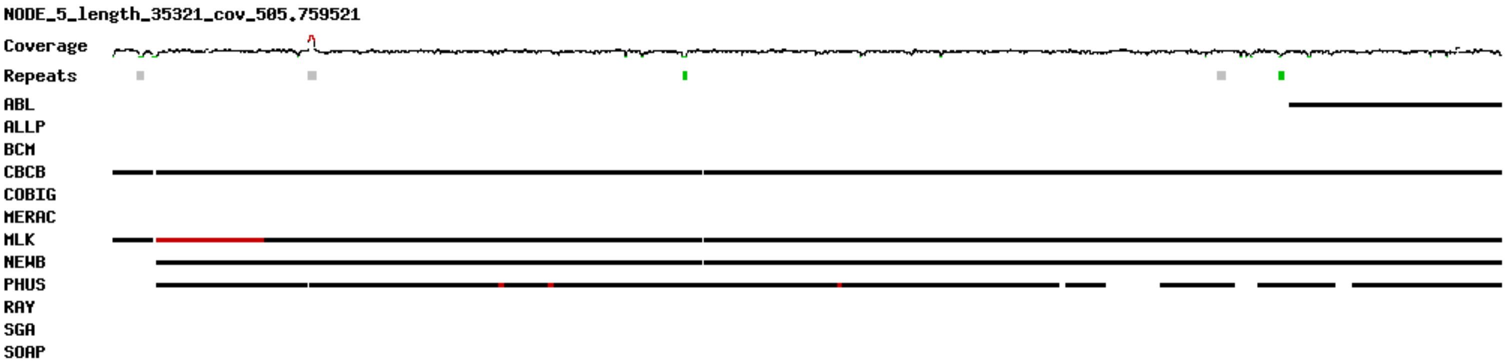
NODE_5_length_35321_cov_505.759521
Coverage
Repeats
ABL
ALLP
BCM
CBCB
COBIG
MERAC
MLK
NEWB
PHUS
RAY
SGA
SOAP

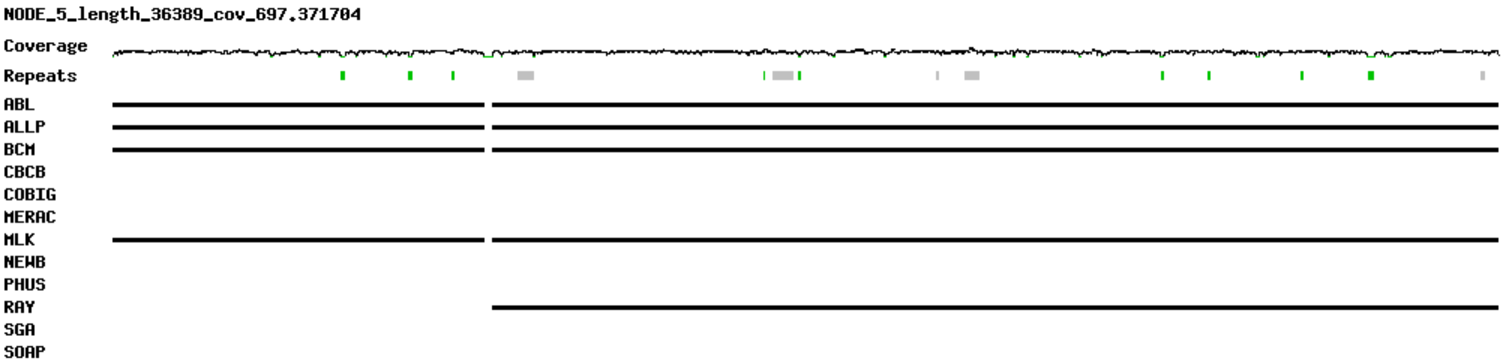
NODE_5_length_36389_cov_697.371704
Coverage
Repeats
ABL
ALLP
BCM
CBCB
COBIG
MERAC
MLK
NEWB
PHUS
RAY
SGA
SOAP

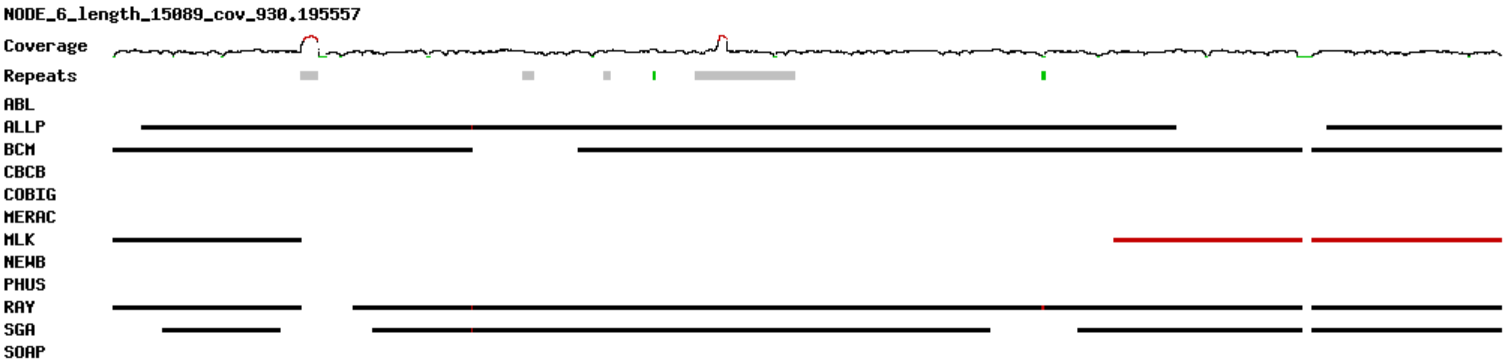
NODE_6_length_15089_cov_930.195557
Coverage
Repeats
ABL
ALLP
BCM
CBCB
COBIG
MERAC
MLK
NEWB
PHUS
RAY
SGA
SOAP

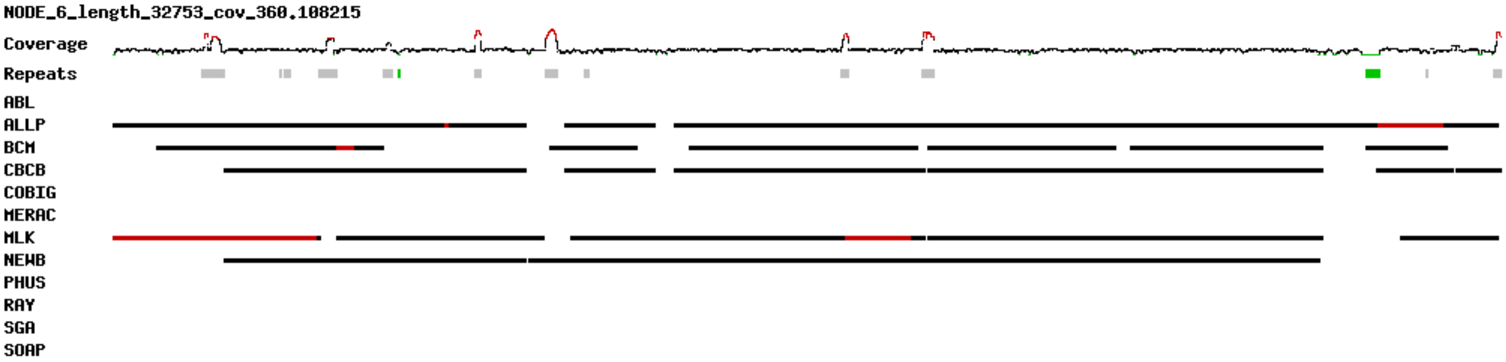
NODE_6_length_32753_cov_360.108215
Coverage
Repeats
ABL
ALLP
BCM
CBCB
COBIG
MERAC
MLK
NEWB
PHUS
RAY
SGA
SOAP

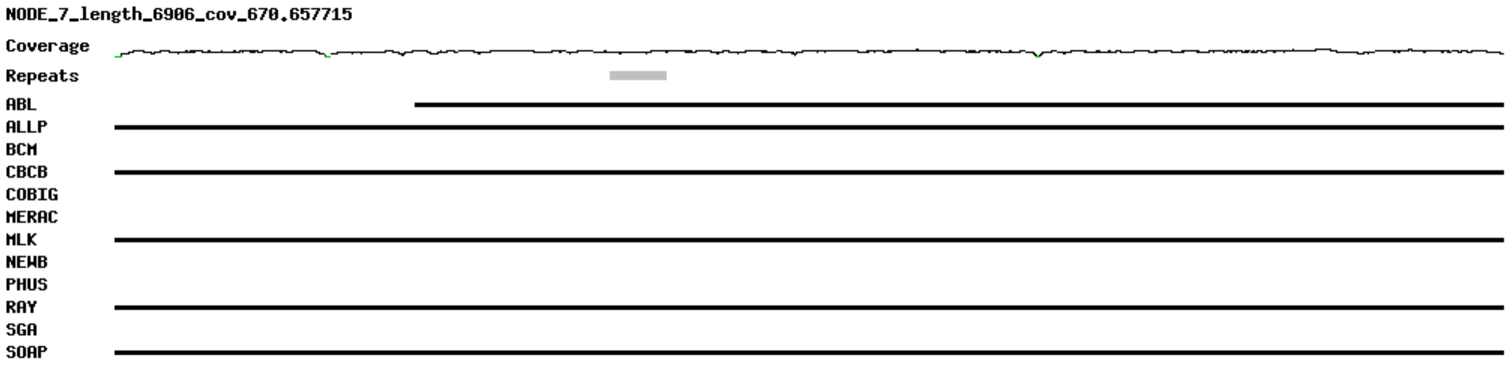
NODE_7_length_6906_cov_670.657715
Coverage
Repeats
ABL
ALLP
BCM
CBCB
COBIG
MERAC
MLK
NEWB
PHUS
RAY
SGA
SOAP

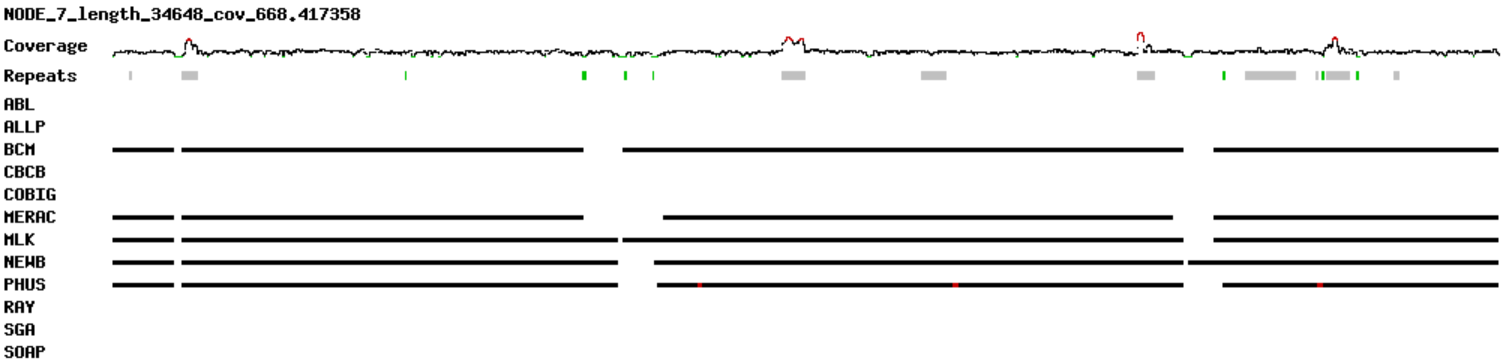
NODE_7_length_34648_cov_668.417358
Coverage
Repeats
ABL
ALLP
BCM
CBCB
COBIG
MERAC
MLK
NEWB
PHUS
RAY
SGA
SOAP

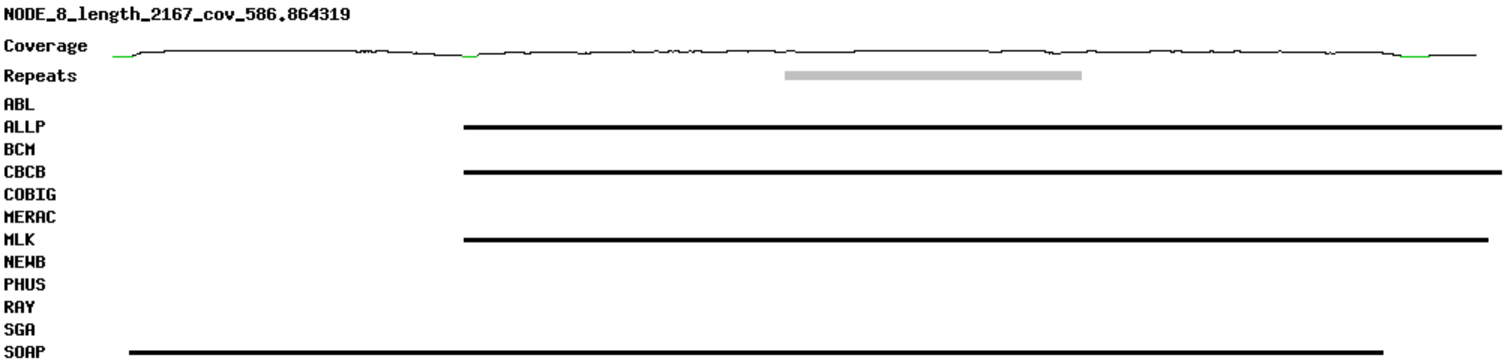
NODE_8_length_2167_cov_586.864319
Coverage
Repeats
ABL
ALLP
BCM
CBCB
COBIG
MERAC
MLK
NEWB
PHUS
RAY
SGA
SOAP

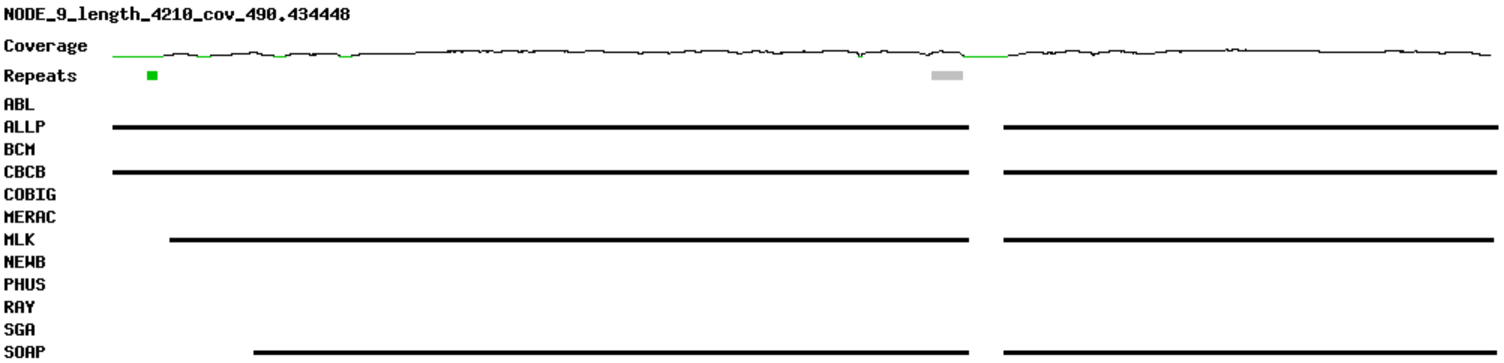
NODE_9_length_4210_cov_490.434448
Coverage
Repeats
ABL
ALLP
BCM
CBCB
COBIG
MERAC
MLK
NEWB
PHUS
RAY
SGA
SOAP

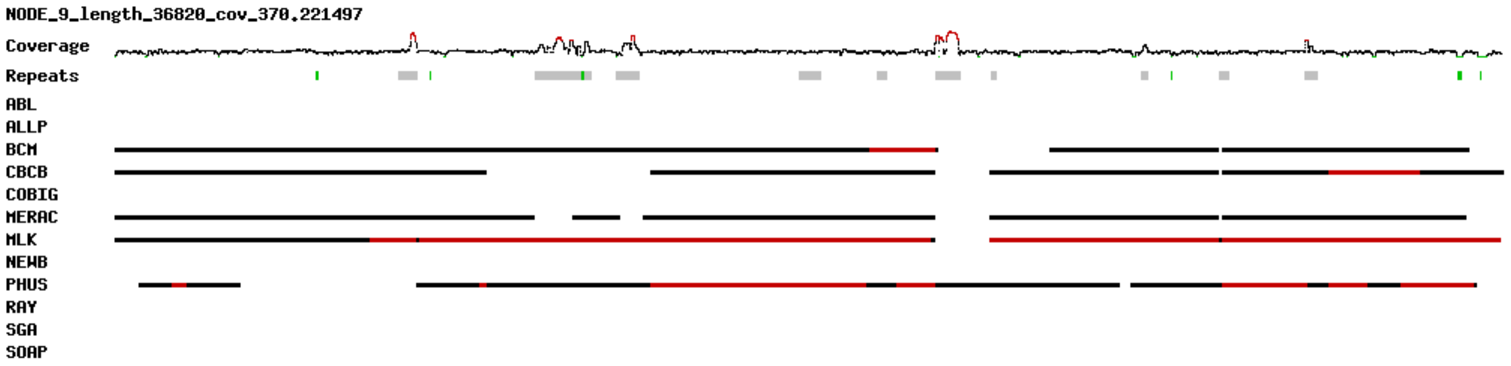
NODE_9_length_36820_cov_370.221497
Coverage
Repeats
ABL
ALLP
BCM
CBCB
COBIG
MERAC
MLK
NEWB
PHUS
RAY
SGA
SOAP

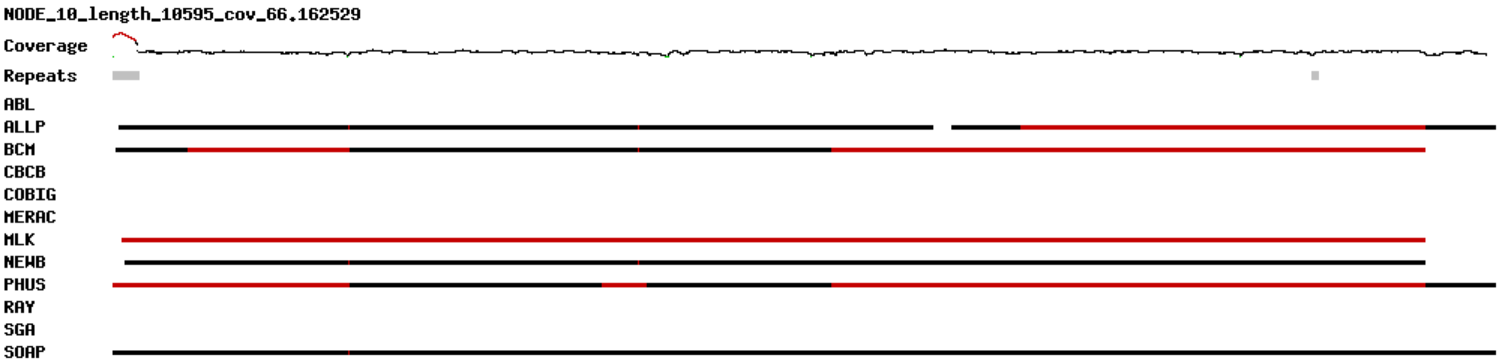
NODE_10_length_10595_cov_66.162529
Coverage
Repeats
ABL
ALLP
BCM
CBCB
COBIG
MERAC
MLK
NEWB
PHUS
RAY
SGA
SOAP

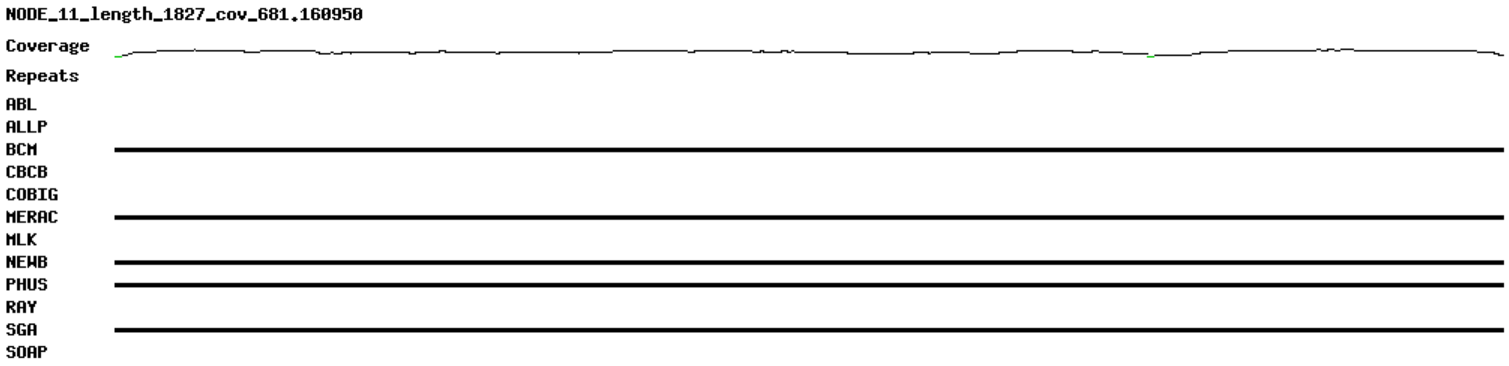
NODE_11_length_1827_cov_681.160950
Coverage
Repeats
ABL
ALLP
BCM
CBCB
COBIG
MERAC
MLK
NEWB
PHUS
RAY
SGA
SOAP

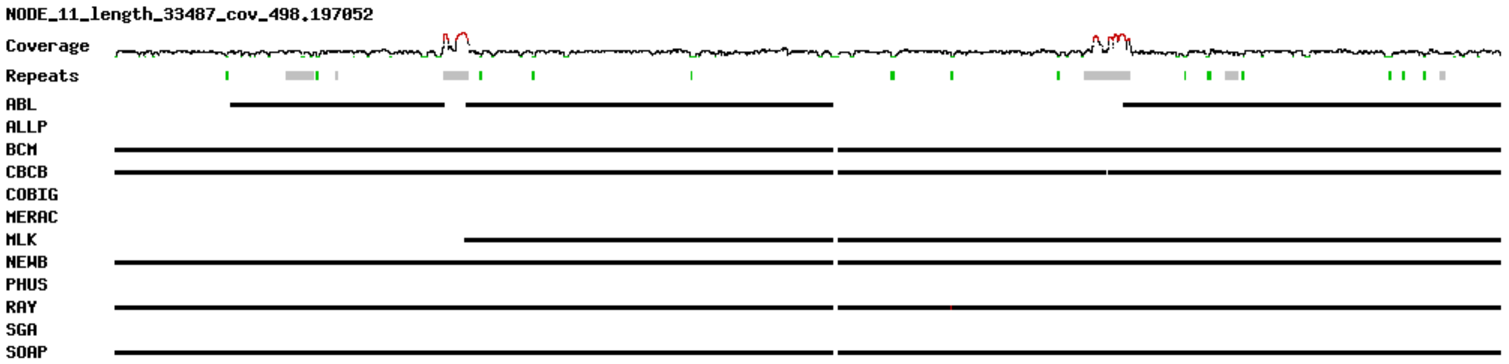
NODE_11_length_33487_cov_498.197052
Coverage
Repeats
ABL
ALLP
BCM
CBCB
COBIG
MERAC
MLK
NEWB
PHUS
RAY
SGA
SOAP

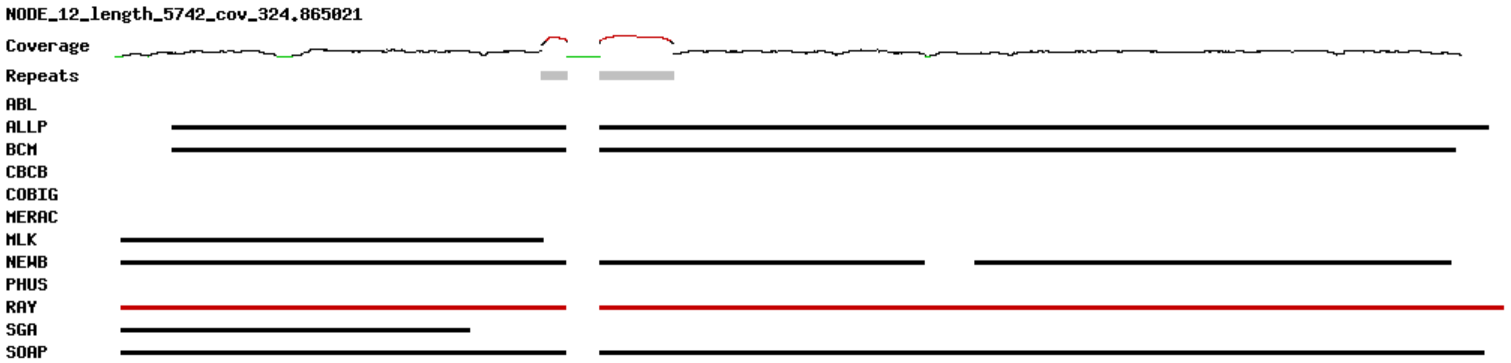
NODE_12_length_5742_cov_324.865021
Coverage
Repeats
ABL
ALLP
BCM
CBCB
COBIG
MERAC
MLK
NEWB
PHUS
RAY
SGA
SOAP

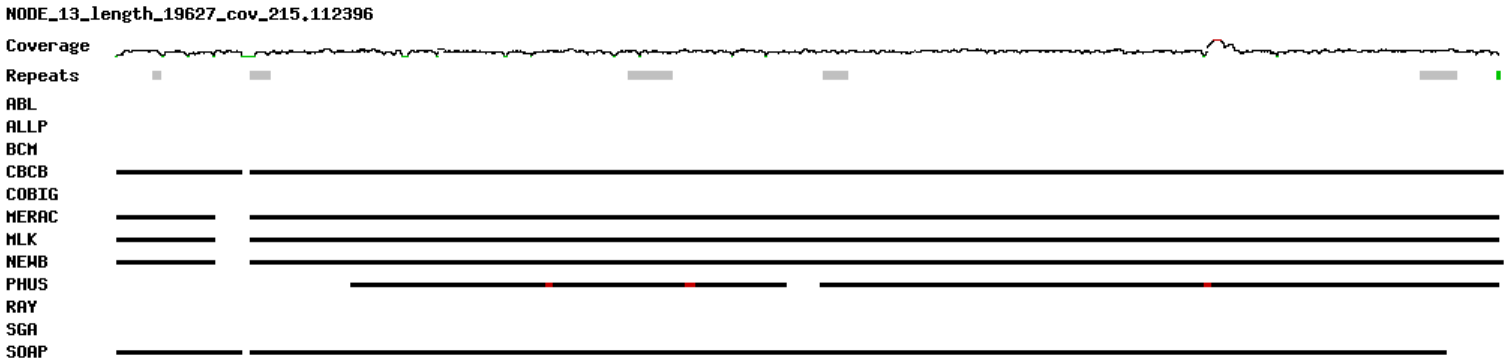
NODE_13_length_19627_cov_215.112396
Coverage
Repeats
ABL
ALLP
BCM
CBCB
COBIG
MERAC
MLK
NEWB
PHUS
RAY
SGA
SOAP

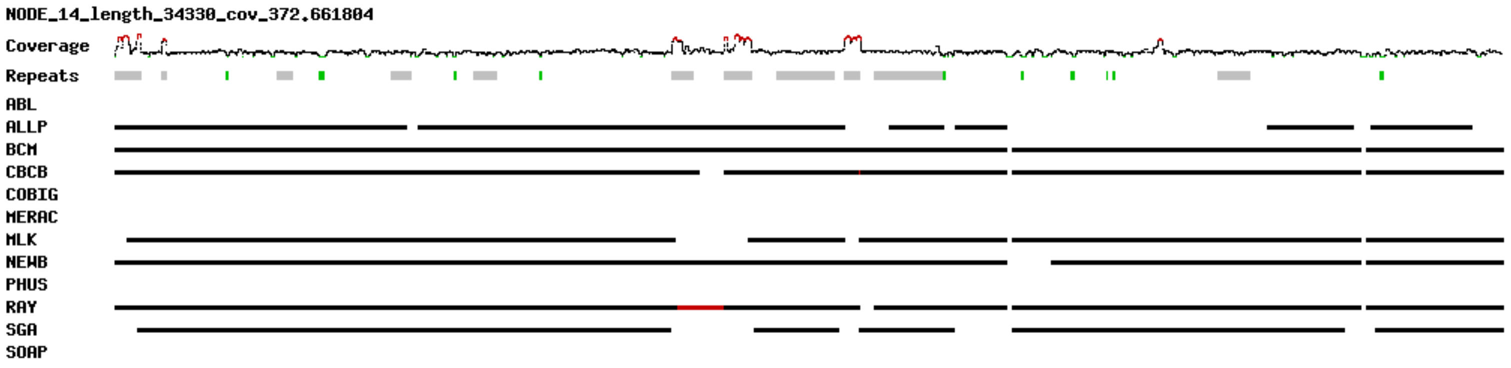
NODE_14_length_34330_cov_372.661804
Coverage
Repeats
ABL
ALLP
BCM
CBCB
COBIG
MERAC
MLK
NEWB
PHUS
RAY
SGA
SOAP

NODE_15_length_5020_cov_178.050598

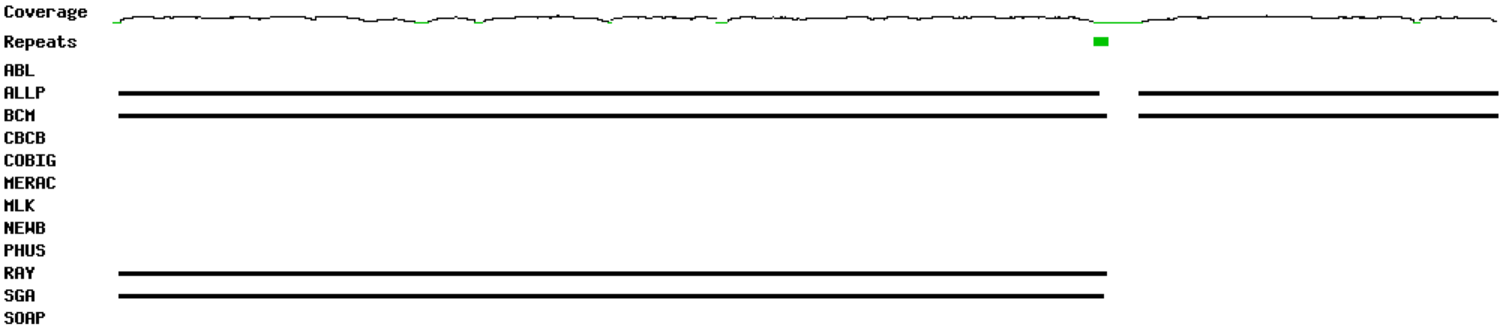

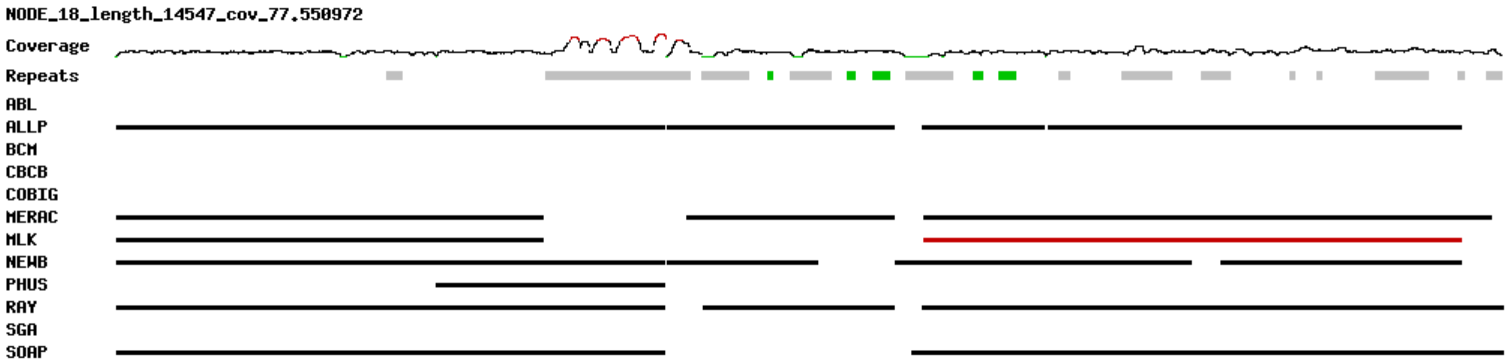
NODE_18_length_14547_cov_77.550972
Coverage
Repeats
ABL
ALLP
BCM
CBCB
COBIG
MERAC
MLK
NEWB
PHUS
RAY
SGA
SOAP

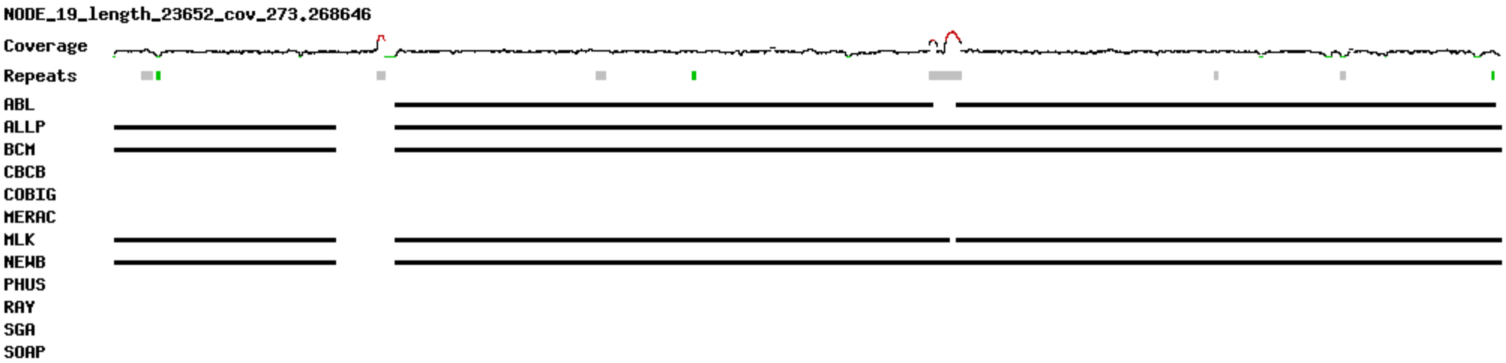
NODE_19_length_23652_cov_273.268646
Coverage
Repeats
ABL
ALLP
BCM
CBCB
COBIG
MERAC
MLK
NEWB
PHUS
RAY
SGA
SOAP

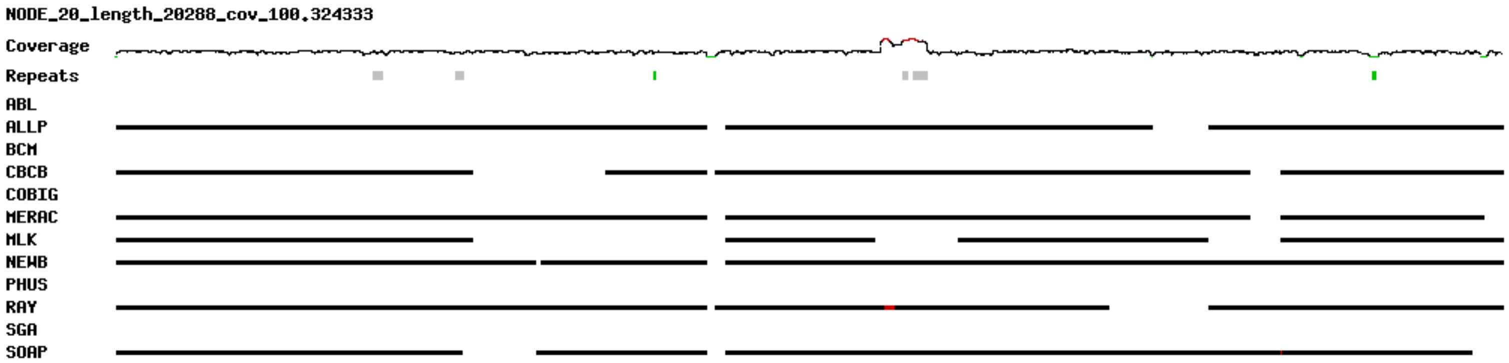
NODE_20_length_20288_cov_100.324333
Coverage
Repeats
ABL
ALLP
BCM
CBCB
COBIG
MERAC
MLK
NEWB
PHUS
RAY
SGA
SOAP

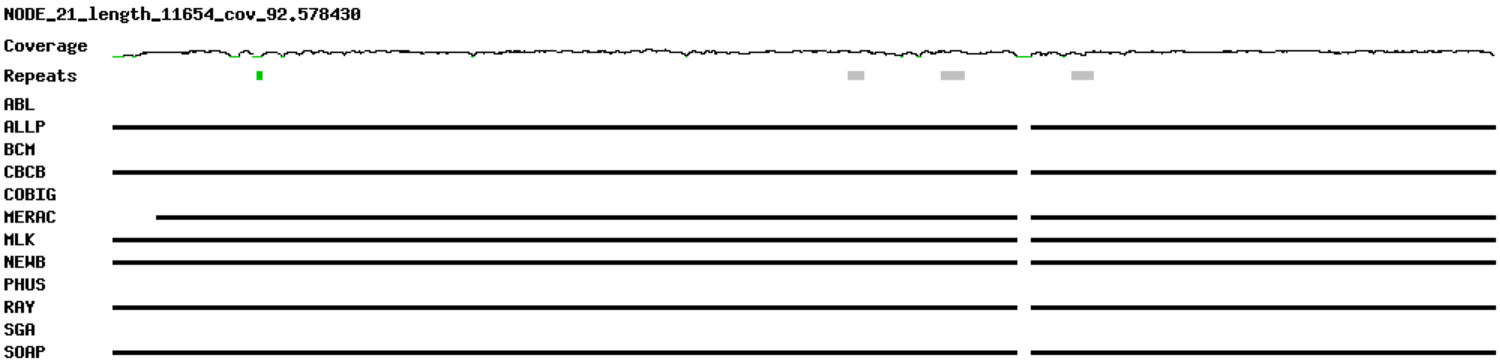
NODE_21_length_11654_cov_92.578430
Coverage
Repeats
ABL
ALLP
BCM
CBCB
COBIG
MERAC
MLK
NEWB
PHUS
RAY
SGA
SOAP

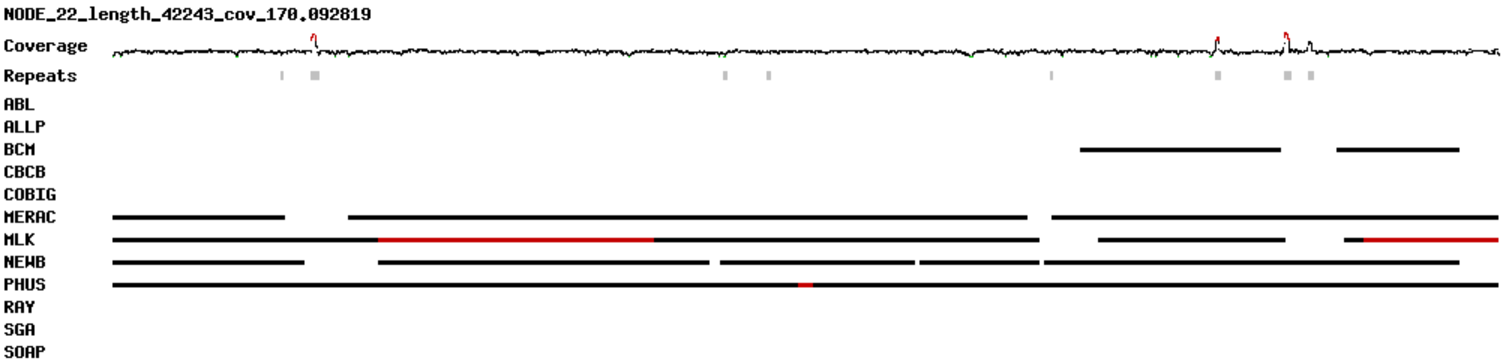
NODE_22_length_42243_cov_170.092819
Coverage
Repeats
ABL
ALLP
BCM
CBCB
COBIG
MERAC
MLK
NEWB
PHUS
RAY
SGA
SOAP

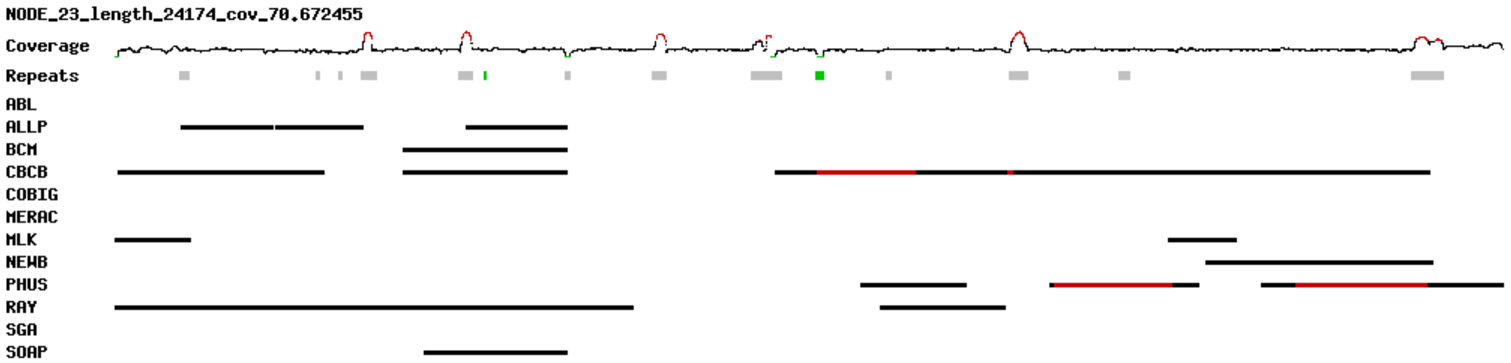
NODE_23_length_24174_cov_70.672455
Coverage
Repeats
ABL
ALLP
BCM
CBCB
COBIG
MERAC
MLK
NEWB
PHUS
RAY
SGA
SOAP

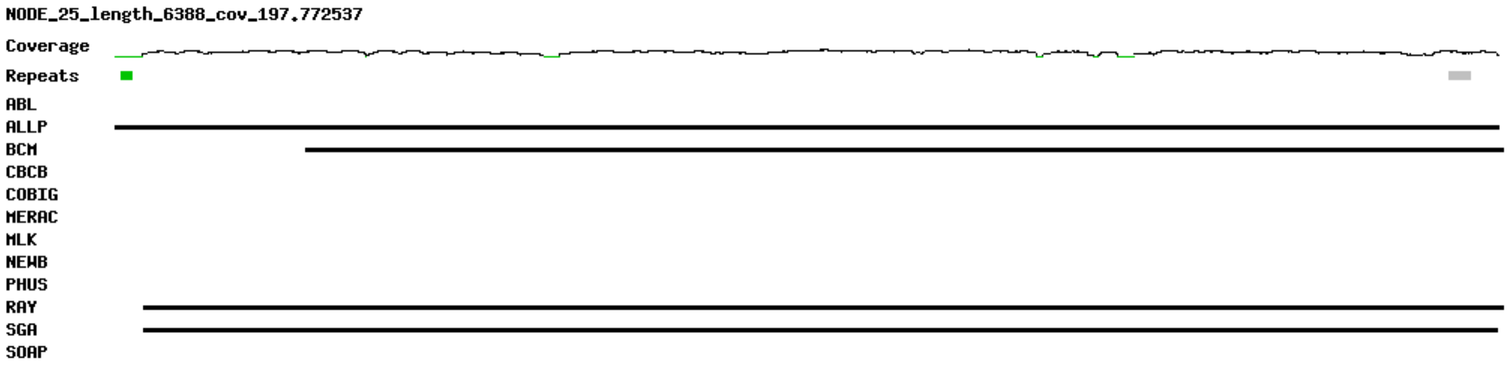
NODE_25_length_6388_cov_197.772537
Coverage
Repeats
ABL
ALLP
BCM
CBCB
COBIG
MERAC
MLK
NEWB
PHUS
RAY
SGA
SOAP

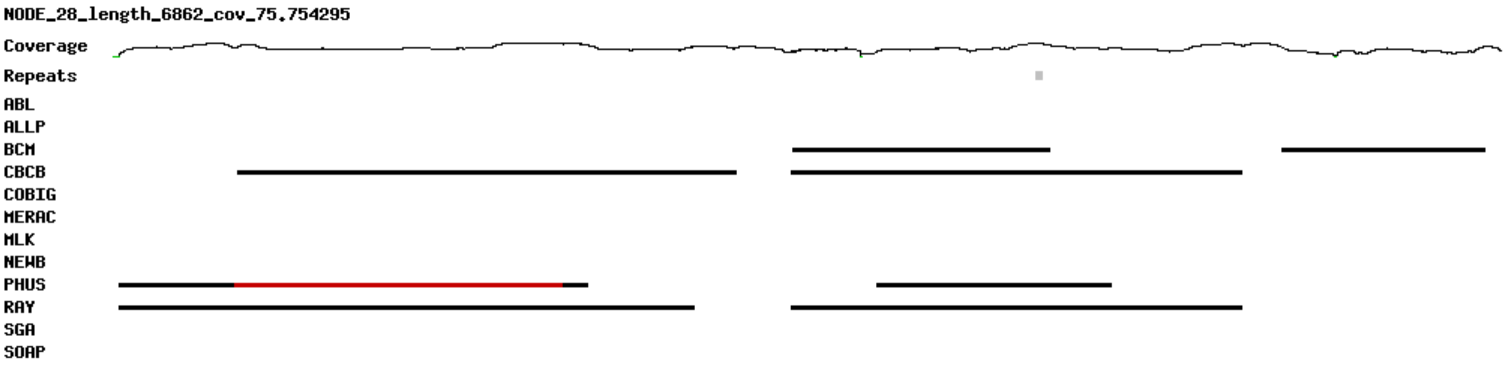

NODE_28_length_6862_cov_75.754295
Coverage
Repeats
ABL
ALLP
BCM
CBCB
COBIG
MERAC
MLK
NEWB
PHUS
RAY
SGA
SOAP

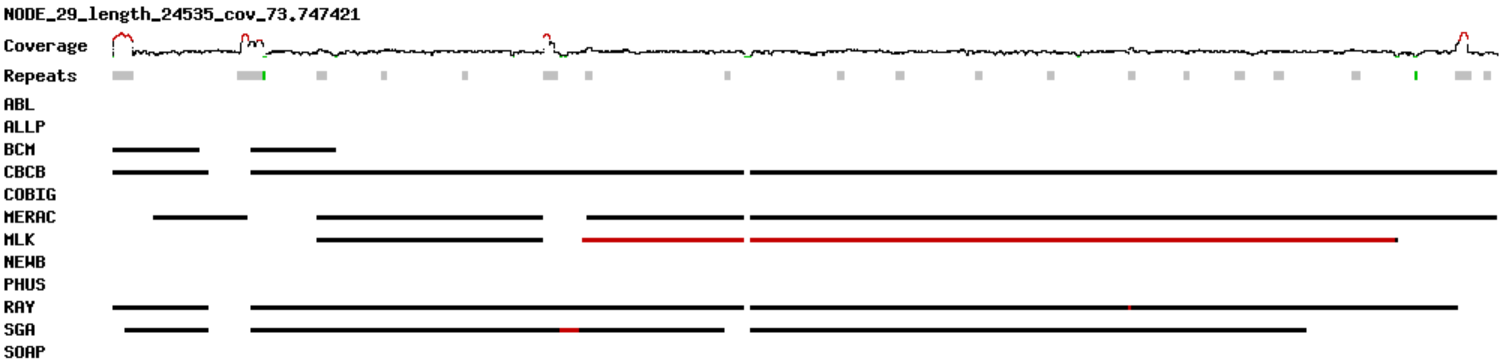

NODE_29_length_24535_cov_73.747421
Coverage
Repeats
ABL
ALLP
BCM
CBCB
COBIG
MERAC
MLK
NEWB
PHUS
RAY
SGA
SOAP

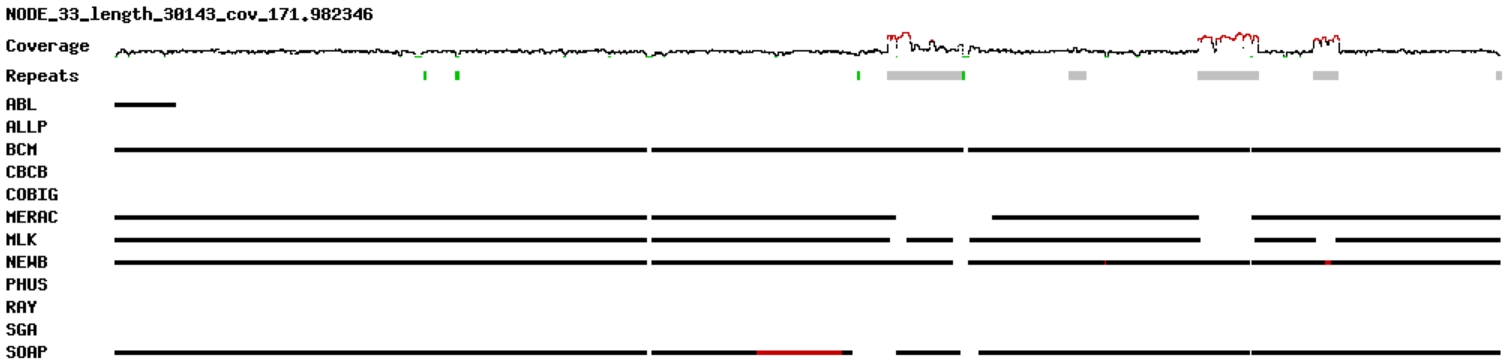

NODE_33_length_30143_cov_171.982346
Coverage
Repeats
ABL
ALLP
BCM
CBCB
COBIG
MERAC
MLK
NEWB
PHUS
RAY
SGA
SOAP

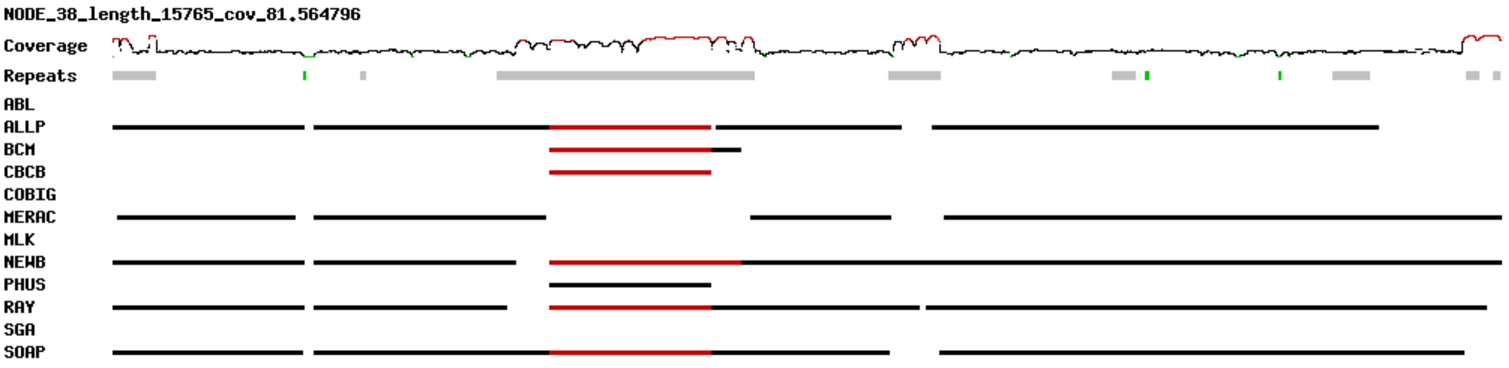
NODE_38_length_15765_cov_81.564796
Coverage
Repeats
ABL
ALLP
BCM
CBCB
COBIG
MERAC
MLK
NEWB
PHUS
RAY
SGA
SOAP

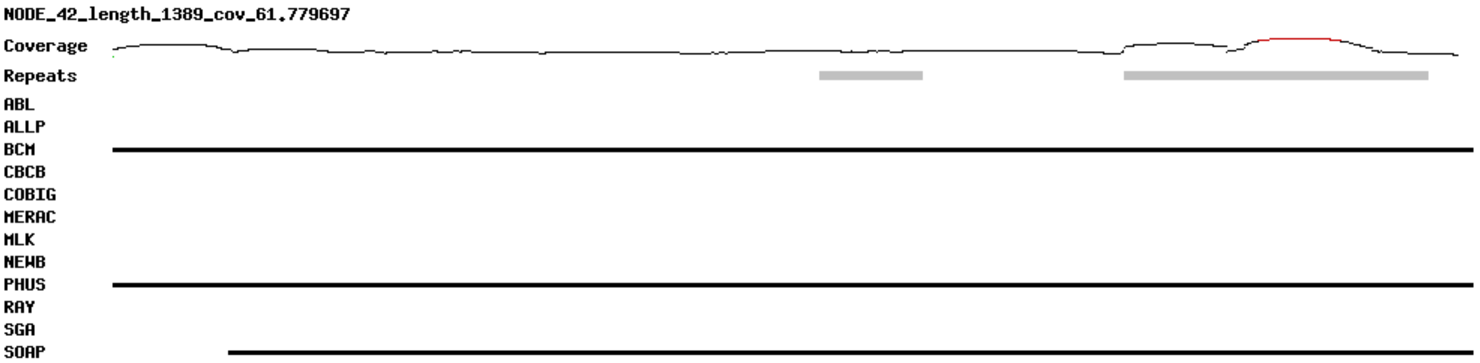
NODE_42_length_1389_cov_61.779697
Coverage
Repeats
ABL
ALLP
BCM
CBCB
COBIG
MERAC
MLK
NEWB
PHUS
RAY
SGA
SOAP

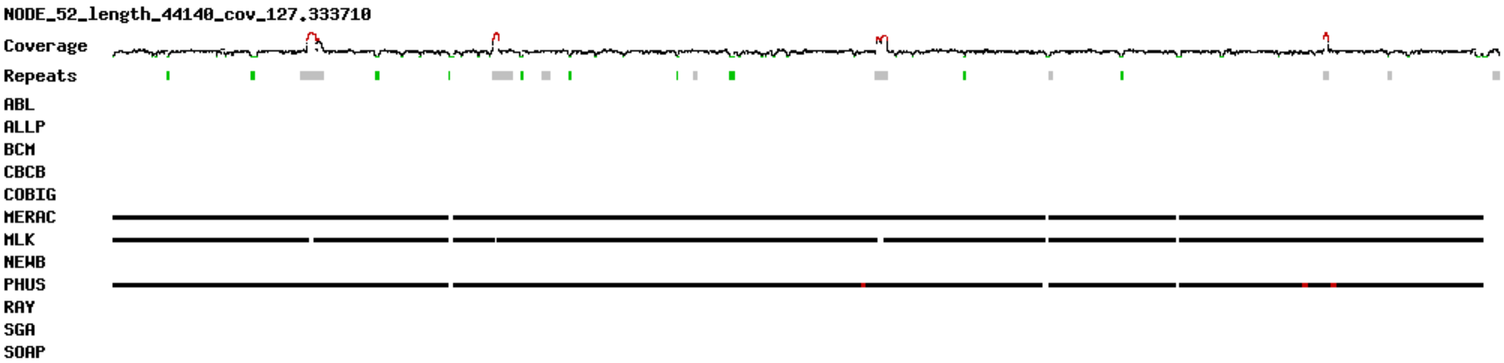
NODE_52_length_44140_cov_127.333710
Coverage
Repeats
ABL
ALLP
BCM
CBCB
COBIG
MERAC
MLK
NEWB
PHUS
RAY
SGA
SOAP
