# Supplementary material for: Assemblathon 2: evaluating de novo methods of genome assembly in three vertebrate species
Source: Gigascience. 2013 Jul 22;2:10. doi: 10.1186/2047-217X-2-10 (PMC3844414; doi:10.1186/2047-217X-2-10)
Supplement: Additional file 8 — Snake scaffolds mapped to snake Fosmids. Results of using BLAST to align 24 assembled Fosmid sequences to snake scaffold sequences. Each figure represents an assembled Fosmid sequence with tracks showing read coverage, presence of repeats, and alignments to each assembly. [file 2047-217X-2-10-S8.pdf]

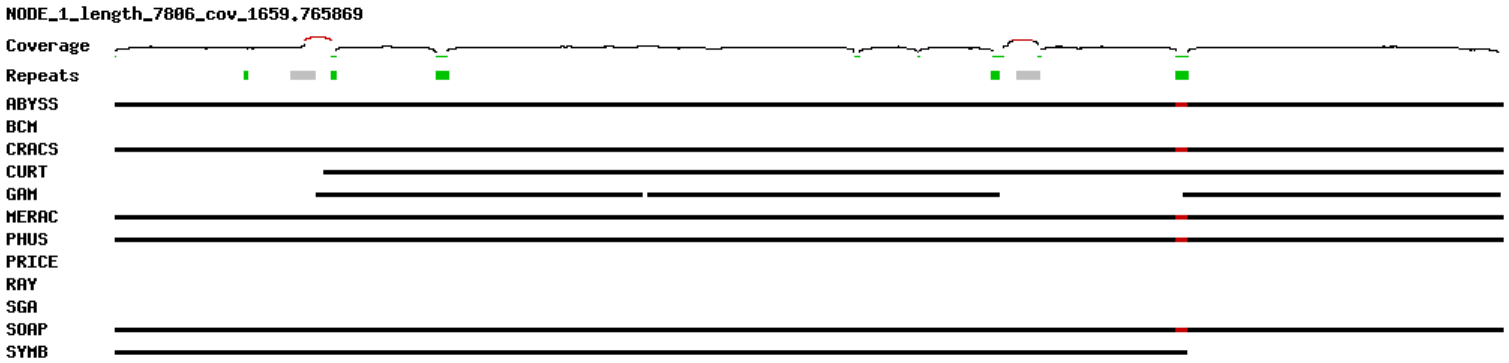
NODE_1_length_7806_cov_1659.765869
Coverage
Repeats
ABYSS
BCM
CRACS
CURT
GAM
MERAC
PHUS
PRICE
RAY
SGA
SOAP
SYMB

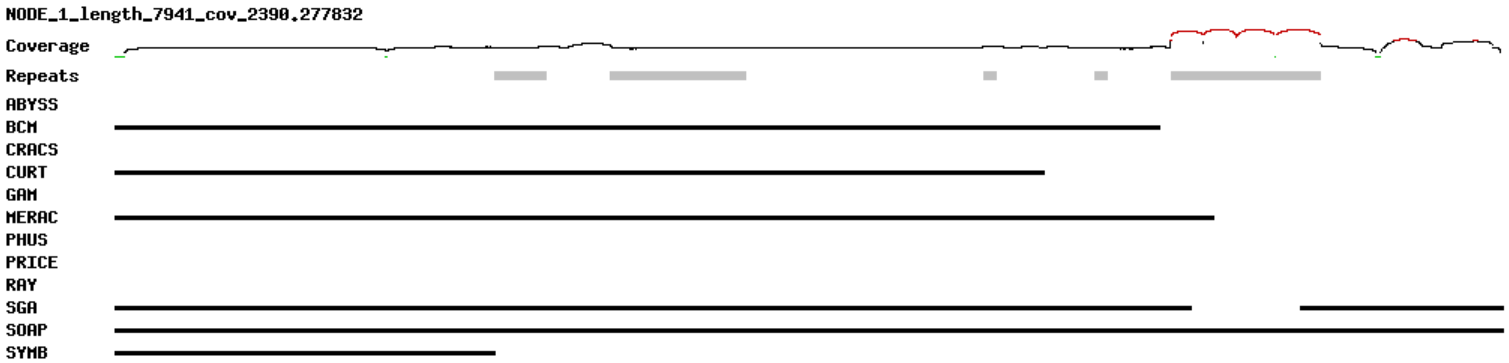
NODE_1_length_7941_cov_2390.277832
Coverage
Repeats
ABYSS
BCM
CRACS
CURT
GAM
MERAC
PHUS
PRICE
RAY
SGA
SOAP
SYMB

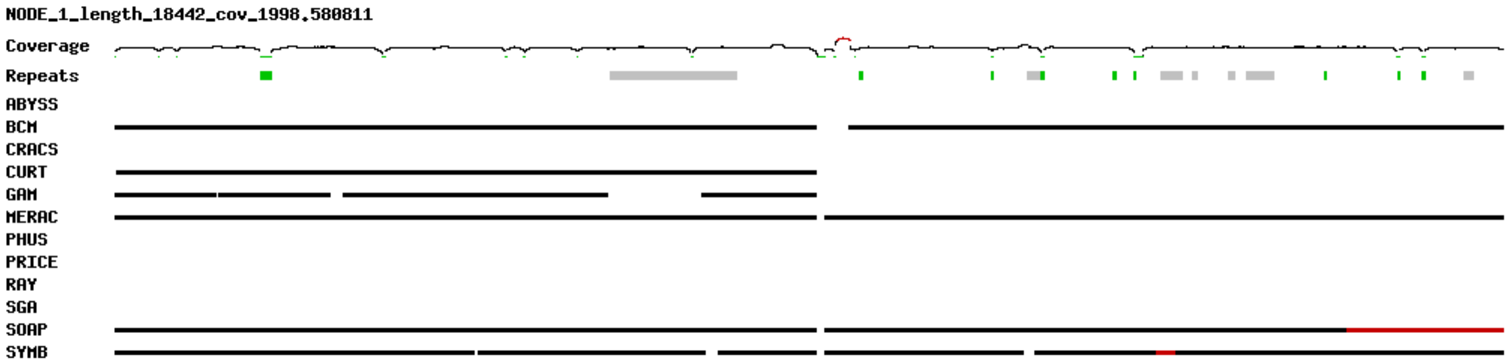
NODE_1_length_18442_cov_1998.580811
Coverage
Repeats
ABYSS
BCM
CRACS
CURT
GAM
MERAC
PHUS
PRICE
RAY
SGA
SOAP
SYMB

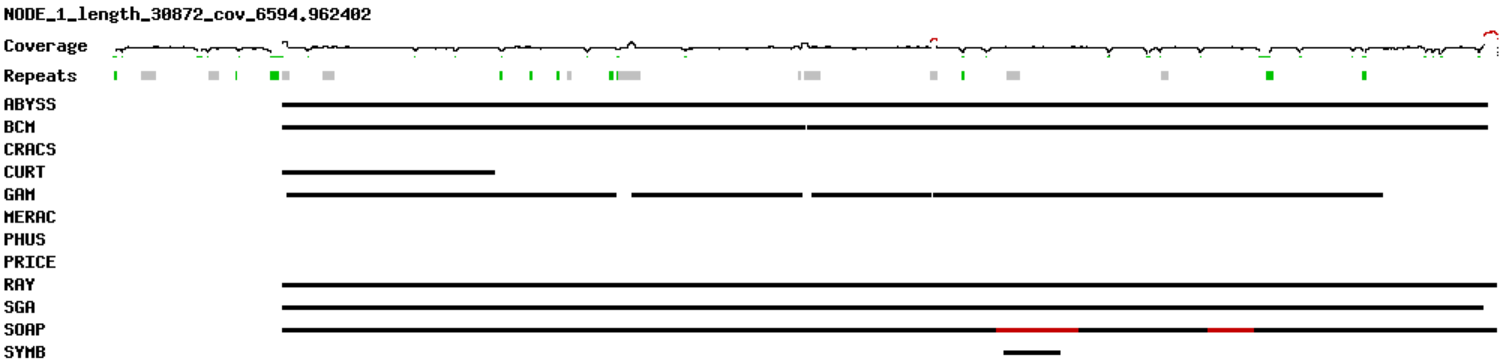
NODE_1_length_30872_cov_6594.962402
Coverage
Repeats
ABYSS
BCM
CRACS
CURT
GAM
MERAC
PHUS
PRICE
RAY
SGA
SOAP
SYMB

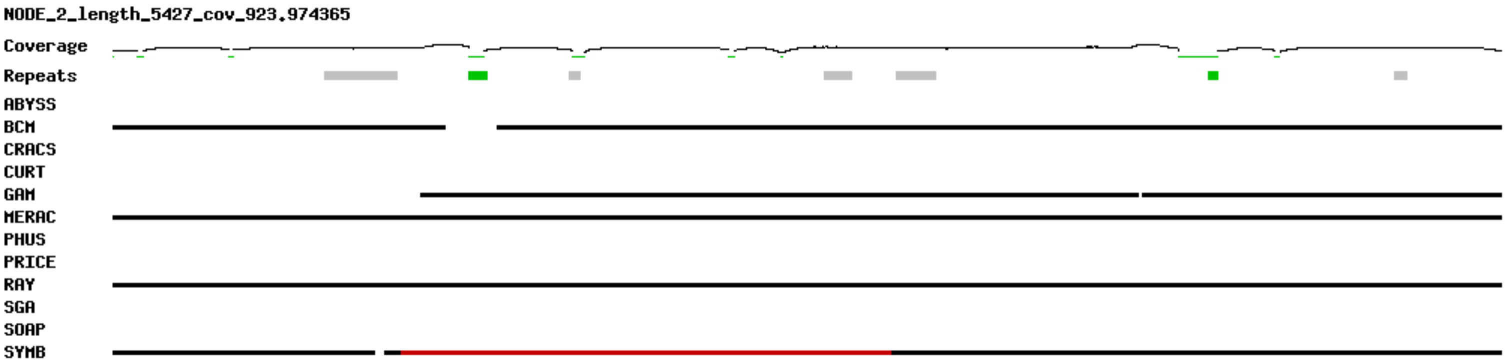
NODE_2_length_5427_cov_923.974365
Coverage
Repeats
ABYSS
BCM
CRACS
CURT
GAM
MERAC
PHUS
PRICE
RAY
SGA
SOAP
SYMB

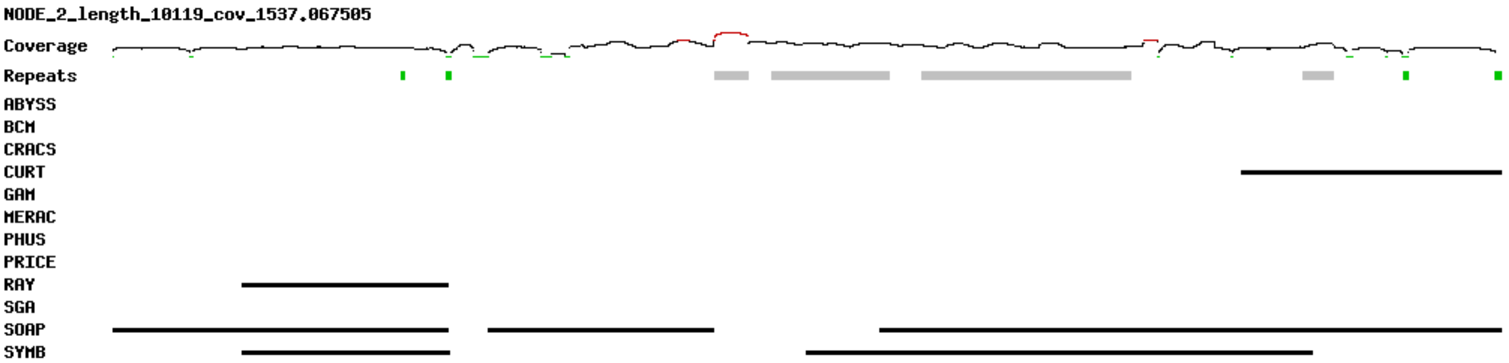
NODE_2_length_10119_cov_1537.067505
Coverage
Repeats
ABYSS
BCM
CRACS
CURT
GAM
MERAC
PHUS
PRICE
RAY
SGA
SOAP
SYMB

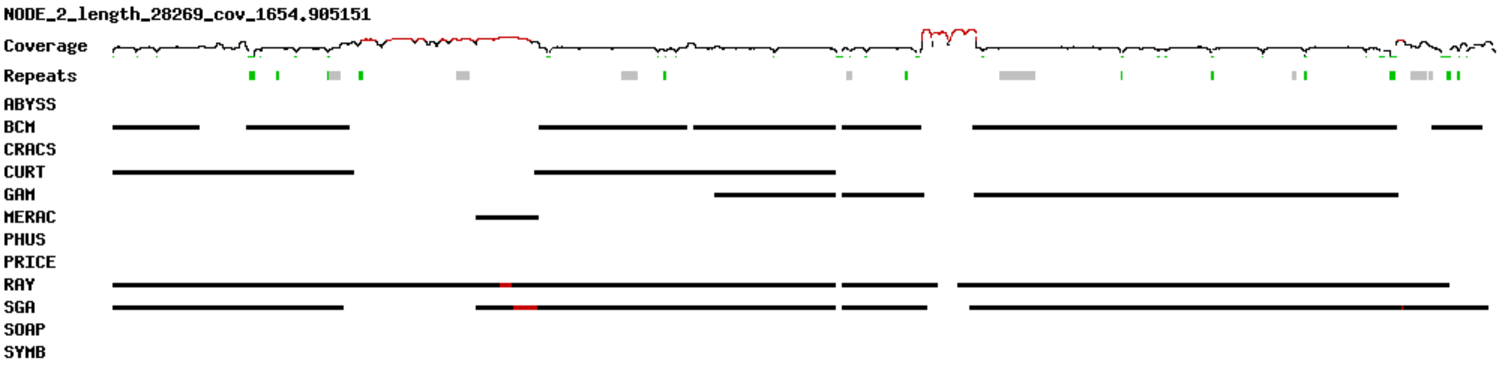
NODE_2_length_28269_cov_1654.905151
Coverage
Repeats
ABYSS
BCM
CRACS
CURT
GAM
MERAC
PHUS
PRICE
RAY
SGA
SOAP
SYMB

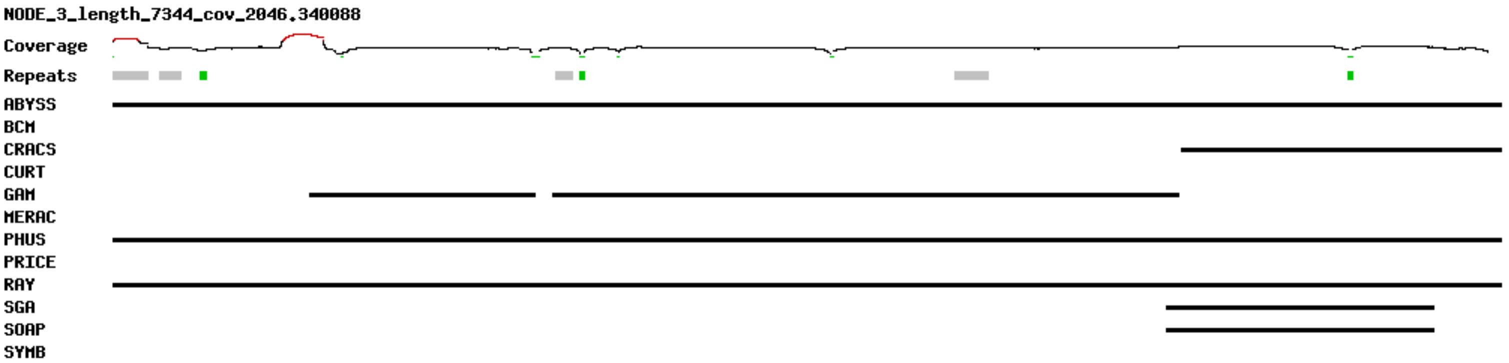
NODE_3_length_7344_cov_2046.340088
Coverage
Repeats
ABYSS
BCM
CRACS
CURT
GAM
MERAC
PHUS
PRICE
RAY
SGA
SOAP
SYMB

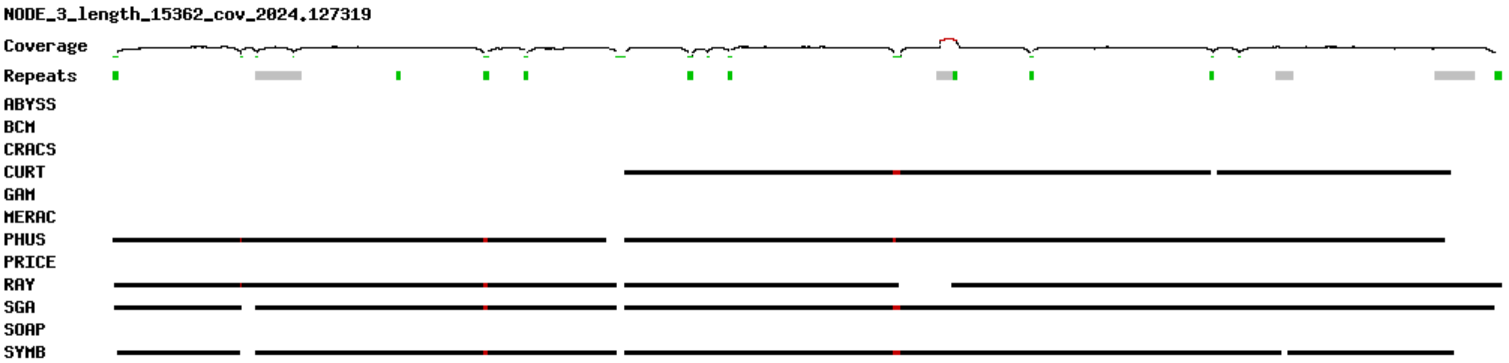
NODE_3_length_15362_cov_2024.127319
Coverage
Repeats
ABYSS
BCM
CRACS
CURT
GAM
MERAC
PHUS
PRICE
RAY
SGA
SOAP
SYMB

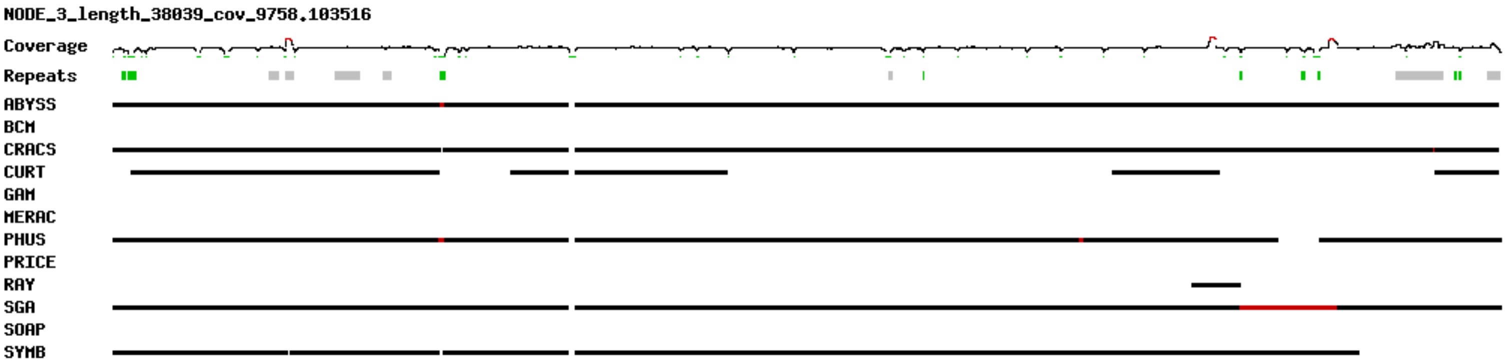
NODE_3_length_38039_cov_9758.103516
Coverage
Repeats
ABYSS
BCM
CRACS
CURT
GAM
MERAC
PHUS
PRICE
RAY
SGA
SOAP
SYMB

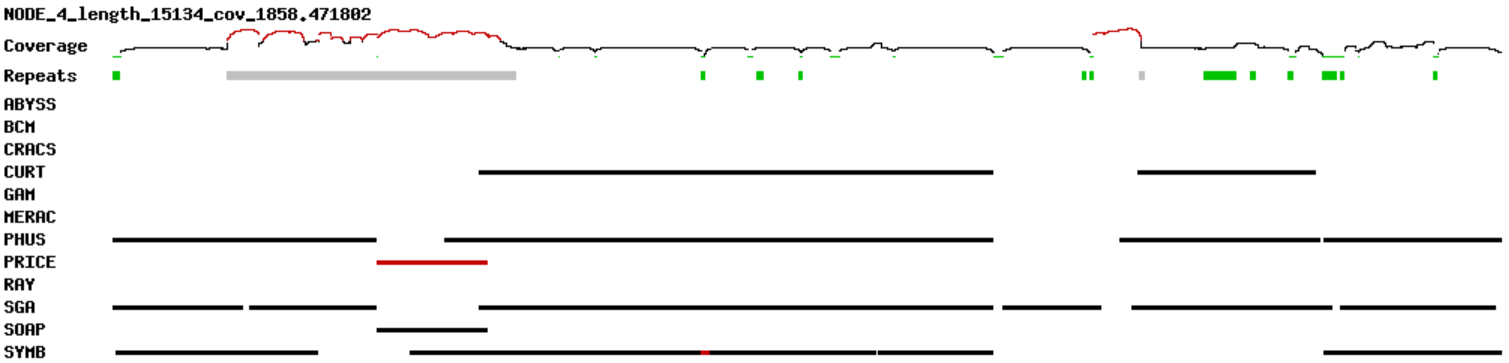
NODE_4_length_15134_cov_1858.471802
Coverage
Repeats
ABYSS
BCM
CRACS
CURT
GAM
MERAC
PHUS
PRICE
RAY
SGA
SOAP
SYMB

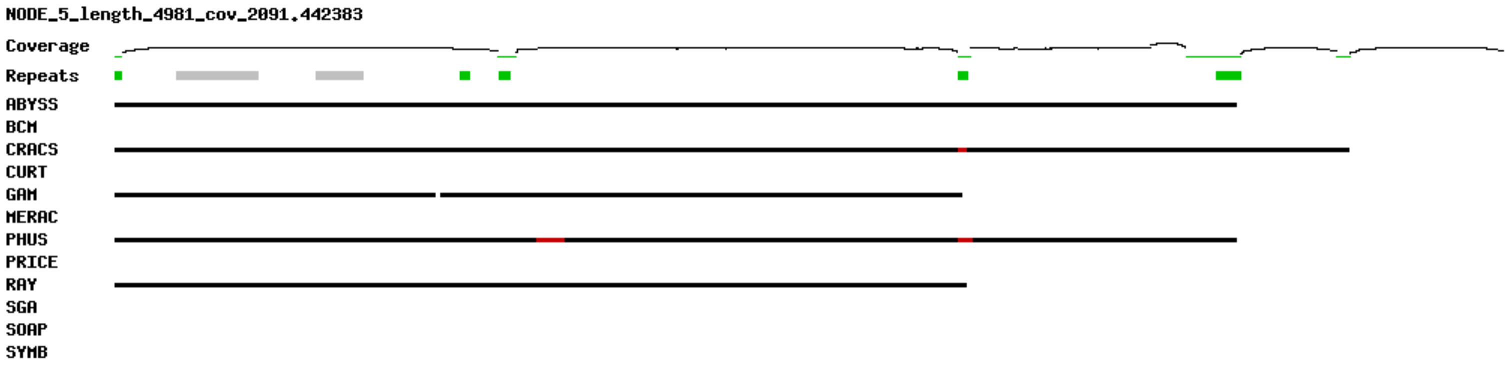
NODE_5_length_4981_cov_2091.442383
Coverage
Repeats
ABYSS
BCM
CRACS
CURT
GAM
MERAC
PHUS
PRICE
RAY
SGA
SOAP
SYMB

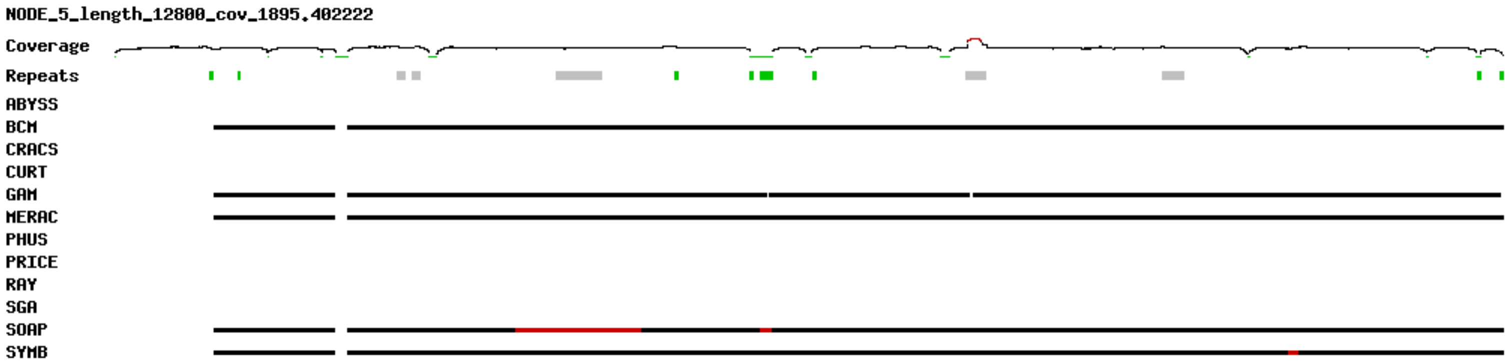
NODE_5_length_12800_cov_1895.402222
Coverage
Repeats
ABYSS
BCM
CRACS
CURT
GAM
MERAC
PHUS
PRICE
RAY
SGA
SOAP
SYMB

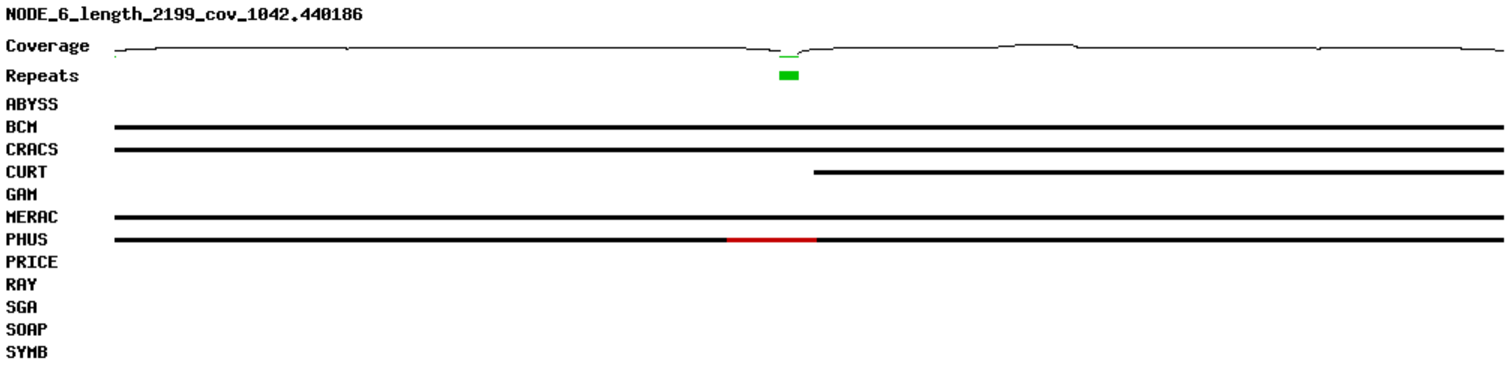
NODE_6_length_2199_cov_1042.440186
Coverage
Repeats
ABYSS
BCM
CRACS
CURT
GAM
MERAC
PHUS
PRICE
RAY
SGA
SOAP
SYMB

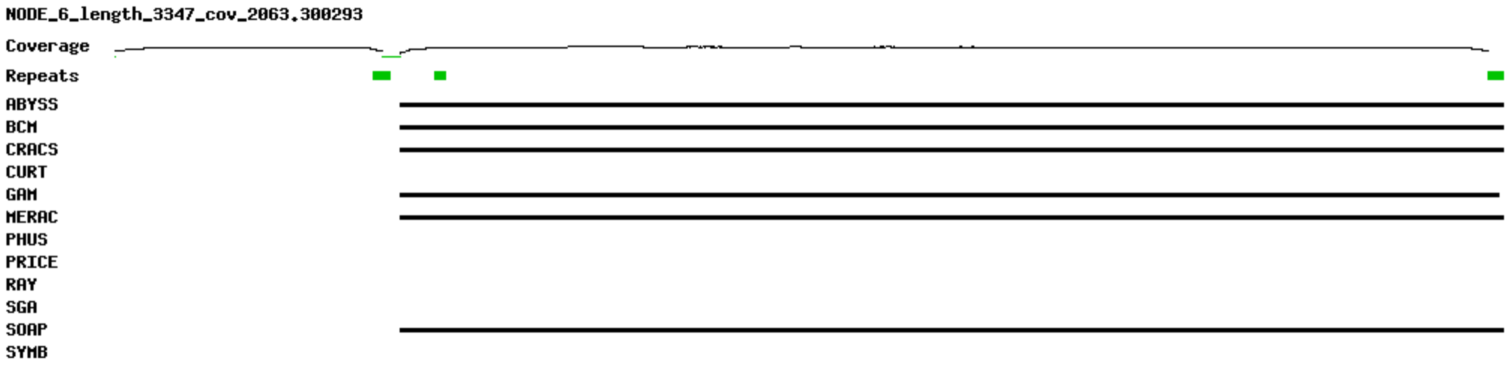
NODE_6_length_3347_cov_2063.300293
Coverage
Repeats
ABYSS
BCM
CRACS
CURT
GAM
MERAC
PHUS
PRICE
RAY
SGA
SOAP
SYMB

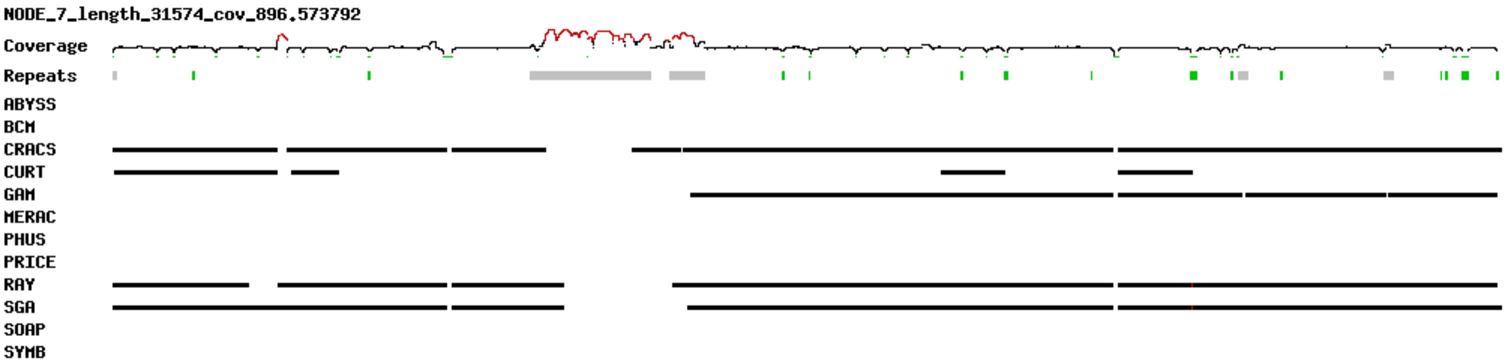
NODE_7_length_31574_cov_896.573792
Coverage
Repeats
ABYSS
BCM
CRACS
CURT
GAM
MERAC
PHUS
PRICE
RAY
SGA
SOAP
SYMB

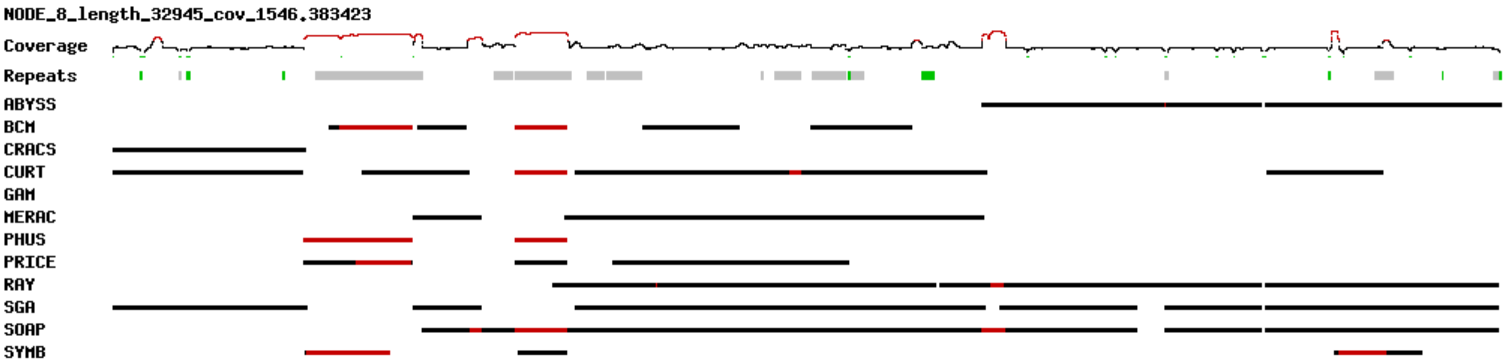
NODE_8_length_32945_cov_1546.383423
Coverage
Repeats
ABYSS
BCM
CRACS
CURT
GAM
MERAC
PHUS
PRICE
RAY
SGA
SOAP
SYMB

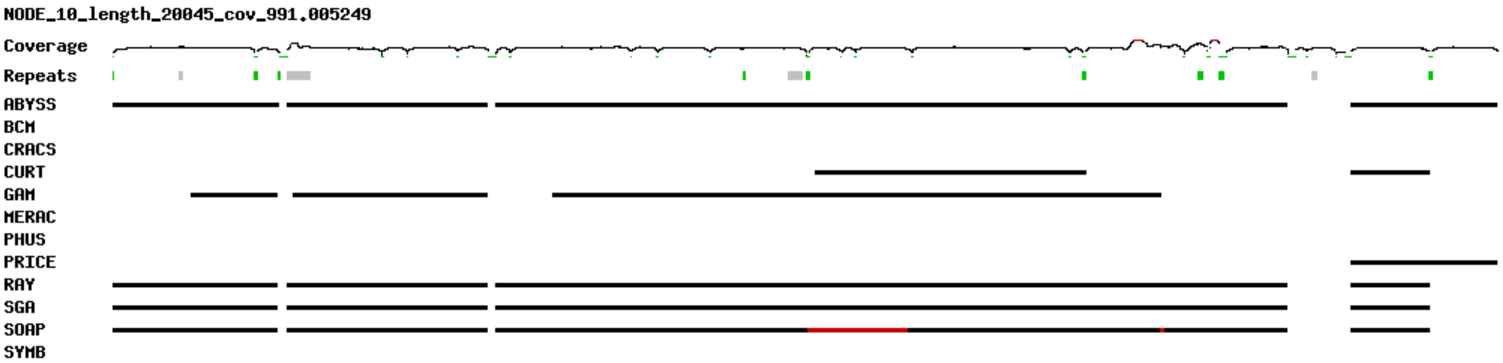
NODE_10_length_20045_cov_991.005249
Coverage
Repeats
ABYSS
BCM
CRACS
CURT
GAM
MERAC
PHUS
PRICE
RAY
SGA
SOAP
SYMB

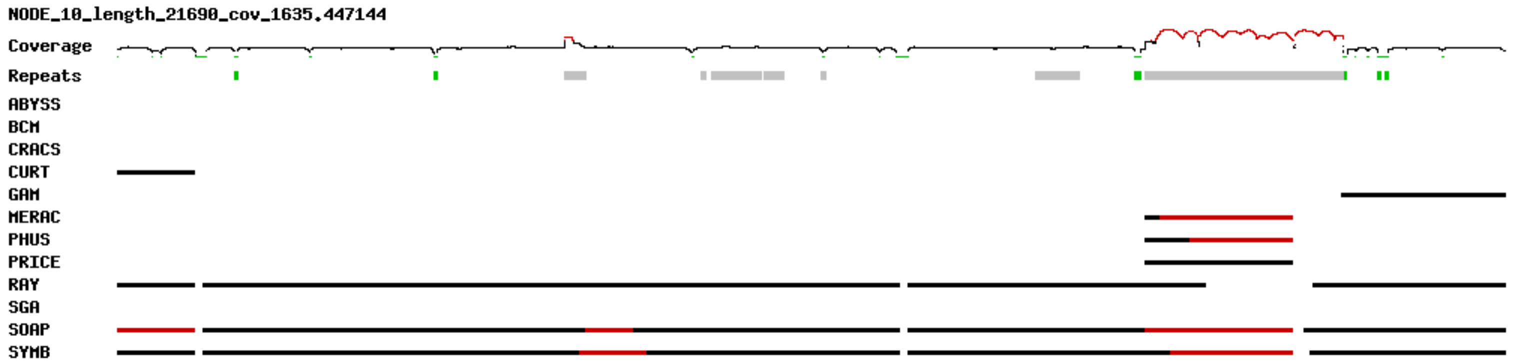
NODE_10_length_21690_cov_1635.447144
Coverage
Repeats
ABYSS
BCM
CRACS
CURT
GAM
MERAC
PHUS
PRICE
RAY
SGA
SOAP
SYMB

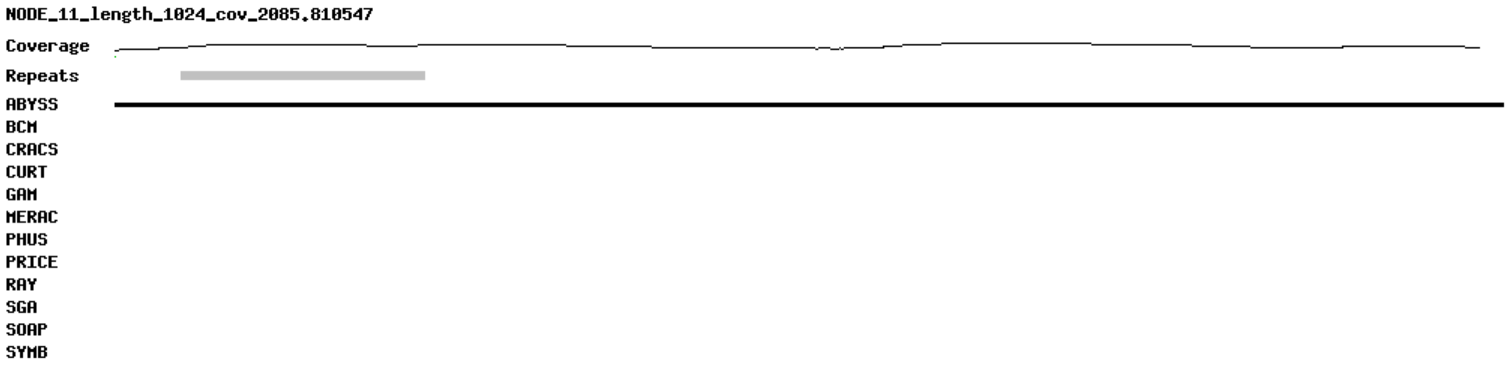
NODE_11_length_1024_cov_2085.810547
Coverage
Repeats
ABYSS
BCM
CRACS
CURT
GAM
MERAC
PHUS
PRICE
RAY
SGA
SOAP
SYMB

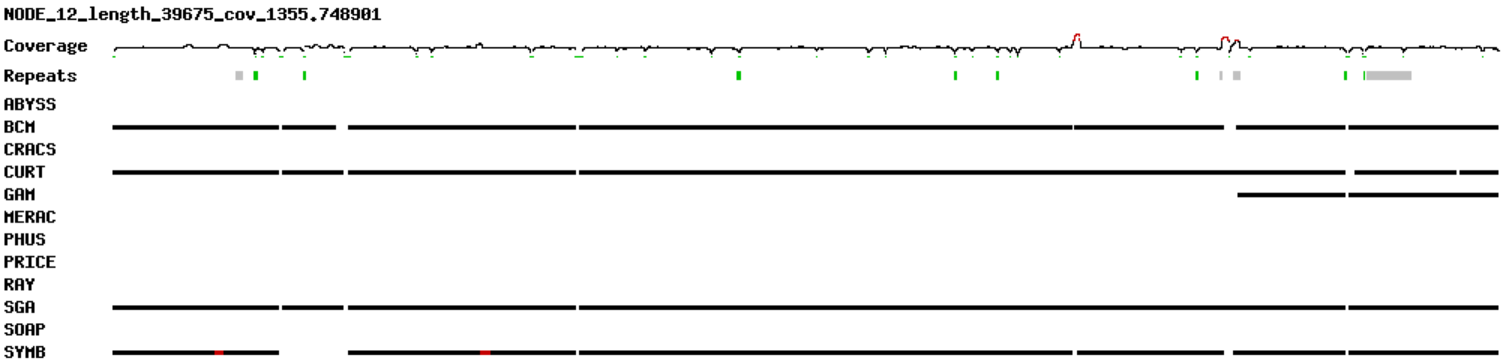
NODE_12_length_39675_cov_1355.748901
Coverage
Repeats
ABYSS
BCM
CRACS
CURT
GAM
MERAC
PHUS
PRICE
RAY
SGA
SOAP
SYMB

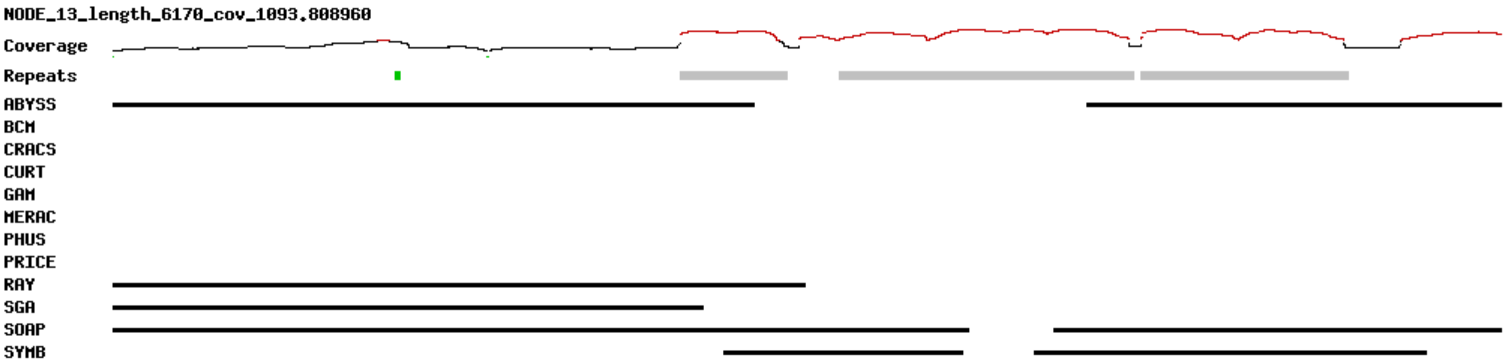
NODE_13_length_6170_cov_1093.808960
Coverage
Repeats
ABYSS
BCM
CRACS
CURT
GAM
MERAC
PHUS
PRICE
RAY
SGA
SOAP
SYMB

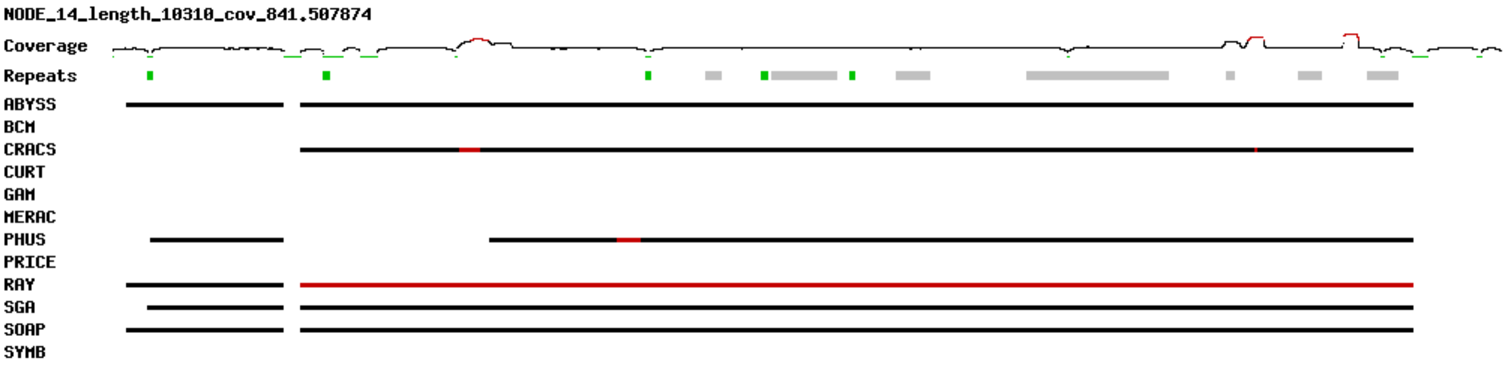
NODE_14_length_10310_cov_841.507874
Coverage
Repeats
ABYSS
BCM
CRACS
CURT
GAM
MERAC
PHUS
PRICE
RAY
SGA
SOAP
SYMB

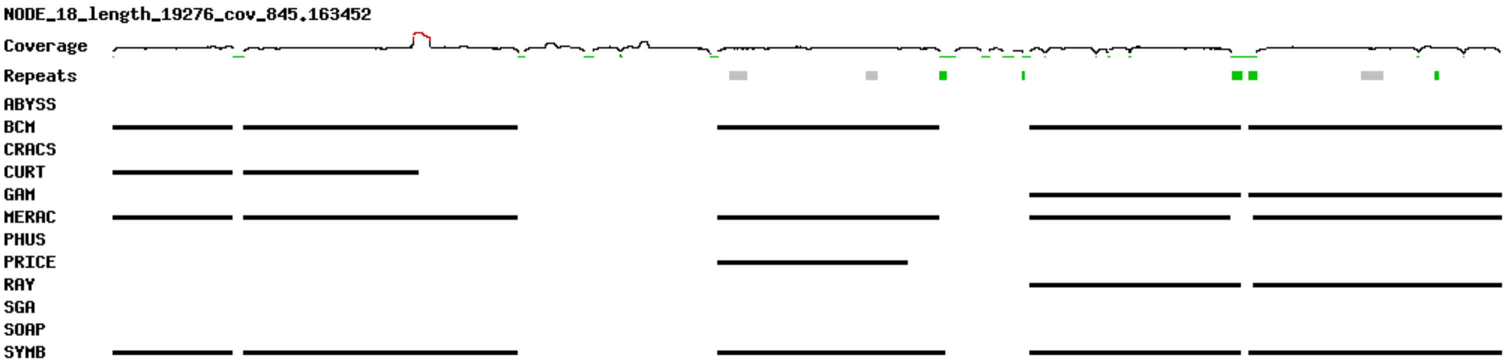
NODE_18_length_19276_cov_845.163452
Coverage
Repeats
ABYSS
BCM
CRACS
CURT
GAM
MERAC
PHUS
PRICE
RAY
SGA
SOAP
SYMB
